# Supplementary material for: Blueprinting extendable nanomaterials with standardized protein blocks
Source: Nature. 2024 Mar 13;627(8005):898–904. doi: 10.1038/s41586-024-07188-4 (PMC10972742; doi:10.1038/s41586-024-07188-4)
Supplement: Supplementary file 1 — Supplementary Methods, Discussion, Figs. 1–43 and Tables 1–6. [file 41586_2024_7188_MOESM1_ESM.pdf]

nature portfolio

https://doi.org/10.1038/s41586-024-07188-4

Supplementary information

Blueprinting extendable nanomaterials with standardized protein blocks

In the format provided by the authors and unedited

Supplementary Methods for *Blueprinting extendable nanomaterials with standardized protein blocks*

Computational design

Placement of straight alpha helices for THRs (both linear and curved) and SHDs

The Rosetta protein design software suite has an implementation of Crick-parameter [16] generated secondary structures. The MakeBundle or BundleGridSampler movers in RosettaScripts [18] can both be used to generate preset idealized helices. We use both the "alpha_helix" and "alpha_helix_100" parameter presets; if blocks will be combined by fusion later, then it could be beneficial to use the same parameter sets for all blocks. The "alpha_helix_100" preset also features the repeating helical phase every 18 residues, so structures can be modularly lengthened or shortened at the level of helix length (fig. S4).

Both movers offer functionality for placing helices in the X-Y plane with specific phase and position. For a simple THR design, it is easiest to place an initial helix at the origin, and then place other helices relative to that one. We find that distances from the center of 1 helix to the center of an adjacent helix can be in the range of 8.5 Å to 11.5 Å to offer favorable packing solutions between them. The most favorable spacing to find solutions seems to be near 10.5 Å. Sampling the phases of adjacent helices is primarily how different packing solutions are found (although sometimes DOFs are locked because distance/phase relationships are used to establish repeat trajectory). It is also reasonable to use Python/PyRosetta [36] to position copies of a template ideal helix.

For making a repeat protein, all helices of the first repeat unit plus one copy of the first helix are generated with explicitly defined positioning. Then, the ConnectChainsMover is used to install loops between helices using helix-loop-helix structural fragments (available for download at: files.ipd.uw.edu/pub/modular_repeat_protein_2020/ss_grouped_vall_all.h5) that were harvested from the PDB[17]. Once this looped "repeat unit +1" structure is made, then the RepeatPropagationMover is used to generate perfect repeat structures that contain however many repeats are desired.

An example script for this building block generation is provided at https://github.com/tfhuddy/2023-manuscript-materials. This example uses a dummy input to an annotated RosettaScripts xml script, which will be used with the Rosetta executable to produce a ***Linear*** THR backbone.

Sequence design on protein backbones

THR1-THR14 were sequence-designed with RosettaScripts, using the FastDesign protocol. To avoid high alanine content and voids due to rigid backbone design, small deviations in the backbone were allowed during FastDesign, but limited by using constraints to maintain backbone ideality along repeats.

As ProteinMPNN has benchmarked better both in success rate and computational efficiency generally, we found ProteinMPNN to be sufficient for all later sequence design operations[20]. For repeat proteins and homomeric oligomers, it is possible to restrict sequences to be identical between the structural elements where that is desired, using the `--tied_positions` argument as described[20].

Structure prediction on designed sequences

We primarily use AlphaFold2 [37] to judge if our designed sequences will likely fold or assemble as desired. Models 4 and 5 tend to give the best confidence for these all alpha-helical designed proteins, and often these are the only models used. Designs are judged to be good if they have pLDDT > 90, good pTM score (typically > 0.80) and low CA RMSD (typically < 1.5 Å) to the ideal design model.

Generation of protein assemblies

Curved THR rings and train-track rails are made by producing a monomer length as needed with RepeatPropagationMover, and leaving the outer ends of the proteins with intact repeat sequence so they can use the repeat interactions between chains.

Angle-encoded THRs being used to make cyclic oligomers can theoretically be done easily by hand in PyMOL, but we used the WORMS protocol to find the intended idealized closure solutions. Inputs to worms were split THRs (an THR with a loop region deleted such that it became a 2-chain input) fused to copies of themselves as described in the “Crown” structures in the reference publication [6].

THRs can also be combined with SHDs or other proteins via the WORMs protocol to make similar structures, such as in design “sC4”.

RPXDock can also be used to generate cyclic oligomers without requiring any fusion operations; just by docking THRs against each other, such as in design “hex_C6” (fig. S1G) [8].

For the C2 helical bundle design at the center of the “TT_C2” design, helical placement can be done with the intention that symmetric copies can be generated with appropriate rotation about the Z axis with the SetupForSymmetry mover, as detailed in previous work with these movers [38].

Handshake C2 designs were made in RPXDock by first positioning the THRs such that their repeat axes were parallel to the X axis but offset from it as shown in figure S12. These were then treated as if they were cyclic oligomers already (or an arm extension on an “invisible” cyclic oligomer) and docked into the desired nanocage symmetry that would correspond to the desired THR handshake angle. Designs were selected that maintained the 2 copies of THR at the handshake with their repeat axes parallel to each other (tolerance of approximately +/- 2 degrees) and were then designed with proteinMPNN and evaluated with Alphafold2 as dimers. While any arbitrary angle can be designed in this manner outside the specific polyhedral angles, we did not experimentally investigate them directly.

Arm fusions to designs were done with either: (A) the HelixFuse wrapper for the MergePDBMover, as was done with fuse_2, fuse_3, and fuse_19 (*5*) or (B) RPXDock was used to dock arm to the outside of cyclic oligomer (treating arm as a fake cyclic oligomer as mentioned above, and then using RPXDock Axle sampler [8]; multiple arm position inputs were used for this) and then solutions with chain termini at appropriate positions were looped together with ConnectChainsMover, as was done for the ring fusions in Figure 4 and with the struts added to rings in Figure 3. For the concentric ring designs in Figure 3, the RPXDock Axle sampling was repeated after the first round yielded inner ring + strut so that the outer ring height and rotation could be sampled to dock against the strutted inner pieces.

For the 2 component nanocages shown, RPXDock was used with constraints. Cyclic oligomers with THR arms on them were first pre-oriented such that the arm propagation was aligned to the x-axis. For each symmetry, the x-axis aligned oligomer was additionally rotated to match the symmetry axis of the partner symmetry; for example, in the O43 case, the THR-containing oligomer was rotated +45° around the z-axis. This component was then restricted from rotating during the RPXDock sampling using the `--fixed_rot` option, while the other component was free to sample all normally available DOFs.

For designing interactions between THRs in the case of the train track fibril designs, it did not matter how the tie was docked against the rail so long as the helices remained parallel. For this, samplings of the rigid body positions between the 2 pieces can be obtained by using helix fusion protocols as previously described[6], where the stationary structure is the rail, and the mobile structure is the tie. Outputs of helix fusion then had the fusion helix truncated off of the rail, and the tie was restored to its original length such that the resulting poses feature a tie butting up against the rail, making contacts with one helix of the tie as if it were a continuation of the THR in the rail.

Experimental methods

Construction of synthetic genes

Synthetic genes were ordered from Genscript Inc. (Piscataway, NJ, USA) or Integrated DNA Technologies, Inc. (Coralville, IA, USA) and cloned in pET29b+ *E. coli* expression vector. Most genes feature a 6-Histidine tag for affinity purification. When protein sequences are in the case of a desired N terminal tag, we add that to the sequence and then add a stop codon at the end of the protein sequence so that the vector tag is not expressed. In some cases, such as the 2 component concentric rings, bicistronic expression was used by including a stop and ribosome binding site between 2 protein sequences included in the gene.

Protein production

E. coli expression strain BL21(DE3*) (New England Biolabs, MA, USA) was transformed with plasmid for protein expression. After transformation and overnight growth on LB agar Kanamycin selection plates at either 37°C or 30°C, colonies were picked and transferred to 50mL autoinduction media [39] in 250 mL baffled flasks, where they would typically be incubated at 37°C for 16-24 hours. At the end of this incubation period, cells were harvested by centrifuging at 4000xG for 10 minutes at 4°C.

Protein purification

Cell pellets were resuspended in 30mL lysis buffer on ice; this was performed by vortexing and by mixing with serological pipette. This suspension was lysed by sonication (QSonica Sonicators, CT, USA) at 65% power for 3 minutes (15 sec on/15 sec off) with ¾" tips while keeping the cell suspension on ice. Soluble and insoluble lysates were separated by centrifugation at 4°C and 18,000 G for 40 minutes and applied to chromatography columns containing Ni-NTA (Qiagen, MA, USA) resin pre-equilibrated with lysis buffer. The columns were typically washed twice with 10x column volume of lysis buffer, followed by 15mL of elution buffer for protein elution. Concentration in a 10 kDa molecular weight cutoff spin concentrator was performed after this when necessary.

For two-component nanocages, the two components were expressed in separate cell cultures (50mL each) and harvested into the same tube. The mixed cells were then resuspended in 2.66 mL lysis buffer and sonicated at 80% power with ⅛" tips.

In rare cases, such as with the rails of the train track, the insoluble lysate pellet was taken and resuspended in lysis buffer supplemented with 6M guanidine hydrochloride. This suspension can follow the same centrifugation and Ni affinity purification as above, just with the 6M guanidine hydrochloride added to each buffer, which ultimately was dialyzed away overnight into TBS with Slide-A-Lyzer MINI Dialysis Devices (Thermo Fisher Scientific, MA, USA).

Proteins that expressed well were typically further characterized and purified by size exclusion chromatography (SEC). SEC was done on either the Superdex 6 increase 10/300 GL column (Cytiva, MA, USA) or a Superdex 200 increase 10/300 GL column in TBS buffer or high salt TBS buffer, depending on protein/assembly size. Elution profiles were compared amongst each other in batches of designs and to the manufacturer's provided elution profiles for molecular weight standards.

Small Angle X-ray Scattering (SAXS)

Samples were buffer exchanged to TBS+2% glycerol v/v; a blank buffer was obtained by spin concentrator flow-through. SAXS Scattering measurements were performed at the SIBYLS 12.3.1 beamline at the Advanced Light Source. For each sample, data were collected for two different concentrations to test for concentration-dependent effects; 'low' concentration samples corresponded to ~ 1 mg/ml and 'high' concentration samples to ~ 5 mg/ml. Collected data were processed using the SAXS FrameSlice online server and the FoXS software (Sali Lab) was used to compare experimental scattering profiles to design models and assess quality of fit by computing $\chi 2$ [40]. Profiles were fitted using q values 0.010313 through 0.393757, though plotting only the most reliable data (up to q=0.2).

The FoXS server generates theoretical data from the input design model which it fits to the data using three free parameters, c1 (scaling of atomic radius), c2 (contribution of hydration layer), and an intensity scaling factor. To avoid overfitting, we analyzed the data with c1 and c2 fixed to their default values (1.0 and 0.0, respectively), leaving only the scaling parameter. We also modeled the His-tags with the FloppyTail Rosetta application and used the model with the lowest $\chi 2$ value out of 10 decoys (all decoys' $\chi 2$ values within ~10% of each other) as an additional theoretical model[41]. Generally, the $\chi 2$ values were low (range 1.47 - 0.12), with the models with a tag showing lower $\chi 2$ values (range 0.83 - 0.09) which indicate that these are better models to explain the data.

For the $\chi 2$ calculation, the error is taken from the standard deviation of the raw experimental data which can be affected by buffer subtraction. For these datasets, the $\chi 2$ values for different buffer subtractions are similar to each other so we report the one with the lowest value.

We highlight for comparison the FoXS fit for THR5, a design for which we obtained a crystal structure with 0.6Å C-alpha RMSD to the design model. This model achieved a $\chi 2$ value of 0.33 (0.27 with the tag), which is among the lowest of the dataset suggesting this is still reasonable $\chi 2$ territory. For designs in supplemental figure S1B, these plots are shown in supplemental figure S39.

Buffer recipes

Lysis buffer: 25 mM Tris, 25 mM NaCl, 20 mM Imidazole, pH 8.0 at room temperature

Elution buffer: 25 mM Tris, 25 mM NaCl, 300 mM Imidazole, 50mM EDTA, pH 8.0 at room temperature

TBS buffer: 25 mM Tris pH 8.0, 100 mM NaCl

High salt TBS buffer: 25 mM Tris pH 8.0, 300 mM NaCl

Negative Stain Electron Microscopy

Samples for ns-EM were typically prepared at 0.1 mg/mL concentration for initial screening in a TBS buffer. 5 µL was applied on glow discharged, carbon-coated 400-mesh copper grids (01844-F, TedPella,Inc.), then washed with Milli-Q Water and stained using 0.75% uranyl formate [42]. Air-dried grids were then imaged on a FEI Talos L120C TEM (FEI Thermo Scientific, Hillsboro, OR) equipped with a 4K × 4K Gatan OneView camera at a magnification of 57,000x and pixel size of 2.47 Å. Micrographs collection was automated using EPU software (FEI Thermo Scientific, Hillsboro, OR) and were imported into cryoSPARC software [43]. Typically, micrographs were imported with constant CTF and then manual particle picking of 200-400 particles was done to make templates through 2D classing for automated picking. After full scale templated particle picking, additional 2D classing was done, and selected 2D classes were used for C1 (non-symmetrized) ab initio reconstruction followed by either C1 or symmetric homogeneous refinement, depending on what structural features were being analyzed.

Cryo-EM sample preparation for sC4, sC4_+2, cage_O3_10, cage_T3_5, cage_T3_5_+2, cage_T3_5+6, and tC3_A

Grids (QUANTIFOIL® R 2/2 on Cu 300 mesh grids + 2 nm C) were frozen using a Vitrobot Mark IV with a chamber maintained at 22°C and 100% humidity. 3.5 µl of protein at 0.5 mg/ml (cage_O3_10, cage_T3_5, cage_T3_5+2, cage_T3_5+6), 0.8 mg/ml (sC4, tC3_A), or 1.0 mg/ml (sC4_+2) was applied to the surface of a freshly glow-discharged (for 5 s) grid. Grids were then blotted for 3 - 4 s and plunge-frozen in liquid ethane. Due to low particle density in initial cryo-EM screening, cage_O3_10 was concentrated ~4-fold using 10K MWCO Amicon centrifugal filtration devices prior to grid freezing. All grids were screened at the NYU Cryo-EM core facility using a Talos Arctica microscope operated at 200 kV with a Gatan K3 camera.

Cryo-EM sample preparation for cage_O4_34, cage_O4_34_+4, cage_O43_129, cage_O43_129_+4, cage_O43_129_+8, and cage_T3_101

3 µL of cage_O4_34 (1.0 mg/mL), cage_O4_34_+4 (0.8 mg/mL), cage_O43_129 (0.8 mg/mL), cage_O43_129_+4 (0.8 mg/mL), cage_O43_129_+8 (0.8 mg/mL), or cage_T3_101 (0.8 mg/mL) in 25mM Tris pH 8.0 300mM NaCl was applied to glow-discharged 2/2 Quantifoil carbon grids.

Vitrification was performed on a Mark IV Vitrobot with a wait time of 7.5 seconds, with a blot time of 0.5 seconds, and a blot force of either 0 or -1 before being immediately plunged frozen into liquid ethane. The sample grids were clipped following standard protocols before loaded into the microscope for imaging.

Cryo-EM sample preparation for strut_C6_21

Cryo-EM grids for strut_C6_21 samples were prepared by diluting protein samples with TBS 1 to 10 times immediately before applying 3.5 µL to glow-discharged 400 mesh, C-flat, 2 micron holes, 2 micron spacing, CF-2/2-4C (CF-224C-100) (Electron Microscopy Sciences, Hatfield, PA) cryo-EM grids. Grids were 10 blotted using a blot force of 0 and 5.5 second blot time at 100% humidity and 4°C and plunge-frozen in liquid ethane using a Vitrobot Mark IV (FEI Thermo Scientific, Hillsboro, OR).

Cryo-EM sample preparation for R12B

Four datasets of R12B were collected. For the first two, 3 µL of 10 µM R12B in buffer (300 mM NaCl, 25 mM Tris, pH 8.0) was applied to glow-discharged C-flat 2/2 holey carbon EM grids (Protochips), then blotted and plunge-frozen into liquid ethane using an FEI Vitrobot set to 22 °C with 100% relative humidity. For the third and fourth datasets, 3 µL of 10 µM R12B in buffer + 0.05% fluorinated octyl maltoside was applied to glow-discharged UltrAuFoil 1.2/1.3 holey gold EM grids (Quantifoil), then blotted and plunge-frozen as above.

Cryo-EM data collection, processing and model building of sC4

A total of 3,850 movies were collected with Leginon in super-resolution mode at 0.4124 Å per pixel on a Krios microscope equipped with a K3 camera [44]. Data collection parameters are provided in Supplemental Table S1 and a data processing workflow is provided in Supplemental Figure S25. Movies were pre-processed (2X binned and motion-corrected with MotionCor2) within Appion, then imported to CryoSPARC v.2 and 3 for further processing [43,45,46]. CTF was estimated in CryoSPARC using CTFFIND [47]. 3,781,336 particles were picked using templates generated from processing of a subset of micrographs. Iterative 2D classification was performed in CryoSPARC, and a subset of the 2D-curated particles were used for *ab initio* 3D reconstruction. To curate the particle set in 3D, homogeneous refinement was alternated with C1 heterogeneous refinement with 4 classes- one in which the starting model was the best working reconstruction, and three of which were "junk" classes generated by 3D reconstruction of rejected particles. After the final round of homogeneous refinement in CryoSPARC, the particle set was subjected to a final round of 2D classification after which a few particles were excluded, and 1,212,156 particles were imported to Relion v.3 for a final round of 3D classification [48]. 378,829 particles from the single best class were then refined in Relion using either C4 symmetry or no symmetry (C1). The C4 symmetric map was sharpened using a post-processing job with automatic B-factor assignment. Individual chains of the sC4 design model were docked as rigid bodies into the z-flipped final cryo-EM map using Chimera and imported to Phenix for real-space refinement [49,50]. A single round of simulated annealing was used in the first round of refinement, after which rounds of restrained refinement (using secondary structure, non-

crystallographic symmetry, rotamer, and Ramachandran restraints) were alternated with manual inspection and adjustments in Coot to generate the final model[51]. Map-model correlation coefficients were calculated in Phenix and model geometry was analyzed using MolProbity. Sphericity was estimated using independent half-maps from refinement using the 3DFSC server (https://3dfsc.salk.edu/)[52]. Coordinate refinement details are shown in Supplemental Table S1.

Cryo-EM data collection and processing of cage_O3_10

A total of 4,262 movies were collected with Leginon in super-resolution mode at 0.4124 Å per pixel on a Krios microscope equipped with a K3 camera [44]. Data collection parameters are provided in Supplemental Table S1 and a data processing workflow is provided in Supplemental Figure S26. Movies were imported into CryoSPARC v2 and 3, motion corrected using Patch motion correction and CTF was estimated using the Patch CTF estimation job [43]. After visual curation and removal of 87 micrographs, a subset of micrographs (867) was used to pick particles, both manually and using a blob picker. The reference model for homogeneous refinement used was generated using *ab initio* 3D reconstruction and generated with O symmetry from a subset of 24,040 particles picked from 867 micrographs. After one round of 2D classification using images binned 4 times, the best templates were selected and used as inputs of template picker (663,778 particles picked) and topaz picker (1,336,640 particles picked) to re-pick particles on all of the micrographs. After several rounds of 2D classification and removal of duplicate particles, we obtained 86,379 particles. An initial round of 3D homogenous refinement without symmetry (C1) using all images binned 4 times was performed, using the *ab initio* model as a reference. Following homogeneous refinement, particles were subjected to heterogenous refinement with 3 classes, using 2 decoy references for further sorting of heterogeneity. Particles from 2 of the resulting 3 classes were removed, and the 50,540 particles were then refined using unbinned images. Two rounds of non-uniform refinement in both C1 and O symmetries were performed. Overall resolution was estimated using CryoSPARC implementation of the gold standard method, from which the average reported resolutions are 7.4 and 6.0 respectively. The design model was docked into the final map in Chimera to assess fit of the cryo-EM map to the design model, and analyze the design model-to-map fit [49]. Sphericity was estimated using independent half-maps from refinement using the 3DFSC server (https://3dfsc.salk.edu/).

Cryo-EM data collection and processing of cage_T3_5

A total of 5,854 movies were collected with Leginon in super-resolution mode at 0.4124 Å per pixel on a Krios microscope equipped with a K3 camera [44]. Data collection parameters are provided in Supplemental Table S2 and a data processing workflow is provided in Supplemental Figure S27. Movies were imported into CryoSPARC, motion corrected using Patch motion correction and CTF was estimated using the Patch CTF estimation job [43]. After visual curation and removal of 118 micrographs, a subset of micrographs (1,236) was used to pick particles, both manually and using a blob picker. After one round of 2D classification using images binned 4 times, the best templates were selected and used as inputs of template picker (2,585,392 particles picked) and topaz picker (1,800,346 particles picked) to re-pick particles on all of the micrographs. After several rounds of 2D classifications and removal of duplicate particles, the

resulting 959,145 particles were subject to another round of 2D classification. 676,480 particles from the selected 2D classes were used to generate an *ab initio* model with T symmetry. Homogeneous refinement was performed without symmetry (in C1), using the *ab initio* model as a reference, with 4 times binned images.. Heterogeneous refinement was performed with 3 decoy classes, resulting in 371,057 particles classified into good classes. One round of homogenous refinement and one round of non-uniform homogenous refinement were performed using unbinned images. A final round of heterogeneous refinement was run, resulting in the final particle set. 144,976 particles were refined in C1 symmetry and T symmetry, leading to the final maps, with reported average resolutions of 4.3 Å in C1 symmetry, and 3.6 Å with a T symmetry (class 0 in workflow on Supplemental Figure S27). Class 1 was not an identifiable shape, while classes 2, 3, 4 and 5 appear to be very low resolution reconstructions of the target design, in which part of the intended assembly is missing. Class 3 (116,923 particles) was subjected to another round of heterogeneous refinement leading to two maps, Map 3.0, with a reported average resolution of 6.1 Å, and Map 3.1, with a reported average resolution of 6.5 Å. The design model was docked into the final map in Chimera to assess fit of the cryo-EM map to the design model, and analyze the design model-to-map fit [49]. Map 3.0 clearly shows that the density for one monomer is absent, while Map 3.1 clearly shows that the density for one trimer is absent. Sphericity was estimated using independent half-maps from refinement using the 3DFSC server (https://3dfsc.salk.edu/).

Cryo-EM data collection and processing of cage_T3_5_+2

A total of 19,358 movies were collected with Leginon in 3 sessions, in super-resolution mode at 0.4124 Å per pixel on a Krios microscope equipped with a K3 camera [44]. Data collection parameters are provided in Supplemental Table S2 and a data processing workflow is provided in Supplemental Figure S28. Movies were aligned using Relion v3 's implementation of the motion correction algorithm before being imported into CryoSPARC [43,48]. After patch CTF estimation and curation, a subset of 436 micrographs were used to pick particles, both manually and using blob-picker. After one round of 2D classification with images binned 4 times, the best classes were selected as templates to feed into a template picker and topaz picker jobs to pick particles on all of the micrographs. After removal of duplicates, the resulting 1,318,959 particles were subjected to several rounds of 2D classification. A subset of 363,381 particles was used to generate *ab initio* models with T symmetry. After an initial round of homogeneous refinement without symmetry (C1), further classification was performed using heterogeneous refinement with decoy references to exclude bad particles. The resulting 906,447 particles were refined using homogenous and non-uniform refinement without symmetry (C1). Another round of heterogeneous refinement into 6 classes led to 4 classes which resembled part of the design target, but with parts of the expected target missing. The remaining 2 classes did not resemble any expected shape, and are likely junk particles. The highest resolution map (38% of the particles) was refined without symmetry (C1) leading to a map with reported average resolution of 6.7 Å. The design model was docked into the final map in Chimera to assess fit of the cryo-EM map to the design model, and analyze the design model-to-map fit [49]. Sphericity was estimated using independent half-maps from refinement using the 3DFSC server (https://3dfsc.salk.edu/).

Cryo-EM data collection and processing of sC4_+2, tC3_A, cage_T3_5_+6

All movies from these datasets were collected with Leginon as noted in Supplemental Tables S1, S2, S3 [44], and movies were aligned using Relion's implementation of the motion correction algorithm before being imported into CryoSPARC [43,48]. CTF estimation was performed using the Patch CTF estimation job. Several rounds of 2D classification were performed until a clean stack of particles was obtained.

Cryo-EM data collection and processing of cage_O4_34

Data collection was performed automatically using EPU (FEI Thermo Scientific) to control a ThermoFisher Tundra 100 kV TEM equipped with a standalone CETA-F direct electron detector. Data were collected using fractionation mode, with random defocus ranges spanning between -0.5 and -2.2 μm using image shift and multiple shots per hole. Two sets of movies (3,226 and 1,324) were collected with a pixel size of 1.248 Å.

All data processing was carried out in CryoSPARC [43]. Alignment of movie frames was performed using Patch Motion with an estimated B-factor of 500 Å2, with a maximum alignment resolution set to 5 Å. Defocus and astigmatism values were estimated using Patch CTF with Amplitude Contrast set to 0.07. 252 particles were initially picked using Manual Picker and extracted with a box size of 380 pixels. An initial round of reference-free 2D classification with 10 classes was performed with a maximum alignment resolution of 6 Å, resulting in classes representing views of all three symmetry axes. These classes were used as input for a round of Template Picking with particle diameter of 342 Å, initially picking 248248 particles (before extraction) which were extracted with 380 pix boxes. A round of 2D classification was performed and the particles were sorted into 100 classes. Classes which clearly resolve the cage particles were selected and 59904 particles were used to perform 3D ab initio reconstruction consisting of 3 classes with a maximum alignment resolution of 12 Å using O symmetry. The largest class was refined using Non-uniform Refinement using all particles and using O symmetry with per-particle defocus optimization to arrive at a 7.5 Å map. Viewing Direction Distribution had particle clusters separated by 90 degrees corresponding to slight preferred orientations for the face of the cube. 3D maps for the half maps, final unsharpened maps, and the final maps sharpened by DeepEMhancer [53] (tightTarget model) were deposited in the EMDB under accession number EMD-29915. The processing pipeline for this design is illustrated in Supplement Figure S29, and image processing details are provided in Supplemental Table S4. Figures were generated using UCSF ChimeraX [54].

To validate the use of O-symmetry during reconstruction and refinement, we performed an independent round of Ab-initio reconstruction and Heterogeneous Refinement of 12 classes with C1 symmetry. The 3 classes containing fully resolved cages were subjected to another round of reconstruction Heterogeneous Refinement with all particles, followed by Non-Uniform Refinement to arrive at a 8.7 A map which overlaps well with the O4 map.

Cryo-EM data collection and processing of *cage_O4_34_+4*

Cage_O4_34_+4 cryo-EM grids were screened and data was collected on a ThermoFisher Glacios transmission electron microscope (FEI Thermo Scientific, Hillsboro, OR) operated at 200 kV and equipped with a standalone Gatan K3 Summit direct detector. 3,837 movies were

acquired in counting mode at a nominal magnification of 45,000x (0.89 ˚A/pixel), fractionated in 99 frames at 10.0 e-/$Å^2$/sec for a total dose of 50 e-/$Å^2$ over 5 seconds.

All data processing was carried out in CryoSPARC [43]. Alignment of movie frames was performed using Patch Motion Correction. Defocus and astigmatism values were estimated using Patch CTF with default parameters. A small number of particles were initially picked manually and extracted. 2D classification was used to generate templates for Template Picker, using a 300 Å particle size. The resulting 302,911 locations were extracted with a box size of 706 pixels. This was followed by 2D classification. The best classes, a total of 124,705 particles, were used for 3D ab initio determination using the C1 symmetry operator. This was followed by a Homogeneous Refinement with O symmetry, Local CTF refinement, Global CTF Refinement with exposure groups, and Homogeneous Refinement with O symmetry for a final global resolution estimate of 5.74 Å. 3D maps for the half maps, final unsharpened maps, and the final sharpened maps were deposited in the EMDB under accession number EMD-41907. The processing pipeline for this design is illustrated in Supplemental Figure S32.

Cryo-EM data collection and processing of *cage_T3_101*

Cage_T3_101 cryo-EM grids were screened and data was collected on a ThermoFisher Glacios transmission electron microscope (FEI Thermo Scientific, Hillsboro, OR) operated at 200 kV and equipped with a standalone Gatan K3 Summit direct detector. 3,699 movies were acquired in counting mode at a nominal magnification of 45,000x (0.89 ˚A/pixel), fractionated in 99 frames at 10.0 e-/$Å^2$/sec for a total dose of 50 e-/$Å^2$ over 5 seconds.

All data processing was carried out in CryoSPARC [43]. Alignment of movie frames was performed using Patch Motion Correction. Defocus and astigmatism values were estimated using Patch CTF with default parameters. A small number of particles were initially picked using Blob Picker with a min and max particle diameter of 80 and 300 and extracted. 2D classification was used to generate templates for Template Picker, using a 275 Å particle size. The resulting 1,068,087 locations were extracted with a box size of 490 pixels. This was followed by 2D classification. The best classes, a total of 266,100 particles, were used for 3D ab initio determination using the C1 symmetry operator. This was followed by Non-Uniform Refinement with T symmetry, Local CTF refinement, Global CTF Refinement with exposure groups, and Non-Uniform Refinement with T symmetry for a final global resolution estimate of 5.74 Å. The final map was sharpened using DeepEMhancer[53]. 3D maps for the half maps, final unsharpened maps, and the final sharpened maps were deposited in the EMDB under accession number EMD-41364. The processing pipeline for this design is illustrated in Supplemental Figure S33.

Cryo-EM data collection and processing of *cage_O43_129*

Cage_O4_129 cryo-EM grids were screened and data was collected on a ThermoFisher Glacios transmission electron microscope (FEI Thermo Scientific, Hillsboro, OR) operated at 200 kV and equipped with a standalone Gatan K3 Summit direct detector. 922 movies were acquired in counting mode at a nominal magnification of 45,000x (0.89 ˚A/pixel), fractionated in 99 frames at 10.0 e-/$Å^2$/sec for a total dose of 50 e-/$Å^2$ over 5 seconds.

All data processing was carried out in CryoSPARC [43]. Alignment of movie frames was performed using Patch Motion Correction. Defocus and astigmatism values were estimated using

Patch CTF with default parameters. A small number of particles were initially picked using Blob Picker with a min and max particle diameter of 220 and 260 and extracted. 2D classification was used to generate templates for Template Picker, using a 240 Å particle size. The resulting 32,135 locations were extracted with a box size of 546 pixels. This was followed by 2D classification. The best classes, a total of 13,409 particles, were used for 3D ab initio determination using the O symmetry operator. This was followed by Homogenous Refinement with O symmetry, Reference Motion Correction, and Homogenous Refinement with O symmetry for a final global resolution estimate of 6.77 Å. The final map was sharpened using DeepEMhancer[53]. 3D maps for the half maps, final unsharpened maps, and the final sharpened maps were deposited in the EMDB under accession number EMD-42906. The processing pipeline for this design is illustrated in Supplemental Figure S34.

Cryo-EM data collection and processing of cage_O43_129_+4

Cage_O4_129_+4 cryo-EM grids were screened and data was collected on a ThermoFisher Krios transmission electron microscope (FEI Thermo Scientific, Hillsboro, OR) operated at 300 kV and equipped with a standalone Gatan K3 Summit direct detector. 6,851 movies were acquired in counting mode at a nominal magnification of 105,000x (0.84 ˚A/pixel), fractionated in 100 frames at 10.4 e-/Å^2/sec for a total dose of 52 e-/Å^2 over 5 seconds.

All data processing was carried out in CryoSPARC [43]. Alignment of movie frames was performed using Patch Motion Correction. Defocus and astigmatism values were estimated using Patch CTF with default parameters. A small number of particles were initially picked manually and extracted. 2D classification was used to generate templates for Template Picker. The resulting 23,522 locations were extracted with a box size of 800 pixels. This was followed by 2D classification. The best classes, a total of 13,179 particles, were used for 3D ab initio determination using the C1 symmetry operator. This was followed by Homogenous Refinement with O symmetry, Local CTF refinement, Global CTF Refinement with exposure groups, and Homogenous Refinement with O symmetry for a final global resolution estimate of 6.40 Å. The final map was sharpened using DeepEMhancer[53]. 3D maps for the half maps, final unsharpened maps, and the final sharpened maps were deposited in the EMDB under accession number EMD-42944. The processing pipeline for this design is illustrated in Supplemental Figure S35.

Cryo-EM data collection and processing of *cage_O43_129_+8*

Cage_O43_129_+8 cryo-EM grids were screened and data was collected on a ThermoFisher Glacios transmission electron microscope (FEI Thermo Scientific, Hillsboro, OR) operated at 200 kV and equipped with a standalone Gatan K3 Summit direct detector. 2,990 movies were acquired in counting mode at a nominal magnification of 45,000x (0.89 ˚A/pixel), fractionated in 99 frames at 10.0 e-/Å2/sec for a total dose of 50 e-/Å2 over 5 seconds.

All data processing was carried out in CryoSPARC 43. Alignment of movie frames was performed using Patch Motion Correction. Defocus and astigmatism values were estimated using Patch CTF with default parameters. 8,721 particles were picked manually and extracted with a box size of 900 pixels which were fourier cropped to 380 pixels. This was followed by 2D classification. The best classes, a total of 5,420 particles, were used for 3D ab initio determination using the C1 symmetry operator. This was followed by Non-Uniform Refinement

with O symmetry for a final global resolution estimate of 11.9 Å. 3D maps for the half maps, final unsharpened maps, and the final sharpened maps were deposited in the EMDB under accession number EMD-42031. The processing pipeline for this design is illustrated in Supplemental Figure S36.

Cryo-EM data collection and processing of strut_C6_21

Strut_C6_21 cryo-EM grids were screened and data was collected on a ThermoFisher Glacios transmission electron microscope (FEI Thermo Scientific, Hillsboro, OR) operated at 200 kV and equipped with a standalone Gatan K3 Summit direct detector. Movies were acquired in counting mode at a nominal magnification of 45,000x (0.883 ˚A/pixel), fractionated in 50 frames of 200 ms at 8.5 e-/pixel/sec for a total dose of ~65e-/$Å^2$.

All data processing was carried out in CryoSPARC [43]. Alignment of movie frames was performed using Patch Motion with an estimated B-factor of 500 $Å^2$, with a maximum alignment resolution set to 3. Defocus and astigmatism values were estimated using Patch CTF with default parameters. Strut_C6_21 particles were initially picked in a reference-free manner using Blob Picker and extracted with a box size of 450 pixels. This was followed by multiple rounds of 2D classification and subsequent template-picking using the best 2D class averages. The best classes that revealed clearly visible secondary-structural elements, a total of 37,105 particles, were used for 3D ab initio determination using the C1 symmetry operator. This was followed by a 3D non-uniform refinement with C6 symmetry, global CTF refinement and local refinement for a final global resolution estimate of 5.12 Å. Local resolution estimates were determined in CryoSPARC using an FSC threshold of 0.143. 3D maps for the half maps, final unsharpened maps, and the final sharpened maps were deposited in the EMDB under accession number EMD-29893. The processing pipeline for this design is illustrated in Supplemental Figure S30.

For the model shown in Figure 3C, the design model was fit and refined to the C6 experimental map density using the final model after Phenix refinement with Namdinator [55].

Cryo-EM data collection and processing of R12B

High-throughput data collection was performed with a Gatan K3 Summit direct electron detector on an FEI Glacios Cryo TEM operating at 200 kV accelerating voltage, controlled by SerialEM software [56]. Datasets 2 and 4 were collected with 40° and 30° stage tilt, respectively.

Movies were collected in super-resolution mode, then aligned and corrected for full-frame motion and sample deformation with the patch motion correction algorithm in CryoSPARC v4, with 2x Fourier binning and dose compensation applied during motion correction. All initial processing was performed in CryoSPARC v4 [43]. Contrast transfer function (CTF) was estimated with the patch CTF estimation algorithm, and automatic particle picking using Blob Picker was used to generate template 2D classes for template-based autopicking. Picked particles were boxed and extracted with Fourier cropping and 2D classification was performed iteratively to generate sets of quality particles from all four datasets, which were then combined and run through one more round of 2D classification to select the best-resolved classes. These particles were then re-extracted without Fourier cropping for further processing.

Ab-initio reconstruction was used to generate four volumes and associated classes of particles. The best volume and particle class were used for non-uniform refinement with C12 symmetry to generate an initial map. Local CTF refinement was performed and particles were used for

another round of non-uniform refinement with C12 symmetry. This particle stack was converted to a .star file for processing in RELION 4.0 using the csparc2star.py program in the UCSF pyem collection [57].

Remaining processing was performed in RELION 4.0 [58]. The particle stack imported with csparc2star was reconstructed using relion_reconstruct, and this volume was used as a reference for a 3D auto-refine with no symmetry imposed. Symmetry expansion (relion_particle_symmetry_expand) was used to generate a particle stack expanded with C4 symmetry, which were run through 3D classification without alignment with the sharpened map from 3D auto-refine as a reference. Best-resolved 3D classes were selected, duplicates from symmetry expansion were removed, and another round of 3D auto-refine with no symmetry imposed was performed with the RELION-generated C1 map and a mask generated from the C1 map as references. 3D auto-refine of this new C1 map with C12 symmetry imposed generated a 5.3 Å map. Higher-order aberrations and anisotropic magnification were estimated, and per-particle defocus values and per-micrograph astigmatism were optimized using CTF refinement, which improved the map to a final resolution of 5.2 Å. The processing pipeline for this design is illustrated in Supplementary Figure S31.

Crystallization and Structure Determination

All crystallization experiments were conducted using the sitting drop vapor diffusion method.

Crystallization trials were set up in 200 nL drops using the 96-well plate format at 20 ˚C.

Crystallization plates were set up using a Mosquito LCP from SPT Labtech, then imaged using UVEX microscopes and UVEX PS-256 from JAN Scientific. Diffraction quality crystals formed in 0.2 M Magnesium chloride hexahydrate, 0.1 M BIS-TRIS pH 5.5, and 25% w/v Polyethylene glycol 3,350 for THR1; in 0.3M Sodium nitrate, 0.3M Sodium phosphate dibasic, 0.3M Ammonium sulfate, 0.1M Sodium HEPES; MOPS(acid) pH 7.5, 30% mixture of 40% v/v Ethylene glycol; 20% w/v PEG 8000 for THR2; in 0.2 M Ammonium acetate, 0.1 M HEPES pH 7.5, 25% w/v Polyethylene glycol 3,350 for THR5 and in 0.1M mixture of 0.2M DL-Glutamic acid monohydrate; 0.2M DL-Alanine; 0.2M Glycine; 0.2M DL-Lysine monohydrochloride; 0.2M DL-Serine, 0.1 M mixture of 1M Imidazole; MES monohydrate (acid) pH 6.5, 30% mixture of 40% v/v Ethylene glycol; 20% w/v PEG 8000 for THR6.

Diffraction data was collected at the Advanced Light Source (ALS) 8.2.1/ 8.2.2/ 5.0.2. X-ray intensities and data reduction were evaluated and integrated using XDS and merged/scaled using Pointless/Aimless in the CCP4 program suite [59,60]. Structure determination and refinement starting phases were obtained by molecular replacement using Phaser [61] using the designed model for the structures. Following molecular replacement, the models were improved using phenix.autobuild [62]; efforts were made to reduce model bias by setting rebuild-in-place to false, and using simulated annealing and prime-and-switch phasing. Structures were refined in Phenix [62]. Model building was performed using COOT [63]. The final model was evaluated using MolProbity [64]. Data collection and refinement statistics are recorded in Table S6. Data deposition, atomic coordinates, and structure factors reported in this paper have been deposited in the Protein Data Bank (PDB), http://www.rcsb.org/ with accession code 8G9J (for THR1), 8G9K (for THR2), 8GA7 (for THR5), 8GA6 (for THR6).

Visualization and figures

All structural images for figures were generated using PyMOL (The PyMOL Molecular Graphics System, Version 2.0 Schrödinger, LLC) or ChimeraX [54]. Figures (cartoons and diagrams) were made in InkScape.

Supplementary discussion

Additional polygonal oligomer design details

For the ***Turn*** module-based oligomer designs, a confounding factor for design successes across the different polygonal shapes is that using a hinge helix to produce a sharp turn ends up creating corners with different amounts of local helical interaction density as a function of the turn angle. This gave the n=3 triangles very rigid corners, but the n=5 and n=6 designs were much more hinge-like at the turn helix. We obtained one n=5 design that showed pentagon shapes in ns-EM characterization, although the lower-order n=4 square shape was erroneously prevalent in this design (Supplemental Figure S1F; protein *72_C5_A*). We did not obtain a n=6 hexagon design with this ***Turn*** method, presumably because the hinge region ended up too un-reinforced. So, we made additional hexagon designs that instead were docked to provide increased helical packing density at the corners. We were able to observe 1 hexagon design from an order of 6 that produced the expected hexagonal shape in ns-EM as the dominant species (Supplemental Figure S1G; protein *hex_C6*). Thus, we suggest utilizing this angle-encoding ***Turn*** feature with considerations for local helical density at hinge regions. There are some unique THRs with 3 helices in the repeat unit (Supplemental Figure S5C) which can offer unique looping possibilities for different outcomes after using a ***Turn.***

There are additional cyclic oligomer designs made with Rosetta FastDesign and Rosetta fragment-biased forward folding that are quite different from what we would order today with proteinMPNN and AlphaFold2. Notably, some novel straight-helix heterodimers (SHDs) which were designed to help make cyclic oligomers in Supplemental Figure S1 (proteins *tC3*, *sC4*, and *sC4_+6*) were previously much more prevalent in our building block database (details on construction below). These and also many older THR designs ended up being dropped out of our databases due to new structure prediction capabilities suggesting that they were not nearly as straight as designed. Now, we are much more confident in our capability to create databases of structures that are predicted to be the actual correct shape. The experimental experiences that most closely resemble what a current-day (May 2023) researcher would encounter when designing and testing designs from planned straight helix placement would most likely be similar to “Curve THR rings” (both 18 and 30 repeat experiments) as summarized in Supplemental Figure S2.

We used the SHD blocks to construct closed oligomers by choosing cutpoints on the THRs and the heterodimers that generate the required overall rotation around the Z axis (in contrast to the triangular and square oligomers in Figure 2C, the interfaces between chains have geometries distinct from the interfaces within chains and exhibit more variations in shape) (5). Cryo-EM data for 3 designs yielded 2D class averages in close agreement with the design models (Supplemental Figure S1G, proteins *tC3*, *sC4*, and *sC4_+6*). The top-down views of the particles

show clear densities for the helices, which are very close to the designed placements of helices in the X-Y plane. The distinctive designed interior corner angles for each of these polygon-like structures are evident, 90° for the C4 "square" and 60° for the C3 "hexagon". The C3 "hexagon" has two unique corners that both form similar angles; one at the N terminal fusion of the THR to one half of the SHD, and the other at the C terminal fusion of the THR to the other half of the SHD. The C3 "hexagon" design *tC3* yielded 2D class averages that showed the helices in expected placement (Fig 2E), and the C4 "square" sC4 yielded a 4 Å resolution 3D reconstruction in C4 symmetry with 1.6 Å backbone RMSD to the design model, maintaining the characteristic straightness and phases of all helices (Fig 5A, S9).

Using concentric ring strutting to rescue assembly behavior

When we were planning our designs for ring-strutting (Figure 3) we picked some with imperfections that could hopefully be rescued by reinforcement. For example, we sought to use a correctly sized monodisperse 20-repeat ring to reinforce a 30-repeat ring which had failed to express solubly (fig. S8A). Both rings were cut into ten subunits, the rotation and Z displacement of one ring relative to the other was sampled, and linear THRs were placed to connect the inner and outer rings. The resulting single component C10 strutted assembly formed 10 and 11-mer species in ns-EM 2D classes with the outer ring clearly present, and thus rescued to large extent (Fig. 3A, S8A). In a subset of the classes, a segment of the outer ring was missing, suggesting a distal part of one chain may flay out to alleviate strain. We next used a monodisperse 18-repeat ring to support a 30-repeat ring which on its own formed oblong closed rings often much larger than the design (20-25% increase, fig. S8B). Both were cut into six subunits (with 5 and 3 repeats per subunit, respectively), and the two subunits were connected by a THR strut. The resulting 2 component strutted assembly was monodisperse and close to the design model by ns-EM (Fig. 3B, S8B). Dominant 2D class averages showed both rings with all chains present, without the irregular oblongness that was present in the original 30-repeat ring. A 5.1 Å cryo-EM reconstruction was obtained for this strutted ring sample, which showed that at the regions closest to the strut, the outside diameter was only 2% increased compared to the ideal design model (19.7 nm vs 20.1 nm, Fig. S8C). The helix positioning in the inner ring and the strut were close to the design model (Fig. S8C inset boxes). These results highlight the utility of modular interactions for installing reinforcements that can improve the assembly of nanomaterials.

Additional single-component expandable nanocage designs

If the repeat protein propagation axis is not perfectly aligned in the plane of the symmetry axes (or if the repeat protein is not truly linear), upon extension two outcomes are possible: the building blocks could either flex to compensate, or if the strain in flexing is too large, the assemblies might only partially form. To explore the flexibility of the building blocks, and to investigate whether symmetry could be broken by introduction of strain, we generated designs with varying amounts of repeat propagation axis deviation by docking [8] THRs that were fused to existing cyclic oligomers [6] (fig. S1H, S15) into polyhedral architectures. Of the 12 one-component T3 and O3 nanocages that were tested, cryo electron microscopy maps of a T3 and an O3 design at 4Å and 6Å resolution were very close to the design model, where the helices of the building blocks are clearly visible and aligned as designed. For these designs, based on the

design model, the deviations of the linear THR from the plane spanned by the symmetry axes are approximately 8° (fig. S16, S17). Following addition of one or two repeat units, higher order assembly was still observed, but SEC followed by cryo-EM analysis showed that most of the particles do not contain all expected oligomeric subunits (fig. S17). The *cage_T3_5* expansion series showed clear particles, where *cage_T3_5_+2* and *cage_T3_5_+6* are largely incomplete and are missing a trimer unit when viewed from the 3-fold axis. C1 3D reconstructions showed that a significant population of the particles are missing one trimer. Rigid body fitting of the design model into the *cage_T3_5_+2* reconstruction suggests that the trimer itself is quite rigid, matching well into the map. The three trimer units in turn, to compensate for the increase in length of the THR, "open up" thus preventing the capping fourth trimer from joining the assembly. These results suggest that the angle deviation of the THR propagation axis to the required plane is quite strict, and the flexibility of the well-packed designed protein to compensate for angular deviations is relatively low. This opens an exciting possible design strategy for generating asymmetric protein assemblies with unique subunits to fill the now-empty slot for additional functionality, analogous to the single phage tail-spikes emanating from otherwise symmetric icosahedral phages [65] (fig. S18).

For the C2 "handshake" based designs (Figure 4), variants of the O3, I3, and O4 cages were tested for expandability (no expanded T3 designs were ordered, just because they were deemed less interesting due to being lower order). While the O4 (*cage_O4_34*) worked remarkably well at both the base size and the variant sizes as discussed in main text (Figure 5B), the O3 and I3 cages that were shown in Figure 4 were not as monodisperse in the base size as the O4 cage (Supplemental Figure S13), and upon extension the off-target species became more prevalent. For all of the handshake cages, there is plenty of room left in the sampling space, even with our tight constraints, so it should be very possible to design and filter more C2 handshake angle designs to allow characterization of more designs to select a subset that are similarly robust to *cage_34_O4* to offer more starting places for successful modular expansions. It can be noted that for *cage_O4_34* which had cryo-EM analysis, the interior ring of the tetramer is *R12B* which also had cryo-EM analysis by itself. The ring shows outward size expansion in the context of the cage structure (fig. S14) that is exaggerated compared to what is observed in the "wild-type" originally designed ring structure (fig. S35). There are two main possible reasons for this; first, the position of chain breaks was done at different positions (see supplementary figure S3 for diagrams of these "circular permutations") and, second, there was redesign of some of the ring structure adjacent to the fusion point that may not have packed as designed.

Additional two-component docked nanocage designs for expandability

From a set of two-component docked O43 nanocage designs for the expandability goal, four designs showed monodisperse particles by negative stain EM and yielded 3D reconstructions in octahedral symmetry that show the key features of the assembly including the pores and extended arms (fig. S19-23). For cage_O43_54 and cage_O43_59, parts of the arms formed by

the THR are slightly rotated compared to the design model. Deletion of 4 or addition of 4 or 8 repeat helices from the THRs yielded assemblies in some but not all cases. EM 3D reconstruction of cage_O43_54_-4 and cage_O43_59_+4 yielded maps very similar to the original, where the rotations of both the tetramer and trimer components stay consistent and only the THR portion extends, causing both components to displace further along their respective symmetry axes as expected (fig. S19, S20). To avoid alternative start sites, cage_O43_59 also tolerates mutations to remove methionine residues in the THR (fig. S20). Other sizes of these two cages failed to yield cage-sized assemblies as determined by SEC. An additional cage, cage_O43_164 yielded cages that closely resembled the design model by negative stain EM (fig S23A), but extended variants of the C4 component hosting the THR resulted in insoluble protein. In efforts to solubilize the component, “cut” variants were tested where the first five helices were expressed as a separate protein chain (fig S23B). Cut variants cage_O43_164_cut and cage_O43_164_+4_cut yielded uniform 3-component nanocages when purified via IMAC (fig S23A). The cage_O43_164_cut design matches the parent design as expected, while cage_O43_164_+4_cut matches the expected larger size. The cage_O43_164_+4_cut requires assembly in 2M GuHCl to solubilize the extended component, while additional extension variants remained insoluble.

Comments on assembly yield

Supplemental Figure S37 is included to give examples of typical assembly yield for protein assemblies from THRs. Successful ring designs, both one-component and two-component, typically oligomerize to near completion, with single peaks in the SEC traces (fig S37A). Nanocage designs are more varied; SEC traces for the 4 sizes of *cage_O4_34* are provided to illustrate this. The smallest 2 sizes show most of the protein in single peaks, whereas the largest 2 sizes have additional area under the trace curves for additional partially assembled species, which agrees with the ns-EM characterization. Furthermore, the total protein yield of the larger sizes is lower (fig S37B).

Speculation on thermal and mechanical stability of assemblies from non-globular building blocks

In comparison to structures assembled using globular building blocks, it is possible that using regularized, repetitive building blocks could result in the final architectures having significant thermal and mechanical stability differences. While *de novo* designed proteins are generally very thermostable[18], it has been observed regularly that the terminal helices of repeat proteins are often less rigidly structured than that of the internal repeat units[17]. It is possible that this property is inherited to the higher-order assembly. This is most likely explained by the lack of long-distance contacts (with regard to primary sequence), as the regularized repeat proteins are

defined by repeated local contacts. While the lack of long-distance contacts can contribute to the thermo-flexibility of the protein, we do not observe the long THR building blocks to exhibit excessive flexibility when contributing to cooperativity; structural deviation in the reported nanocages do not stem from the extended THR units deforming (Figure 4, Figure 5, Fig S13, Fig S14, Fig S22). This is consistent with observations of long repeat proteins on the surface of mica[28], and many other nanocages constructed from repeat proteins[66,67].

Similarly, as regularized building blocks are all aligned in the same way in the higher order assembly, this manifests the existence of clear shear planes. This can be observed in some of the larger ring structures where not all of the rings are perfectly round (Figure 2E-F, Fig S1E), but it is not definitive that the alignment is the cause. This can result in potentially mechanically weaker structures, but allow for much more intrinsic designability as modifying local contacts affects much less of the protein building block contacts to the rest of the assembly/protein. As evident in the strutted ring designs, this designability allows for the structure to be reinforced elsewhere.

Supplementary Information References

36. Chaudhury, S., Lyskov, S. & Gray, J. J. PyRosetta: a script-based interface for implementing molecular modeling algorithms using Rosetta. *Bioinformatics* **26**, 689–691 (2010).
37. Jumper, J. *et al.* Highly accurate protein structure prediction with AlphaFold. *Nature* **596**, 583–589 (2021).
38. Boyken, S. E. *et al.* De novo design of protein homo-oligomers with modular hydrogen-bond network-mediated specificity. *Science* **352**, 680–687 (2016).
39. Studier, F. W. Protein production by auto-induction in high density shaking cultures. *Protein Expr. Purif.* **41**, 207–234 (2005).
40. Schneidman-Duhovny, D., Hammel, M. & Sali, A. FoXS: a web server for rapid computation and fitting of SAXS profiles. *Nucleic Acids Res.* **38**, W540–4 (2010).
41. Santiago-Frangos, A., Jeliazkov, J. R., Gray, J. J. & Woodson, S. A. Acidic C-terminal domains autoregulate the RNA chaperone Hfq. *Elife* **6**, (2017).
42. Nannenga, B. L., Iadanza, M. G., Vollmar, B. S. & Gonen, T. Overview of electron crystallography of membrane proteins: crystallization and screening strategies using negative stain electron microscopy. *Curr. Protoc. Protein Sci.* **Chapter 17**, Unit17.15 (2013).
43. Punjani, A., Rubinstein, J. L., Fleet, D. J. & Brubaker, M. A. cryoSPARC: algorithms for rapid unsupervised cryo-EM structure determination. *Nat. Methods* **14**, 290–296 (2017).
44. Suloway, C. *et al.* Automated molecular microscopy: the new Leginon system. *J. Struct. Biol.* **151**, 41–60 (2005).
45. Zheng, S. Q. *et al.* MotionCor2: anisotropic correction of beam-induced motion for improved cryo-electron microscopy. *Nat. Methods* **14**, 331–332 (2017).
46. Lander, G. C. *et al.* Appion: an integrated, database-driven pipeline to facilitate EM image processing. *J. Struct. Biol.* **166**, 95–102 (2009).

47. Rohou, A. & Grigorieff, N. CTFFIND4: Fast and accurate defocus estimation from electron micrographs. *J. Struct. Biol.* **192**, 216–221 (2015).

48. Zivanov, J. *et al.* New tools for automated high-resolution cryo-EM structure determination in RELION-3. *Elife* **7**, e42166 (2018).

49. Pettersen, E. F. *et al.* UCSF Chimera--a visualization system for exploratory research and analysis. *J. Comput. Chem.* **25**, 1605–1612 (2004).

50. Liebschner, D. *et al.* Macromolecular structure determination using X-rays, neutrons and electrons: recent developments in Phenix. *Acta Crystallogr D Struct Biol* **75**, 861–877 (2019).

51. Emsley, P., Lohkamp, B., Scott, W. G. & Cowtan, K. Features and development of Coot. *Acta Crystallogr. D Biol. Crystallogr.* **66**, 486–501 (2010).

52. Tan, Y. Z. *et al.* Addressing preferred specimen orientation in single-particle cryo-EM through tilting. *Nat. Methods* **14**, 793–796 (2017).

53. Sanchez-Garcia, R. *et al.* DeepEMhancer: a deep learning solution for cryo-EM volume post-processing. *Commun Biol* **4**, 874 (2021).

54. Pettersen, E. F. *et al.* UCSF ChimeraX: Structure visualization for researchers, educators, and developers. *Protein Sci.* **30**, 70–82 (2021).

55. Kidmose, R. T. *et al.* Namdinator - automatic molecular dynamics flexible fitting of structural models into cryo-EM and crystallography experimental maps. *IUCrJ* **6**, 526–531 (2019).

56. Mastronarde, D. N. Automated electron microscope tomography using robust prediction of specimen movements. *J. Struct. Biol.* **152**, 36–51 (2005).

57. Asarnow, D., Palovcak, E. & Cheng, Y. *asarnow/pyem: UCSF pyem v0.5*. (2019). doi:10.5281/zenodo.3576630.

58. Kimanius, D., Dong, L., Sharov, G., Nakane, T. & Scheres, S. H. W. New tools for automated cryo-EM single-particle analysis in RELION-4.0. *Biochem. J* **478**, 4169–4185 (2021).

59. Kabsch, W. XDS. *Acta Crystallogr. D Biol. Crystallogr.* **66**, 125–132 (2010).

60. Winn, M. D. *et al.* Overview of the CCP4 suite and current developments. *Acta Crystallogr. D Biol. Crystallogr.* **67**, 235–242 (2011).

61. McCoy, A. J. *et al.* Phaser crystallographic software. *J. Appl. Crystallogr.* **40**, 658–674 (2007).

62. Adams, P. D. *et al.* PHENIX: a comprehensive Python-based system for macromolecular structure solution. *Acta Crystallogr. D Biol. Crystallogr.* **66**, 213–221 (2010).

63. Emsley, P. & Cowtan, K. Coot: model-building tools for molecular graphics. *Acta Crystallogr. D Biol. Crystallogr.* **60**, 2126–2132 (2004).

64. Williams, C. J. *et al.* MolProbity: More and better reference data for improved all-atom structure validation. *Protein Sci.* **27**, 293–315 (2018).

65. Wang, Z. *et al.* Structure of the Marine Siphovirus TW1: Evolution of Capsid-Stabilizing Proteins and Tail Spikes. *Structure* **26**, 238–248.e3 (2018).

66. Yang, E. C. *et al.* Computational design of non-porous, pH-responsive antibody nanoparticles. *bioRxiv* 2023.04.17.537263 (2023) doi:10.1101/2023.04.17.537263.

67. Antanasijevic, A. *et al.* Structural and functional evaluation of de novo-designed, two-component nanoparticle carriers for HIV Env trimer immunogens. *PLoS Pathog.* **16**, e1008665 (2020).

Supplementary figures

Fig. S1. Summary of characterized designs. 37
Fig. S2. Summary of experimental outcomes. 38
Fig. S3. Modular properties of THR ring R12A 39
Fig. S4. Helix phase repetition after 18 residues. 40
Fig. S5. Additional diagrams of THR geometric properties. 41
Fig. S6. Linear THR crystal structures with side chains shown. 42
Fig. S7. THR Ring diagram and raw ns-EM micrographs. 44
Fig. S8. Effects of reinforcement on strutted rings 45
Fig. S9. Images of SHD models. 47
Fig. S10. Cryo-EM experimental model of sC4 compared to design 48
Fig. S11. Geometric constraints for expandable polyhedral symmetrical assemblies. 49
Fig. S12. Design strategy for THR Handshake nanocages 50
Fig. S13. Additional ns-EM data for THR Handshake nanocages 52
Fig. S14. Cryo-EM analysis of a modular O4 nanocage (cage_O4_34). 53
Fig. S15. Examples of HelixFuse de novo helical bundles to THR proteins. 54
Fig. S16. Cryo-EM of cage_O3_10. 55
Fig. S17. Cryo-EM of cage_T3_5 and its expansions. 56
Fig. S18. Cage polarization scheme using cage_T3_5_+2. 57
Fig. S19. Negative stain EM of cage_O43_54 series. 58
Fig. S20. Negative stain EM of cage_O43_59 series. 59
Fig. S21. Negative stain EM of cage_O43_129 series. 60
Fig. S22. Rotational deviation of cage_O43_129 series. 61
Fig. S23. Negative stain EM of cage_O43_164 series. 62
Fig. S24. Additional ns-EM data for train track designs. 64
Fig. S25. cryo-EM data processing pipeline used for sC4. 65
Fig. S26. cryo-EM data processing pipeline used for cage_O3_10. 66
Fig. S27. cryo-EM data processing pipeline used for cage_T3_5. 67
Fig. S28. cryo-EM data processing pipeline used for cage_T3_5_+2. 68
Fig. S29. cryo-EM data processing pipeline used for cage_O4_34. 69
Fig. S30. cryo-EM data processing pipeline used for strut_C6_21. 70
Fig. S31. cryo-EM data processing pipeline used for R12B. 71
Fig. S32. cryo-EM data processing pipeline used for cage_O4_34+4. 72
Fig. S33. cryo-EM data processing pipeline used for cage_T3_101. 73
Fig. S34. cryo-EM data processing pipeline used for cage_O43_129. 74
Fig. S35. cryo-EM data processing pipeline used for cage_O43_129_+4. 75
Fig. S36. cryo-EM data processing pipeline used for cage_O43_129_+8. 76
Fig. S37. Size Exclusion Chromatography (SEC) traces of representative designs 77
Fig. S38. Wider fields of view in ns-EM to show distribution of polygonal design particles 78
Fig. S39. SAXS data plots for tall-helix THR designs made with Rosetta FastDesign 79
Fig. S40. Comparison of helix positions for R12B design model and experimental model from cryo-EM map 80
Fig. S41. Details of cryo-EM model built for cage_T3_101 81
Fig. S42. Details of cryo-EM model built for cage_O43_129 82
Fig. S43. Details of cryo-EM model built for cage_O43_129_+4 83
Table S1. Cryo-EM data collection, processing, and refinement parameters for a subset of designs (I). 85
Table S2. Cryo-EM data collection and processing parameters for a subset of designs (II). 86
Table S3. Cryo-EM data collection and processing parameters for a subset of designs (III). 87
Table S4. Cryo-EM data collection, processing, and refinement parameters for a subset of designs (IV). 89
Table S5. Cryo-EM data collection, processing, and refinement parameters for a subset of designs (V). 91
Table S6. Crystallographic data collection and refinement 92

S1A

Crystal or cryo-EM validated linear THRs

DESIGN NAME	DESIGN IMAGE	FIGURES WITH DESIGN	PROTEIN TYPE	DESIGN METHOD	VALIDATION LEVEL	DATA IMAGE	NOTES OR ADDITIONAL DATA IMAGE	USED IN
THR1	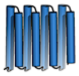	2A, S6	linear, short-helix THR	Rosetta FastDesign and Rosetta fragment-biased forward folding	2.5 Å resolution crystal structure	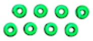	0.8 Å CA RMSD, 2H repeat unit	TT_C2 (see below)
THR2	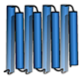	S6	linear, short-helix THR	Rosetta FastDesign and Rosetta fragment-biased forward folding	2.5 Å resolution crystal structure	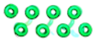	0.8 Å CA RMSD, 2H repeat unit, same backbone as THR1	.
THR3	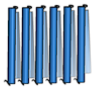	S6	linear, tall-helix THR	Rosetta FastDesign and Rosetta fragment-biased forward folding	3.5 Å resolution crystal structure	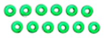	0.4 Å CA RMSD, 2H repeat unit, 8.7 Å helix spacing	.
THR4	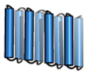	2B, S16,17	linear, short-helix THR, Z-stepping	Rosetta FastDesign and Rosetta fragment-biased forward folding	part of a 4.3 Å resolution cryo-EM nanocage map (T3_96)	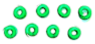	1.0 Å CA RMSD, 2H repeat unit	cage_T3_5 and cage_O3_10 (see below)
THR5	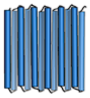	2A, S6	linear, tall-helix THR	Rosetta FastDesign and Rosetta fragment-biased forward folding	2.8 Å resolution crystal structure	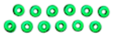	0.6 Å CA RMSD, 3H repeat unit	.
THR6	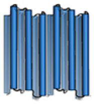	S6	linear, tall-helix THR	Rosetta FastDesign and Rosetta fragment-biased forward folding	2.5 Å resolution crystal structure	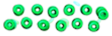	1.3 Å CA RMSD, 3H repeat unit	.

S1B	SAXS validated linear THRs						
**DESIGN NAME**	**DESIGN IMAGE**	**FIGURES WITH DESIGN**	**PROTEIN TYPE**	**DESIGN METHOD**	**VALIDATION LEVEL**	**USED IN**	**USED IN**
THR7	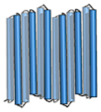	S39	linear, tall-helix THR	Rosetta FastDesign and Rosetta fragment-biased forward folding	SAXS in supp fig S39	.	.
THR8	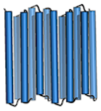	4A, S39	linear, tall-helix THR	Rosetta FastDesign and Rosetta fragment-biased forward folding	SAXS in supp fig S39	sC4 and sC4+2 (see below)	sC4 and sC4+2 (see below)
THR9	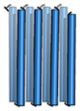	S39	linear, tall-helix THR	Rosetta FastDesign and Rosetta fragment-biased forward folding	SAXS in supp fig S39	.	.
THR10	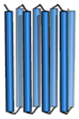	S39	linear, tall-helix THR	Rosetta FastDesign and Rosetta fragment-biased forward folding	SAXS in supp fig S39	.	.
THR11	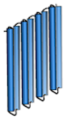	S39	linear, tall-helix THR, Z-stepping	Rosetta FastDesign and Rosetta fragment-biased forward folding	SAXS in supp fig S39	.	.
THR12	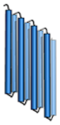	S39	linear, tall-helix THR, Z-stepping	Rosetta FastDesign and Rosetta fragment-biased forward folding	SAXS in supp fig S39	.	.

S1C Linear THRs validated as parts of assemblies

DESIGN NAME	PROTEIN TYPE	DESIGN METHOD	VALIDATION LEVEL	USED IN
THR13	linear THR, Z-stepping	Rosetta FastDesign and Rosetta fragment-biased forward folding	cryoEM classing as part of an oligomer	tC3 see below)
THR14	linear THR	Rosetta FastDesign and Rosetta fragment-biased forward folding	cryoEM classing as part of an oligomer	fuse_19
THR15	linear THR	ProteinMPNN, Alphafold2	nsEM averaging + reconstruction as part of an oligomer	train track rails
THR16	linear THR	ProteinMPNN, Alphafold2	nsEM averaging + reconstruction as part of an oligomer	cage_O3_20
THR17	linear THR	ProteinMPNN, Alphafold2	cryoEM classing and reconstruction as part of an oligomer	cage_O4_34
THR18	linear THR	ProteinMPNN, Alphafold2	nsEM averaging as part of an oligomer	120_C3_A
THR19	linear THR	ProteinMPNN, Alphafold2	nsEM averaging as part of an oligomer	hex_C6
THR20	linear THR	ProteinMPNN, Alphafold2	nsEM averaging as part of an oligomer	90_C4_A
THR21	linear THR	ProteinMPNN, Alphafold2	nsEM averaging as part of an oligomer	120_C3_B, 72_C5_A
THR22	linear THR	ProteinMPNN, Alphafold2	nsEM averaging as part of an oligomer	90_C4_B)

S1D

Unvalidated Linear THRs with high-confidence linear Alphafold 2 predictions

DESIGN NAME	PROTEIN TYPE	DESIGN METHOD	VALIDATION LEVEL	NOTES	NOTES 2
THR23	linear THR	ProteinMPNN, Alphafold2	None as of May 2023	2 helix repeat unit	.
THR24	linear THR	ProteinMPNN, Alphafold2	None as of May 2023	2 helix repeat unit	.
THR25	linear THR	ProteinMPNN, Alphafold2	None as of May 2023	2 helix repeat unit	.
THR26	linear THR	ProteinMPNN, Alphafold2	None as of May 2023	2 helix repeat unit	.
THR27	linear THR	ProteinMPNN, Alphafold2	None as of May 2023	2 helix repeat unit	.
THR28	linear THR	ProteinMPNN, Alphafold2	None as of May 2023	3 helix repeat unit	.
THR29	linear THR	ProteinMPNN, Alphafold2	None as of May 2023	3 helix repeat unit	.
THR30	linear THR	ProteinMPNN, Alphafold2	None as of May 2023	3 helix repeat unit	.
THR31	linear THR	ProteinMPNN, Alphafold2	None as of May 2023	3 helix repeat unit	.
THR32	linear THR	ProteinMPNN, Alphafold2	None as of May 2023	3 helix repeat unit	.
THR33	linear THR	ProteinMPNN, Alphafold2	None as of May 2023	3 helix repeat unit	.
THR34	linear THR	ProteinMPNN, Alphafold2	None as of May 2023	3 helix repeat unit	.
THR35	linear THR	ProteinMPNN, Alphafold2	None as of May 2023	4 helix repeat unit	30° encoded phase change
THR36	linear THR	ProteinMPNN, Alphafold2	None as of May 2023	4 helix repeat unit	30° and 120° phase changes encoded
THR37	linear THR	ProteinMPNN, Alphafold2	None as of May 2023	4 helix repeat unit	30° and 120° phase changes encoded
THR38	linear THR	ProteinMPNN, Alphafold2	None as of May 2023	4 helix repeat unit	30° phase change encoded
THR39	linear THR	ProteinMPNN, Alphafold2	None as of May 2023	4 helix repeat unit	30° and 90° phase changes encoded
THR40	linear THR	ProteinMPNN, Alphafold2	None as of May 2023	4 helix repeat unit	30° and 90° phase changes encoded
THR41	linear THR	ProteinMPNN, Alphafold2	None as of May 2023	4 helix repeat unit	60° phase changes encoded
THR42	linear THR	ProteinMPNN, Alphafold2	None as of May 2023	4 helix repeat unit	60° and 72° phase changes encoded
THR43	linear THR	ProteinMPNN, Alphafold2	None as of May 2023	4 helix repeat unit	60° and 72° phase changes encoded
THR44	linear THR	ProteinMPNN, Alphafold2	None as of May 2023	4 helix repeat unit	90° phase change encoded
THR45	linear THR	ProteinMPNN, Alphafold2	None as of May 2023	4 helix repeat unit	90° phase change encoded
THR46	linear THR	ProteinMPNN, Alphafold2	None as of May 2023	4 helix repeat unit	90° phase change encoded
THR47	linear THR	ProteinMPNN, Alphafold2	None as of May 2023	4 helix repeat unit	120° phase change encoded
THR48	linear THR	ProteinMPNN, Alphafold2	None as of May 2023	4 helix repeat unit	30° phase change encoded
THR49	linear THR	ProteinMPNN, Alphafold2	None as of May 2023	4 helix repeat unit	30° phase change encoded
THR50	linear THR	ProteinMPNN, Alphafold2	None as of May 2023	4 helix repeat unit	60° phase change encoded
THR51	linear THR	ProteinMPNN, Alphafold2	None as of May 2023	4 helix repeat unit	72° phase change encoded
THR52	linear THR	ProteinMPNN, Alphafold2	None as of May 2023	4 helix repeat unit	120° and 72° phase changes encoded
THR53	linear THR	ProteinMPNN, Alphafold2	None as of May 2023	4 helix repeat unit	72° and 90° phase changes encoded
THR54	linear THR	ProteinMPNN, Alphafold2	None as of May 2023	4 helix repeat unit	72° and 90° phase changes encoded
THR55	linear THR	ProteinMPNN, Alphafold34	None as of May 2023	4 helix repeat unit	90° and 30° phase changes encoded

S1E Rings of Curving THRs

DESIGN NAME	DESIGN IMAGE	FIGURES WITH DESIGN	PROTEIN TYPE	DESIGN METHOD	VALIDATION LEVEL	DATA IMAGE	ADDITIONAL DATA OR INFO
R12A	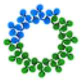	S3	THR ring, 12 repeat, C3 variant	ProteinMPNN, Alphafold2	nsEM class avgs show correct oligomeric state	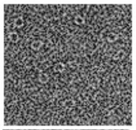	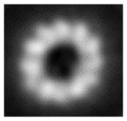
R12B	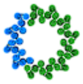	2D, 4	THR ring, 12 repeat, C3 variant	ProteinMPNN, Alphafold2	nsEM (shown) and cryoEM show correct oligomeric state	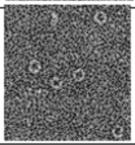	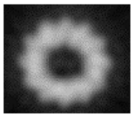
R18A	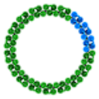	3B, S8B	THR ring, 18 repeat, C6 variant	ProteinMPNN, Alphafold2	nsEM class avgs show correct oligomeric state	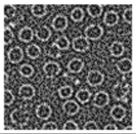	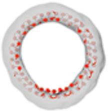
R18B	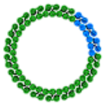	.	THR ring, 18 repeat, C6 variant	ProteinMPNN, Alphafold2	nsEM screening and SEC show similarity to R18A	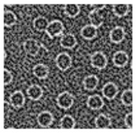	.
R18C	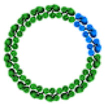	.	THR ring, 18 repeat, C6 variant	ProteinMPNN, Alphafold2	nsEM screening and SEC show similarity to R18A	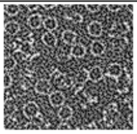	.
R18D	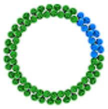	.	THR ring, 18 repeat, C6 variant	ProteinMPNN, Alphafold2	nsEM screening and SEC show larger size than R18A	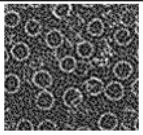	Likely includes 7mer and 8mer ring species preferred over intended 6mer
R18E	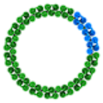	.	THR ring, 18 repeat, C6 variant	ProteinMPNN, Alphafold2	nsEM screening and SEC show similarity to R18A	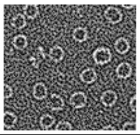	.
R18F	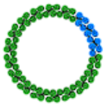	.	THR ring, 18 repeat, C6 variant	ProteinMPNN, Alphafold2	nsEM screening and SEC show similarity to R18A	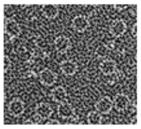	.
R18G	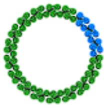	.	THR ring, 18 repeat, C6 variant	ProteinMPNN, Alphafold2	nsEM screening and SEC show similarity to R18A	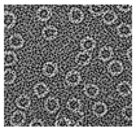	.

R18H	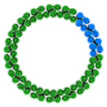	.	THR ring, 18 repeat, C6 variant	ProteinMPNN, Alphafold2	nsEM screening and SEC show similarity to R18A	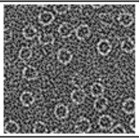	Possibly more broken rings in this design than typical
R18I	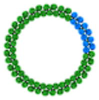	.	THR ring, 18 repeat, C6 variant	ProteinMPNN, Alphafold2	nsEM screening and SEC show similarity to R18A	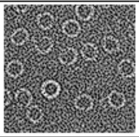	.
R18J	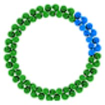	.	THR ring, 18 repeat, C6 variant	ProteinMPNN, Alphafold2	nsEM screening and SEC show larger size than R18A	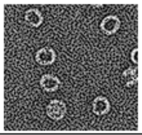	Likely includes 7mer ring species preferred over intended 6mer
R20A	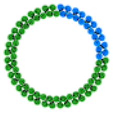	2E, 3A, S8A	THR ring, 20 repeat, C4 variant	ProteinMPNN, Alphafold2	nsEM class avgs show correct oligomeric state	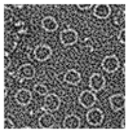	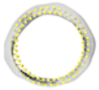
R30A	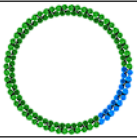	2F	THR ring, 30 repeat, C6 variant	ProteinMPNN, Alphafold2	nsEM class avgs show correct oligomeric state	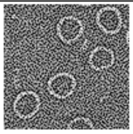	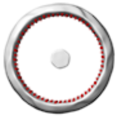
R30B	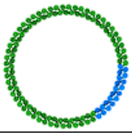	3B, S8B	THR ring, 30 repeat, C6 variant	ProteinMPNN, Alphafold2	nsEM averaging shows 7mer and 6mer species	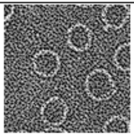	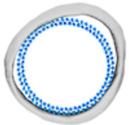
R30C	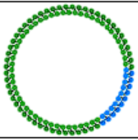	.	THR ring, 30 repeat, C6 variant	ProteinMPNN, Alphafold2	nsEM and SEC show more size polydispersity than R30A	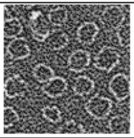	Likely includes 5mer and 7mer ring species in addition to intended 6mer
R30D	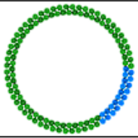	.	THR ring, 30 repeat, C6 variant	ProteinMPNN, Alphafold2	nsEM shows polydispersity and many broken rings	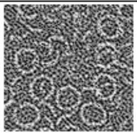	.
R30E	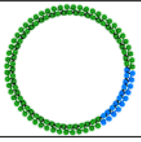	.	THR ring, 30 repeat, C6 variant	ProteinMPNN, Alphafold2	nsEM screening and SEC show similarity to R30A	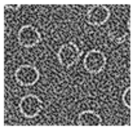	.
R30F	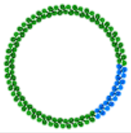	.	THR ring, 30 repeat, C6 variant	ProteinMPNN, Alphafold2	nsEM screening and SEC show smaller size than R30A	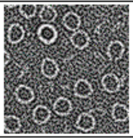	Likely includes 5mer ring species preferred over intended 6mer

S1F Polygon oligomers made with angle-encoded linear THRs

DESIGN NAME	DESIGN IMAGE	FIGURES WITH DESIGN	PROTEIN TYPE	DESIGN METHOD	VALIDATION LEVEL	DATA IMAGE	ADDITIONAL DATA OR INFO
120_C3_A	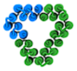	2C	Angle-encoded cyclic oligomer	WORMS with phase-encoded THR, ProteinMPNN, Alphafold2	nsEM class avgs show correct oligomeric state	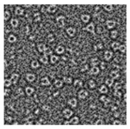	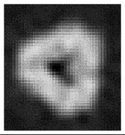
120_C3_B	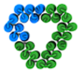	.	Angle-encoded cyclic oligomer	WORMS with phase-encoded THR, ProteinMPNN, Alphafold2	nsEM class avgs show correct oligomeric state	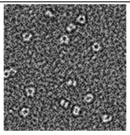	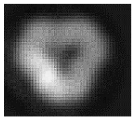
90_C4_A	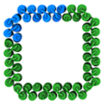	.	Angle-encoded cyclic oligomer	WORMS with phase-encoded THR, ProteinMPNN, Alphafold2	nsEM class avgs show mostly 4mer and some 5mer	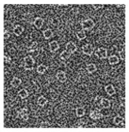	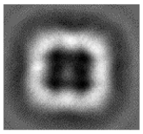
90_C4_B	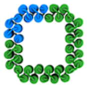	2C	Angle-encoded cyclic oligomer	WORMS with phase-encoded THR, ProteinMPNN, Alphafold2	nsEM class avgs show correct state (poor staining)	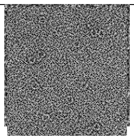	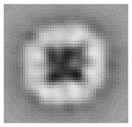
72_C5_A	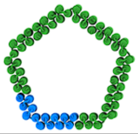	.	Angle-encoded cyclic oligomer	WORMS with phase-encoded THR, ProteinMPNN, Alphafold2	nsEM class avgs show mostly 4mer and some 5mer	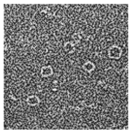	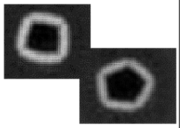

S1G Polygon oligomers made with other methods

DESIGN NAME	DESIGN IMAGE	FIGURES WITH DESIGN	PROTEIN TYPE	DESIGN METHOD	VALIDATION LEVEL	DATA IMAGE	
TT_C2	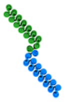	5, S23	C2 oligomer	Parametric bundle helix placement, ProteinMPNN, Alphafold2	nsEM averaging as part of train track fibers	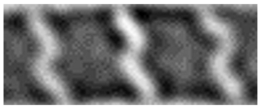	
tC3	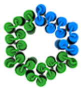	.	C3 oligomer	WORMS of THR13 and SHD heterodimer, Rosetta	cryoEM class averages show correct helix positioning	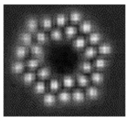	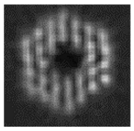
sC4	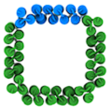	4A	C4 oligomer	WORMS of THR8 and SHD heterodimer, Rosetta	cryoEM reconstruction is correct (averages shown)	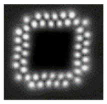	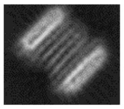
sC4+6	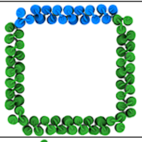	4A	C4 oligomer	Sequence addition to sC4 (2 repeat units)	cryoEM class averages show correct helix positioning	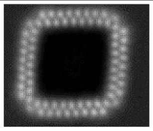	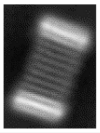
hex_C6	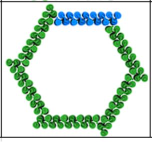	.	C6 oligomer	RPXDock of THR19	nsEM classes show mostly correct 6mer shape and some 7mer	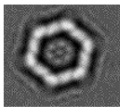	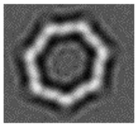

S1H

THR arms added to previously studied oligomer

DESIGN NAME	DESIGN IMAGE	FIGURES WITH DESIGN	PROTEIN TYPE	DESIGN METHOD	VALIDATION LEVEL	DATA IMAGE	ADDITIONAL DATA OR INFO
fuse_2	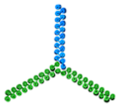	.	Fusion of THR4 to 5L6_HC3 (38)	Hfuse, Rosetta	nsEM class averages show correct oligomeric state	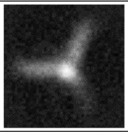	This does not give definitive evidence of THR straightness
fuse_3	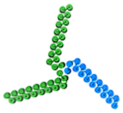	S15	Fusion of THR4 to 5L6_HC3 (38)	Hfuse, Rosetta	nsEM class averages show correct oligomeric state	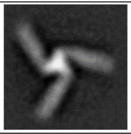	Characterized further in T3_5 and O3_10 nanocages
fuse_19	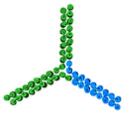	.	Fusion of THR15 to 5L6_HC3 (38)	Hfuse, Rosetta	nsEM class averages show correct oligomeric state	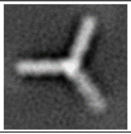	This does not give definitive evidence of THR straightness

S1I

THR C2 Handshake designs

DESIGN NAME	DESIGN IMAGE	FIGURES WITH DESIGN	PROTEIN TYPE	DESIGN METHOD	VALIDATION LEVEL	ADDITIONAL DATA OR INFO
T3_C2	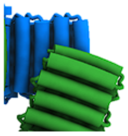	4B	THR Handshake C2	Constrained RPXDock, ProteinMPNN, Alphafold2	nsEM class averages and reconstruction from nanocage	see cage_T3_101 and cage_T3_104 below
O4_C2_A	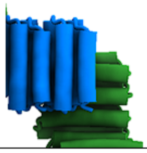	4E, 5B	THR Handshake C2	Constrained RPXDock, ProteinMPNN, Alphafold2	nsEM and cryoEM nanocage reconstructions	see cage_O4_34 below
O3_C2	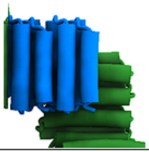	4C	THR Handshake C2	Constrained RPXDock, ProteinMPNN, Alphafold2	nsEM class averages and reconstruction from nanocage	see cage_O3_20 below
I3_C2	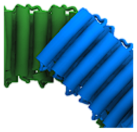	4D	THR Handshake C2	Constrained RPXDock, ProteinMPNN, Alphafold2	nsEM class averages and reconstruction from nanocage	see cage_I3_8 below

S1J

THR nanocage designs

DESIGN NAME	DESIGN IMAGE	FIGURES WITH DESIGN	PROTEIN TYPE	DESIGN METHOD	VALIDATION LEVEL	DATA IMAGE
cage_T3_101	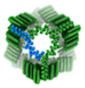	4B, S41	tetrahedral T3 nanocage	Planned combination of armed ring and handshake C2	cryoEM reconstruction	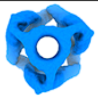
cage_T3_104	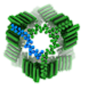	.	tetrahedral T3 nanocage	Planned combination of armed ring and handshake C2	nsEM reconstruction	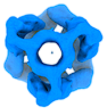
cage_T3_5	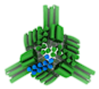	S17	tetrahedral T3 nanocage	RPXDock of fuse_3, Rosetta	cryoEM reconstrction	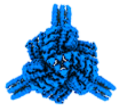
cage_T3_5+2	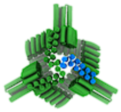	S17	tetrahedral T3 nanocage	RPXDock of fuse_3, Rosetta	cryoEM reconstrction	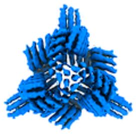
cage_O4_32	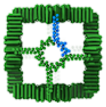	.	octahedral O4 nanocage	Planned combination of armed ring and handshake C2	nsEM class averaging	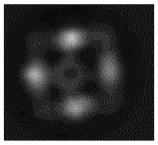
cage_O4_33	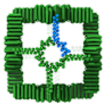	.	octahedral O4 nanocage	Planned combination of armed ring and handshake C2	nsEM class averaging (wrong dominant species)	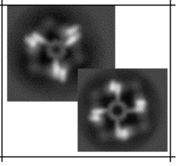

cage_O4_34	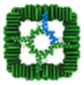	4E, 5B, S14	octahedral O4 nanocage	Planned combination of armed ring and handshake C2	cryoEM reconstruction	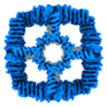
cage_O4_34+4	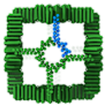	5B	octahedral O4 nanocage	Planned combination of armed ring and handshake C2	cryoEM reconstruction	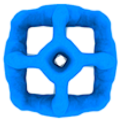
cage_O4_34+8	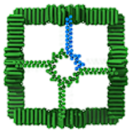	5B	octahedral O4 nanocage	Planned combination of armed ring and handshake C2	nsEM reconstruction	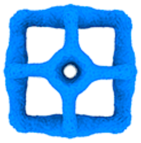
cage_O4_34+12	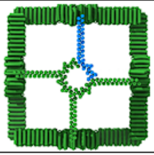	5B	octahedral O4 nanocage	Planned combination of armed ring and handshake C2	nsEM reconstruction	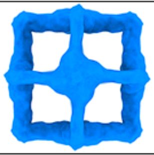
cage_03_10	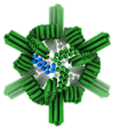	S16	octahedral O3 nanocage	RPXDock of fuse_3, Rosetta	cryoEM reconstruction	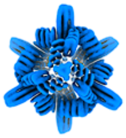
cage_O3_20	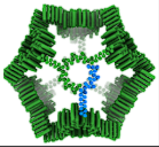	4C	octahedral O3 nanocage	Planned combination of armed ring and handshake C2	nsEM reconstruction, the majority species is smaller	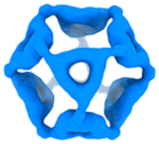
cage_I3_8	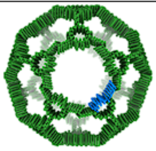	4D	icosahedral I3 nanocage	Planned combination of armed ring and handshake C2	nsEM reconstruction, unclear if most particles are missing subunits or not	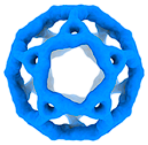
cage_O43_54	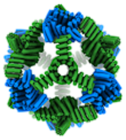	S18	octahedral O43 2-component nanocage	constrained RPXDock, proteinMPNN	nsEM reconstruction	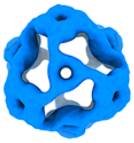

cage_O43_54-4	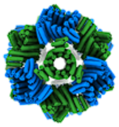	S19	octahedral O43 2-component nanocage	constrained RPXDock, proteinMPNN	nsEM reconstruction	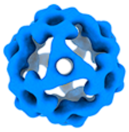
cage_O43_59	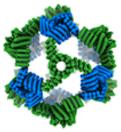	S20	octahedral O43 2-component nanocage	constrained RPXDock, proteinMPNN	nsEM reconstruction	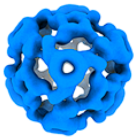
cage_O43_59+4	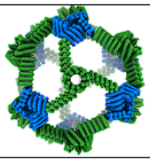	S20	octahedral O43 2-component nanocage	constrained RPXDock, proteinMPNN	nsEM reconstruction	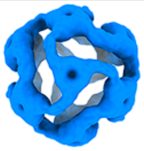
cage_O43_164	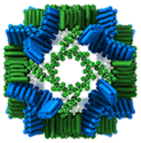	S23	octahedral O43 2-component nanocage	constrained RPXDock, proteinMPNN	nsEM reconstruction	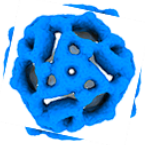
cage_O43_129	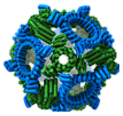	5D,S21, S22, S42	octahedral O43 2-component nanocage	constrained RPXDock, proteinMPNN	cryoEM reconstruction	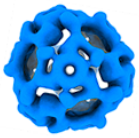
cage_O43_129+4	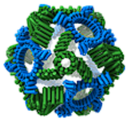	5D,S21, S22, S43	octahedral O43 2-component nanocage	constrained RPXDock, proteinMPNN	cryoEM reconstruction	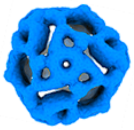
cage_O43_129+8	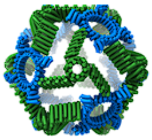	5D, S22	octahedral O43 2-component nanocage	constrained RPXDock, proteinMPNN	cryoEM reconstruction	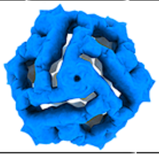
cage_O43_129+12	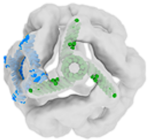	5D, S22	octahedral O43 2-component nanocage	constrained RPXDock, proteinMPNN	nsEM reconstruction	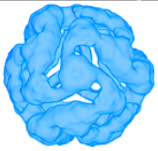

S1K	THR strutted designs					
**DESIGN NAME**	**DESIGN IMAGE**	**FIGURES WITH DESIGN**	**PROTEIN TYPE**	**DESIGN METHOD**	**VALIDATION LEVEL**	**DATA IMAGE**
strut_C6_21	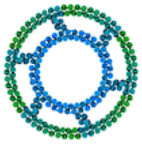	3B, S8	2 component C6 concentric rings	RPXDock, proteinMPNN, Alphafold2	cryoEM reconstruction	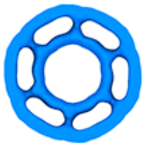
strut_C6_16	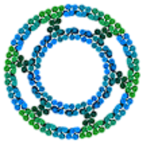	.	2 component C6 concentric rings	WORMS, proteinMPNN, Alphafold2	nsEM reconstruction, some rings are squished in nsEM	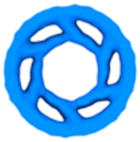
strut_C10_8	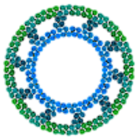	3A, S8	1 component C10 concentric rings	WORMS, proteinMPNN, Alphafold2	nsEM reconstruction	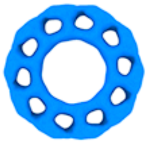
TT_base	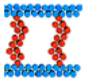	6, S24	2 component unbound fiber	Helix overlap, proteinMPNN, Alphafold2	nsEM reconstruction	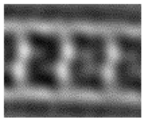
TT_rail+	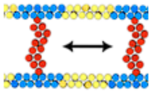	6, S24	2 component unbound fiber	Helix overlap, proteinMPNN, Alphafold2	nsEM reconstruction	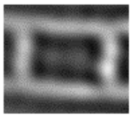
TT_tie+	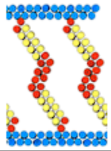	6, S24	2 component unbound fiber	Helix overlap, proteinMPNN, Alphafold2	nsEM reconstruction	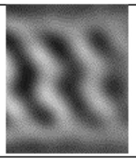
TT_rail+_tie+	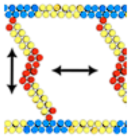	6, S24	2 component unbound fiber	Helix overlap, proteinMPNN, Alphafold2	nsEM reconstruction	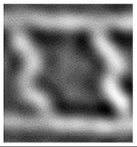

S1L

Summary of designs included in this work			
**Figure Section**	**Design type**	**Number of entries**	**Level of characterization**
S1A	Linear THRs	6	crystal structure or cryo-EM structure
S1B	Linear THRs	6	SAXS analysis
S1C	Linear THRs	10	ns-EM and cryo-EM structure validation when used as part of an oligomer
S1D	Linear THRs	33	design models
S1E	Curving THRs tested as rings	19	ns-EM structure validation
S1F	Polygon oligomers from Turn module	5	ns-EM 2D class averages
S1G	Other cyclic oligomers	5	cryo-EM and ns-EM structure validation
S1H	THR arm fusions to published oligomer	3	ns-EM 2D class averages
S1I	C2 Handshake designs	4	cryo-EM and ns-EM as part of nanocages
S1J	Nanocage designs	22	cryo-EM and ns-EM structure validation
S1K	Strutted THR designs	7	cryo-EM and ns-EM structure validation
	Total (with some redundancy)	120	

Fig. S1. Summary of characterized designs.

This is a reference table for all designs that are included with this publication. (**A**) Summary data for linear THRs that had either crystal structure or were solved as part of a cryo-EM structure. (**B**) Summary data for linear THRs that gave reasonable SAXS profiles. (**C**) Summary data for THRs that were validated as parts of other protein assemblies without a fully solved structure. (**D**) List of THRs that achieved high quality AlphaFold2 structure predictions, but haven't been validated yet. (**E**) Summary data for rings made from curved THRs. (**F**) Summary data for polygon oligomers made from angle-encoded THRs. (**G**) Summary data for cyclic oligomers made with other methods and THRs. (**H**) Summary data for THR arms added to a previously characterized design. (**I**) Handshake C2 designs that are pulled out of working nanocage designs. (**J**) Summary data for nanocages made with THR building blocks.(**K**) Summary data for strutted THR designs. (**L**) Table with counts of each design type from this collection.

Experiment	# soluble by SDS-PAGE	# correct size by SEC	further characterization
1st generation Rosetta Linear THRs	23/33	13/19	6 SAXS, 5 crystal, 2 cryo-EM as assembly
Curve THRs (18 repeat ring)	33/48	16/33	8 nsEM rings correct size, 2 of larger size
Curve THRs (30 repeat ring)	25/48	7/25	2 nsEM rings correct size, 5 with polydispersity
Angle-encoded THR cyclic oligomer (C3-C6)	10/20	7/10	4 nsEM correct, 1 nsEM as wrong state
Handshake nanocages feat. R12B THR ring	13/13	10/13	7 nsEM show the designed species
Handshake nanocages with other C3 hub	15/26	0/15	NA
Rosetta Straight helix heterodimer pairs (SHDs)	32/34	13/34 *	1 verified in cryo-EM as part of assembly
Constrained RPXDock O43**	16/24	2/24	1 was shown correct by nsEM reconstruction

Fig. S2. Summary of experimental outcomes.

Summary of representative final rounds of design strategies for different architectures tested experimentally, showing a general sense of "success rate". Number of designs yielding soluble protein via SDS-PAGE represent proteins post immobilized metal affinity chromatography (IMAC). Soluble designs were then passed through size exclusion chromatography (SEC). Designs that show elution peaks in the right size by SEC were then subject to appropriate biochemical characterization such as electron microscopy. (*:characterization was instead performed by Native-PAGE where appearance of a novel band upon combination of components suggested their interaction, **: this is from the last round of testing this architecture; there were additional successes from previous rounds)

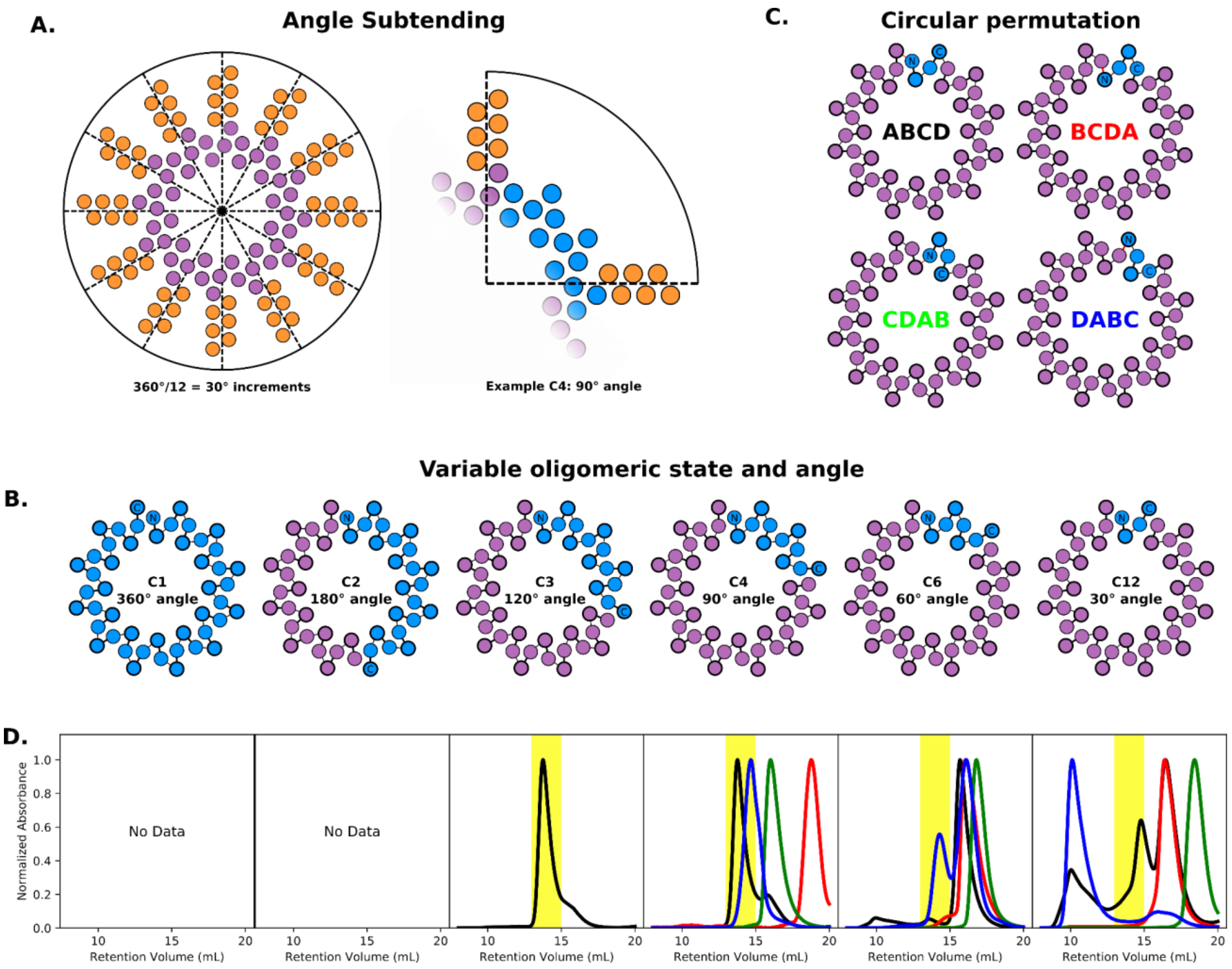

Fig. S3. Modular properties of THR ring R12A

(A) A internally C12 curved THR ring structure can be subdivided in 30° increments; for example, a C4 variant of the ring would yield a 90° angle. (B) Depending on desired angle, the protein can be spliced into a variety of cyclic oligomers. (C) The structure is also planned in a way that circular permutations are simple, thus depending where fusion positions are needed, all eight (N- and C-) helical termini are available. (D) Preliminary experimental validation (size exclusion chromatography) confirms that simple splicing can produce proteins of the correct oligomeric state (yellow highlight), although some splices and circular permutations (color of the curve matches the color of the text from C) yield unassembled building blocks. C1 and C2 variants were not experimentally tested.

Residue position	Degrees of rotation	Cumulative turns	Phase sampled
0	0	0.0	0
1	100	0.3	100
2	200	0.6	200
3	300	0.8	300
4	400	1.1	40
5	500	1.4	140
6	600	1.7	240
7	700	1.9	340
8	800	2.2	80
9	900	2.5	180
10	1000	2.8	280
11	1100	3.1	20
12	1200	3.3	120
13	1300	3.6	220
14	1400	3.9	320
15	1500	4.2	60
16	1600	4.4	160
17	1700	4.7	260
18	1800	5.0	360

Fig. S4. Helix phase repetition after 18 residues.
The "alpha_helix_100" helices turn 100 degrees with every increment in sequence/residue position. After 1800 degrees, there have been exactly 5 complete turns (5 multiples of 360°). In the "Phase sampled" column, we see that the "Degrees of rotation" actually samples all the 20° increments in 0-360° if the "Degrees of rotation" are considered for their net rotation in a 0-360° range (if rotation exceeds 360°, subtract integer multiples of 360° until the rotation is in the 0-360° range).

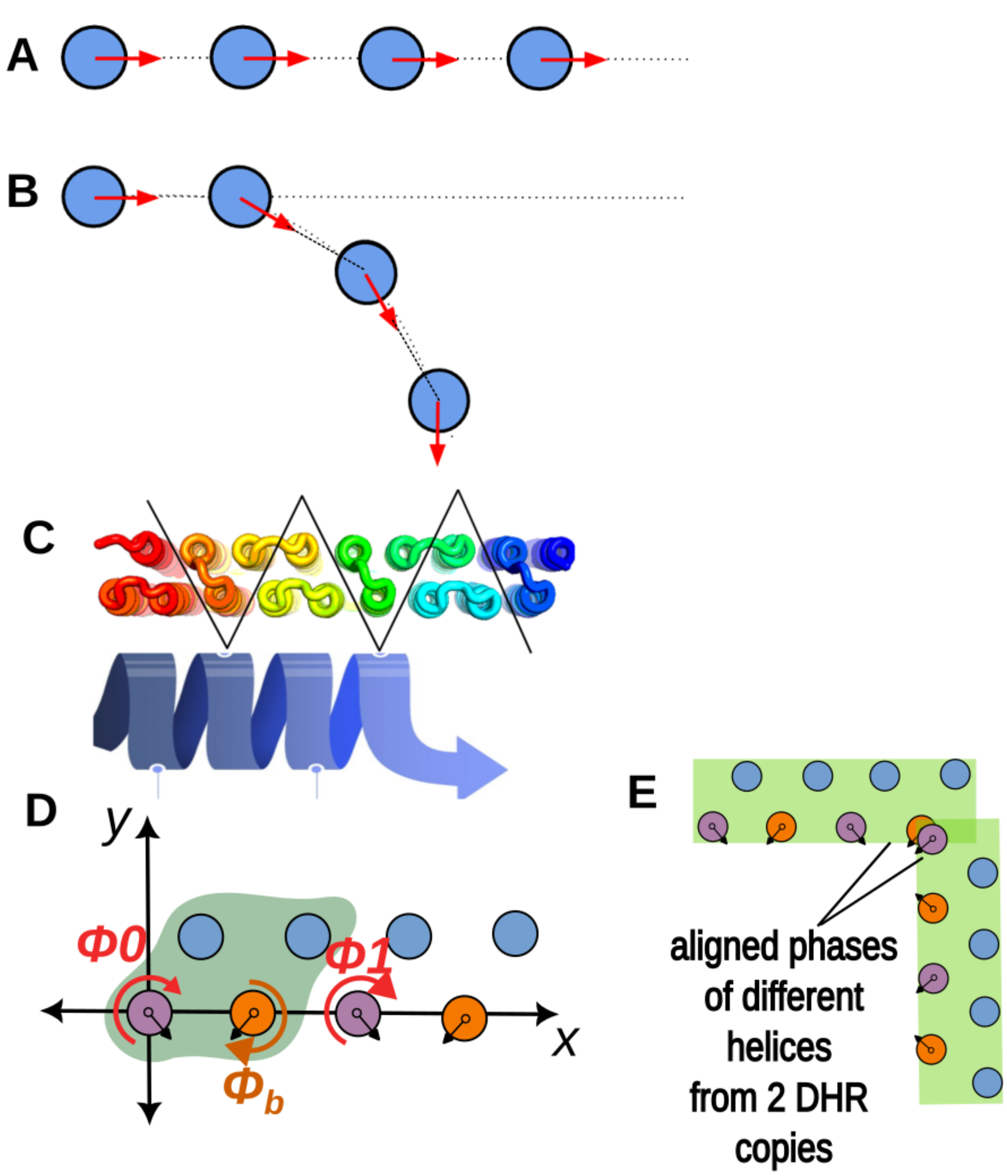

Fig. S5. Additional diagrams of THR geometric properties.

(A) Here we see that a THR repeat trajectory can be linear if there is no change in helix phase between the first and second copies of the “a” helix. (B) Here we see that a THR repeat trajectory will curve if there is a change in helix phase relative to the dashed line between the centers of the first and second copies of the “a” helix. (C) THRs with a repeat unit of 3 helices can be designed to be perfectly linear (such as THR5 and THR6) if they follow this flipping scheme when the helices are placed. (D) In a 4 helix repeat unit, the orange helix can be lined up with the trajectory-setting helices, but its phase can be freely sampled; this means if a purple helix were fused onto an orange helix, the fusion could make any assigned angle between linear THRs, as illustrated in (D).

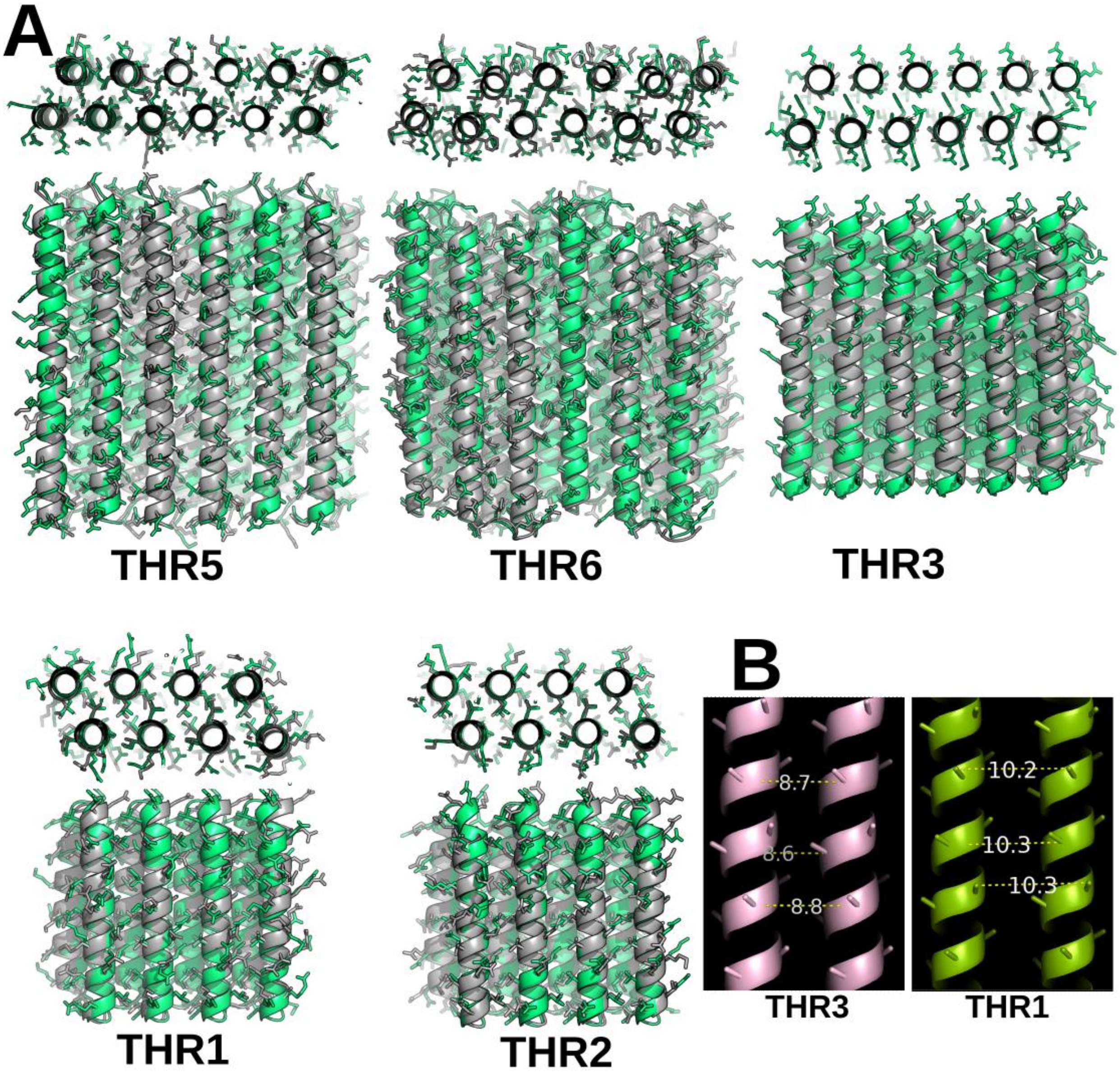

Fig. S6. Linear THR crystal structures with side chains shown.

(A) Grey crystal structures of linear THRs overlaid with design models in green, with side chains shown on both. THR3 only had enough resolution to show CA-CB atoms of the side chain sticks in the crystal structure model. (B) Measurements from PyMOL taken on representative innermost residue positions of THR crystal structures at matched positions in different repeat units (THR3 design spacing = 8.8 Å, THR1 design spacing = 10.0 Å)

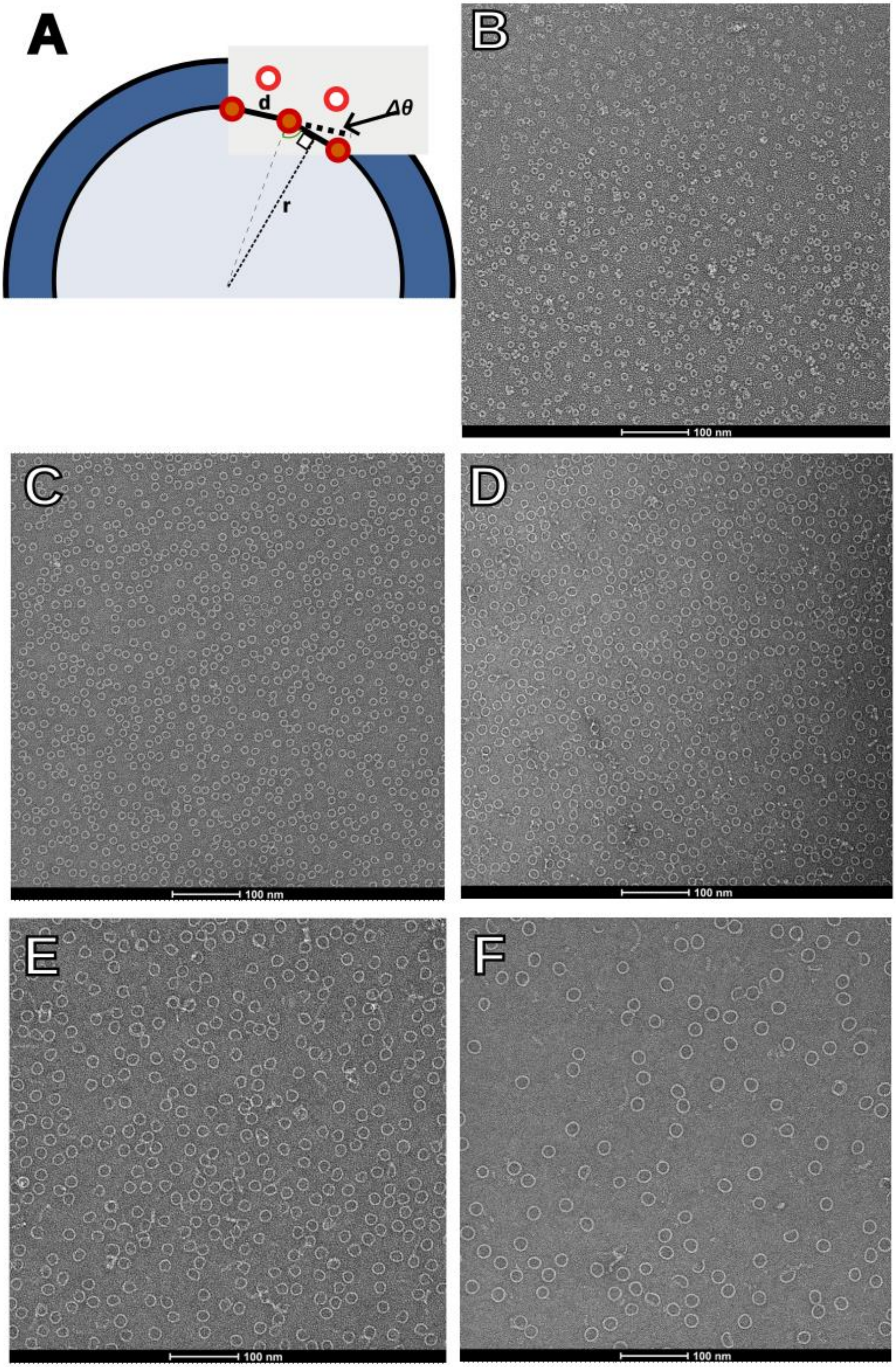
A
d
Δθ
r
B
100 nm
C
100 nm
D
100 nm
E
100 nm
F
100 nm

Fig. S7. THR Ring diagram and raw ns-EM micrographs.

(A) This shows how a diameter (of a circle containing the centers of repeat-setting “a” helices) can be described as a function of the repeat parameters ***d*** and ***Δθ***: $radius = \frac{d}{2} \times tan\left(\frac{180-|\Delta\theta|}{2}\right)$; this value is smaller than the outer diameter dimension. (B-F) Raw ns-EM micrographs of ***Curve*** THR rings. (B) *R12A* ring with 12 repeats, tested as C4 with expected outer diameter of 9 nm. (C) *R18G* ring with 18 repeats, tested as C6 with expected outer diameter of 12 nm. (D) *R20A* ring with 20 repeats, tested as C4 with expected outer diameter of 14 nm. (E) *R30E* ring with 30 repeats, tested as C6 with expected outer diameter of 21 nm. (F) *R30A* ring with 30 repeats, tested as C6 with expected outer diameter of 22 nm.

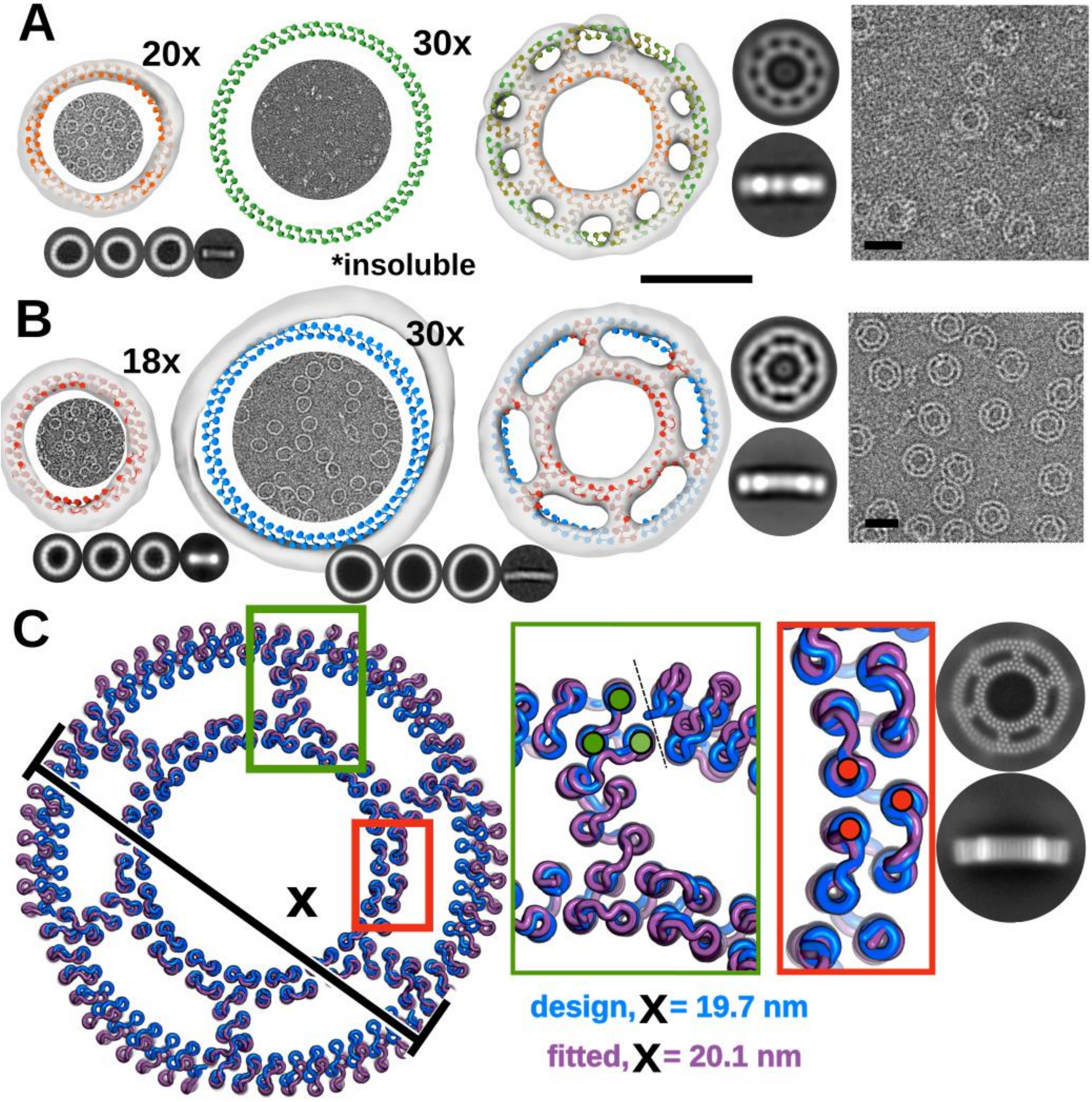

Fig. S8. Effects of reinforcement on strutted rings

(A-B) Single ring (first two columns) and double ring (3rd column) design models are shown in cylindrical helix representation and overlaid with C1 ns-EM reconstructions. The deviations from perfect circular shape are much larger for the single rings than the reinforced double ring structures. ns-EM micrograph samples of the single rings are shown inside of the design models and of the double rings, at the far right. Black scale bars = 10 nm, 2D class averages are not to scale. (A) (Left) A 20-repeat ring *R20A* (orange), tested as a C4 was monodisperse and close to

the intended size. (Middle) A 30-repeat ring (green) tested as a C6 expressed insolubly. (Right) A strutted combination of these rings as a single component C10 design *strut_C10_8* rescues the outer ring, as illustrated in the reconstruction, the 2D class averages, and in the raw micrograph. (B) (Left) An 18-repeat ring *R18A* (red), tested as a C6, was monodisperse and close to the intended size. (Middle) a 30-repeat ring *R30B* (blue) tested as an intended C6, was seen more often as a heptamer than a hexamer and was often oblong. (Right) A strutted combination of these rings as a two-component C6 design *strut_C6_21* with the struts attached to the inner ring. (C) Design model of the two-component C6 design *strut_C6_21* from B (blue) with cryo-EM fitted model (purple) shows good alignment in the inner ring (red inset) while deviation is observed in the interface (dashed line) between strut and outer ring (green inset). Structures in inset boxes are locally aligned to 3 helices, indicated by filled circles on top of the helices.

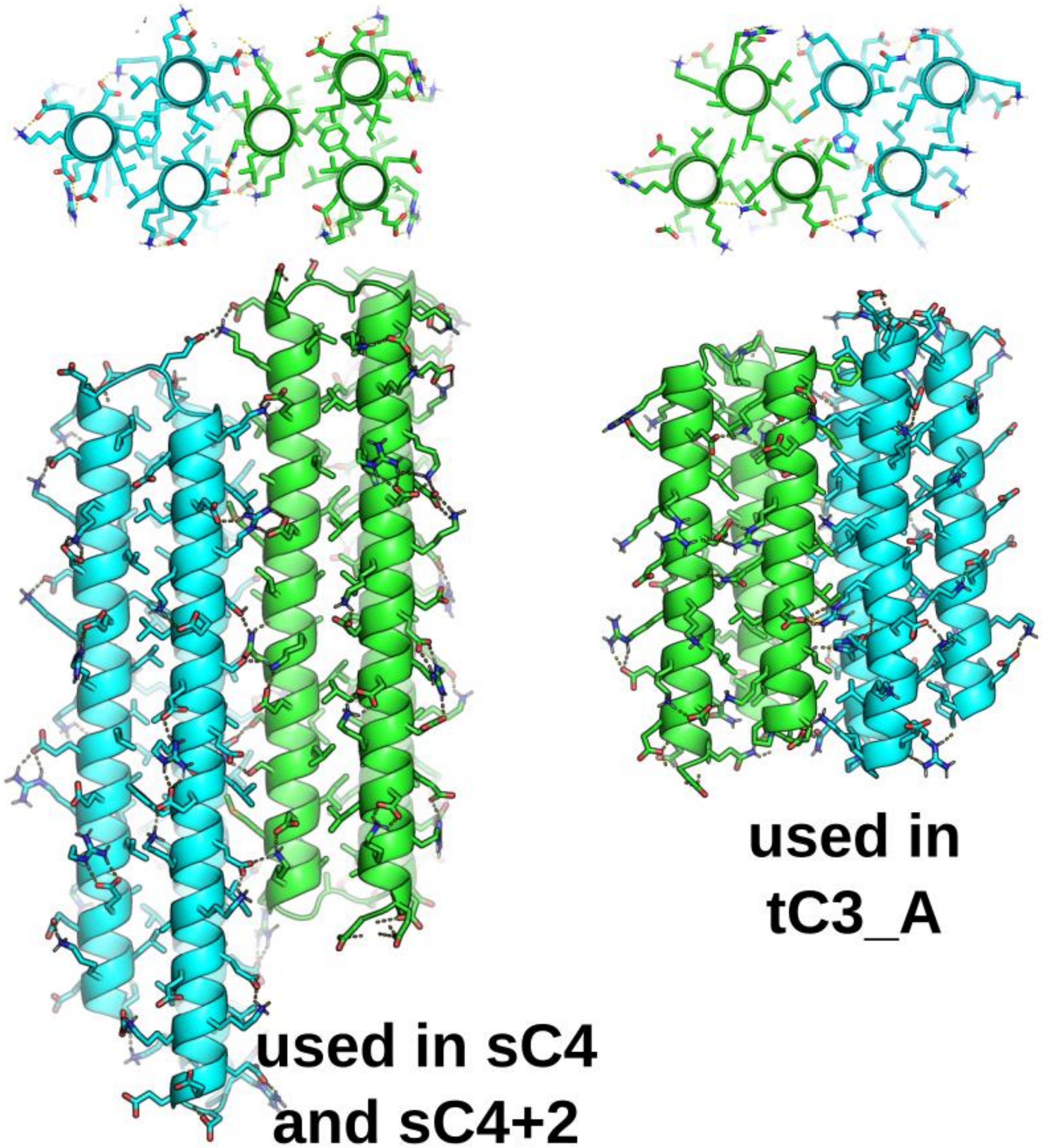

Fig. S9. Images of SHD models.

Examples of design models of straight hetero-dimers (SHDs) utilizing buried hydrogen bond networks to confer interaction specificity.

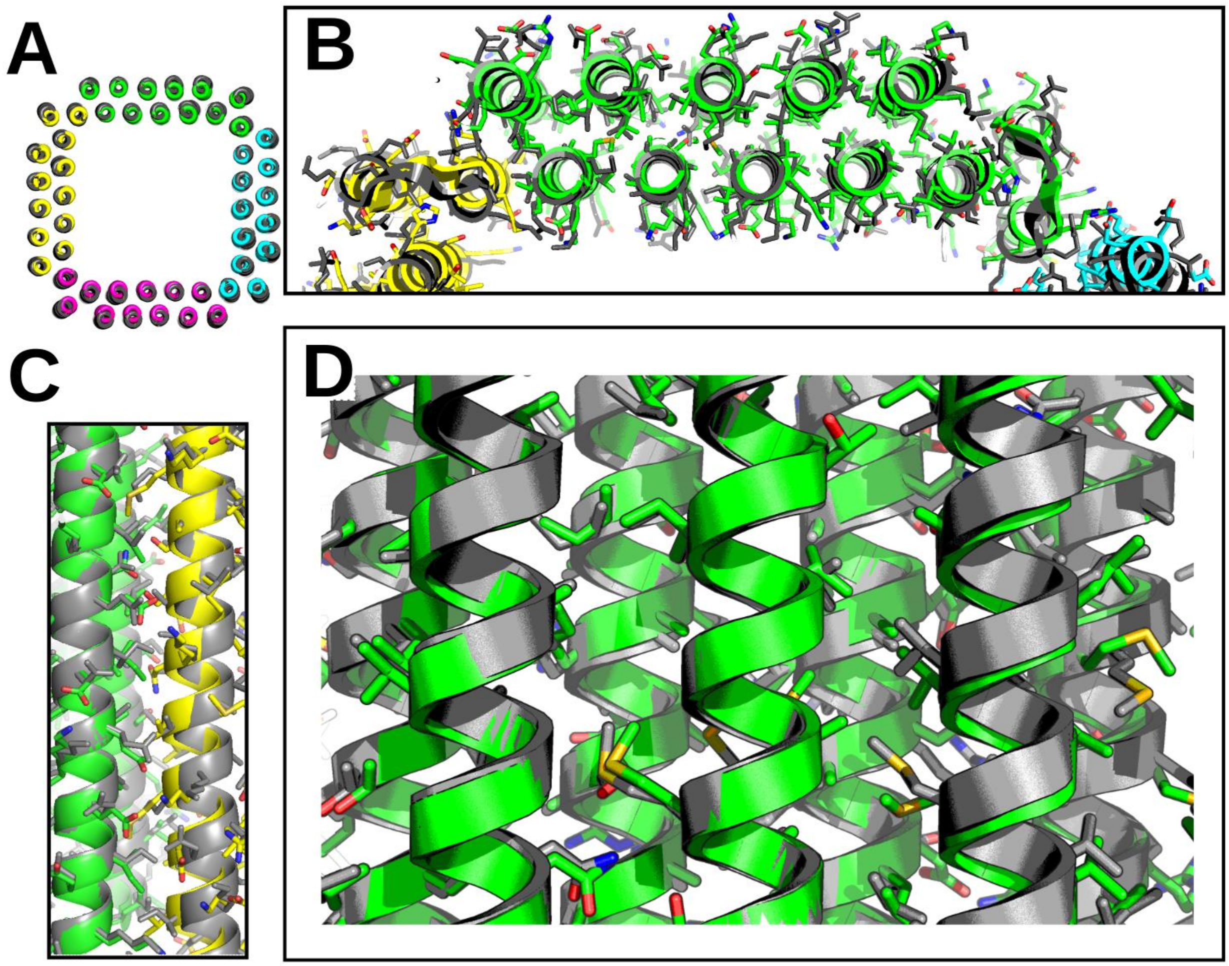

Fig. S10. Cryo-EM experimental model of sC4 compared to design

Design model (colored) overlaid with cryo-EM structure (gray). (A) View from down the z-axis (1.6 Å backbone RMSD). Zoom in on the top (B) and side (C) views showing overall agreement of helices. (D) Zoomed in view showing overall agreement of hydrophobic side chain residues.

A

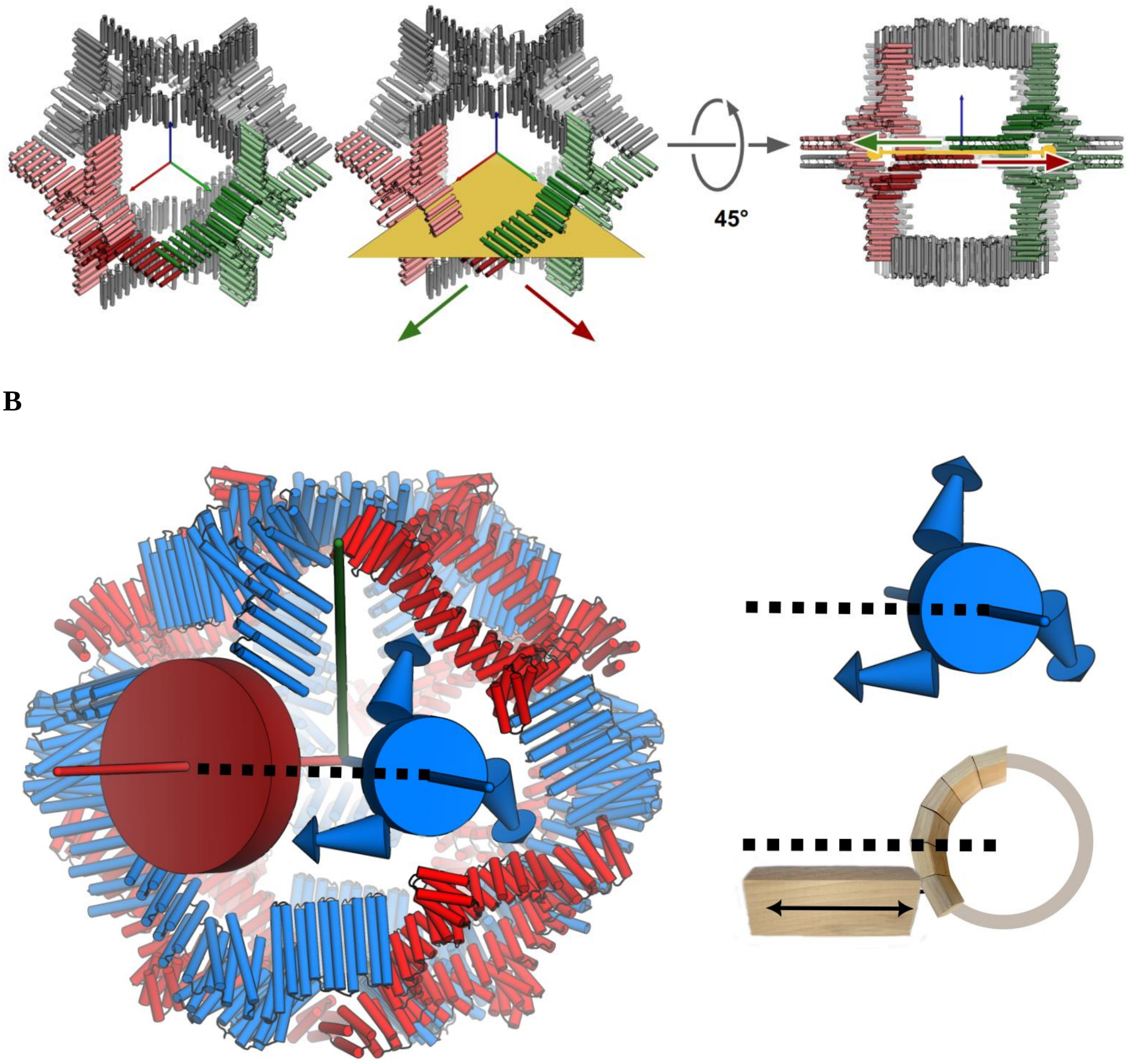

B

Fig. S11. Geometric constraints for expandable polyhedral symmetrical assemblies.

(A) An O4 octahedral nanocage showing the 4-fold symmetry axes aligned to the coordinate axes x, y, and z (red, green, and blue, respectively). The THR propagation vector (large red and green arrows) must be parallel to the plane (in yellow) formed by the two encompassing symmetrical axes, x and y. Deviations off this plane will cause contractions and extensions of the THR to clash and/or not connect to its symmetrical partners using the same contacts. (B) Similarly for a two-component architecture (O43 in this case), the component with the THR propagation vector (3D blue arrow) must remain parallel to the plane created by the 3-fold axis (blue) and 4-fold axis (red).

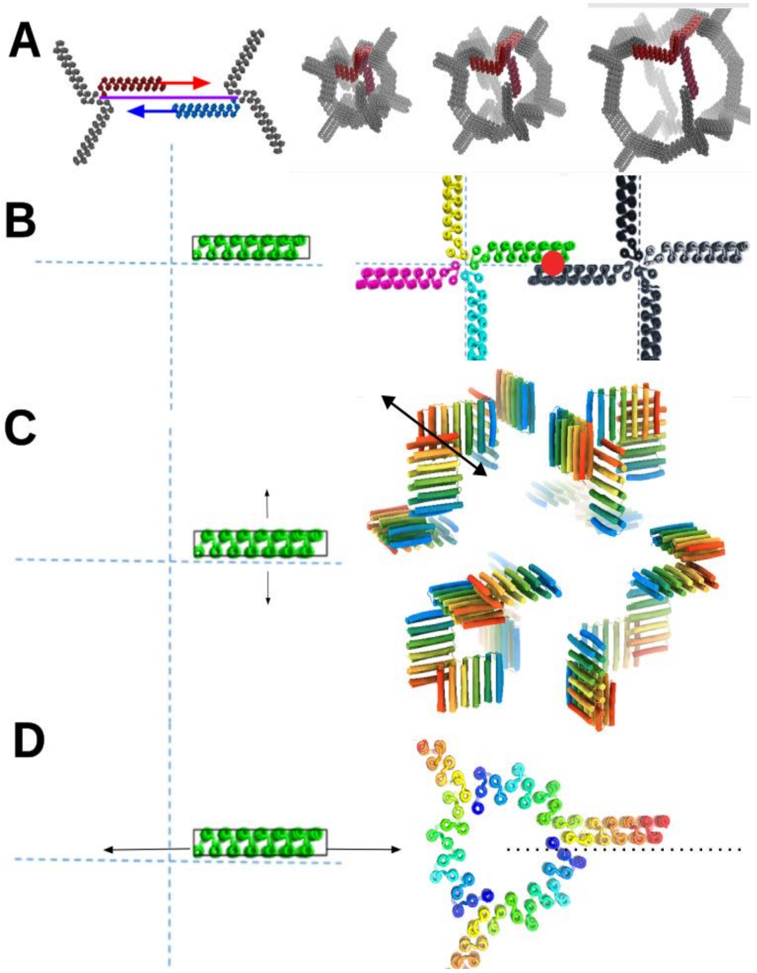

Fig. S12. Design strategy for THR Handshake nanocages

(A) To maintain the expandability of the cages, the THR propagation vector (red and blue) must be maintained parallel to the plane generated by the symmetry axes (purple). **(B)** the interface formed between the two THRs is the "handshake" interface (red dot). **(C)** Handshake C2 interfaces with angles corresponding to the desired architecture are designed by sampling the THR in the "y-direction" (sideways, but parallel to the required plane, and still within handshake contact range if C2 were applied), then treating that positioned THR as a cyclic oligomer for running RPXDock in nanocage symmetries, such as the O4 output shown to the right. The sampling allows Z movement of the THR, which manifests as sliding (arrow) along the handshake interface to find the best rigid body position to design. **(D)** The same THR is sampled in the "x-direction" (forwards and backwards) to find good fusion positions to the cyclic oligomer using RPXDock Axle protocol.

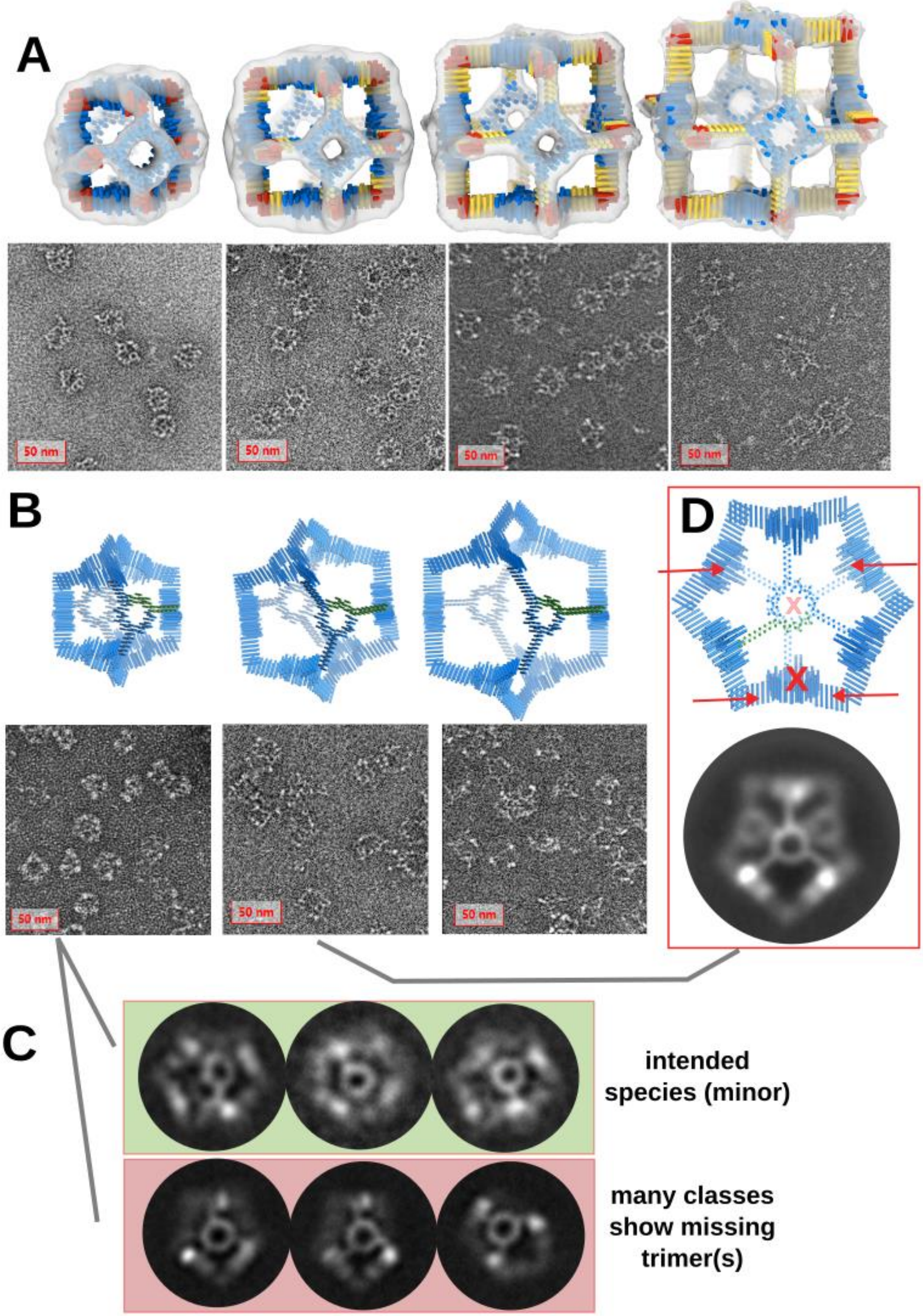

A
50 nm
50 nm
50 nm
50 nm
B
50 nm
50 nm
50 nm
D
C
intended species (minor)
many classes show missing trimer(s)

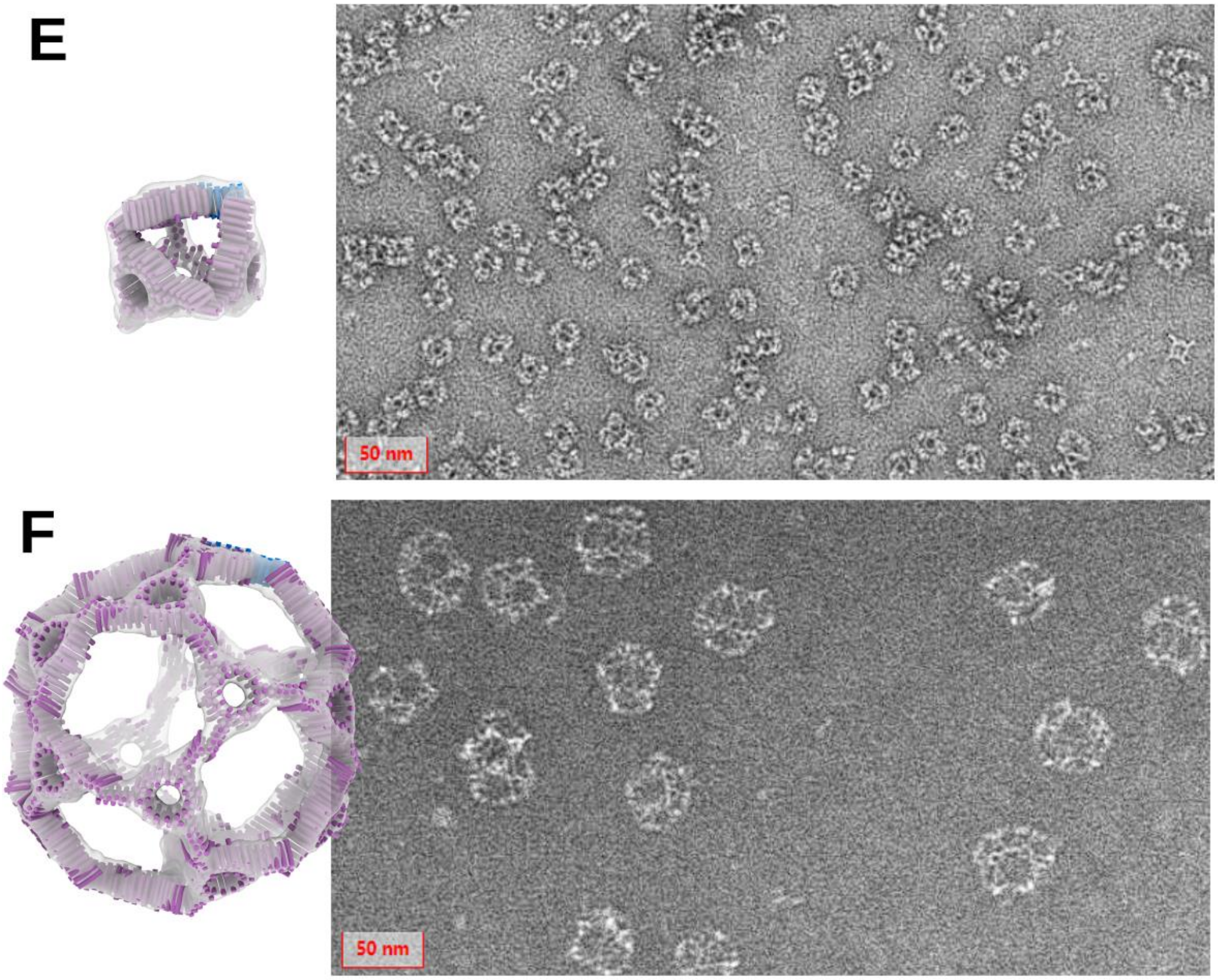

Fig. S13. Additional ns-EM data for THR Handshake nanocages

(A) Representative negative stain EM micrographs of each of the *cage_O4_34* sizes; +0, +4, +8, and +12 respectively. **(B)** Representative negative stain EM micrographs of the *cage_O3_2*0 sizes; +0, +4, and +8. **(C)** Class averaging of the base size shows populations of both the intended octahedral species and off-target populations with missing trimers. **(D)** Class averaging of the two larger sizes shows a monodisperse off-target population where two of the trimeric units are missing from the octahedral structure. **(E)** Representative negative stain EM micrograph of cage_T3_101 showing clear particles. **(F)** Representative negative stain EM micrograph of cage_I3_8 show clear particles, but some particles appear to be missing trimeric subunits.

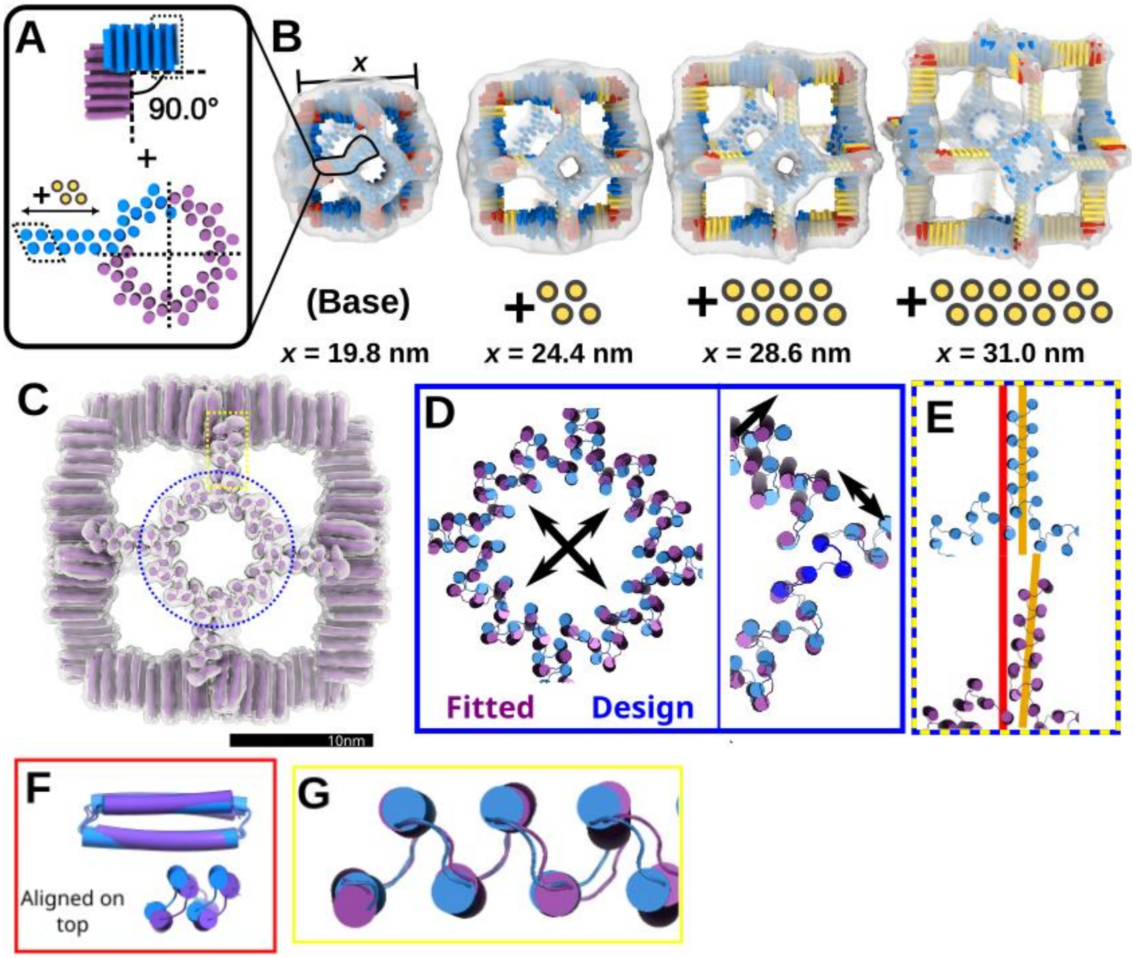

Fig. S14. Cryo-EM analysis of a modular O4 nanocage (*cage_O4_34*).

(A) Previously described (Fig. 4) construction method of combining two modules to make the nanocage. **(B)** The O4 octahedral handshake nanocage was characterized at 4 sizes with ns-EM reconstruction map overlaid with design models. Yellow represents regions that were extended by locally repeating the linear THR structure/sequence. A distance "x" is measured across the 3D reconstructions of each size. **(C)** Comparison of (left) the full O4 design model in blue and (right) the fitted model in purple in the cryo-EM map. Dashed regions indicate areas of focus in panels D-G. **(D)** Overlay of Handshake models aligned on one Hand to exaggerate the shift observed in the other Hand. **(E)** Overlay of Arm models aligned on the top 4 helices. **(F)** (left) Overlay of the Ring models to show general expansion of the ring. (middle) zoom-in on the Ring repeat element of the design model (Blue) fused to the Arm and making an interface with another Ring repeat on a different chain. Models are aligned on the blue repeat. (right) Overlay of the three Ring repeats in the fit (shades of purple) with the structural Ring repeat of the design model (blue). **(G)** Visualization of the Arm repeat axis (orange) for the Design and Fit models aligned along the C2 symmetry axis (red).

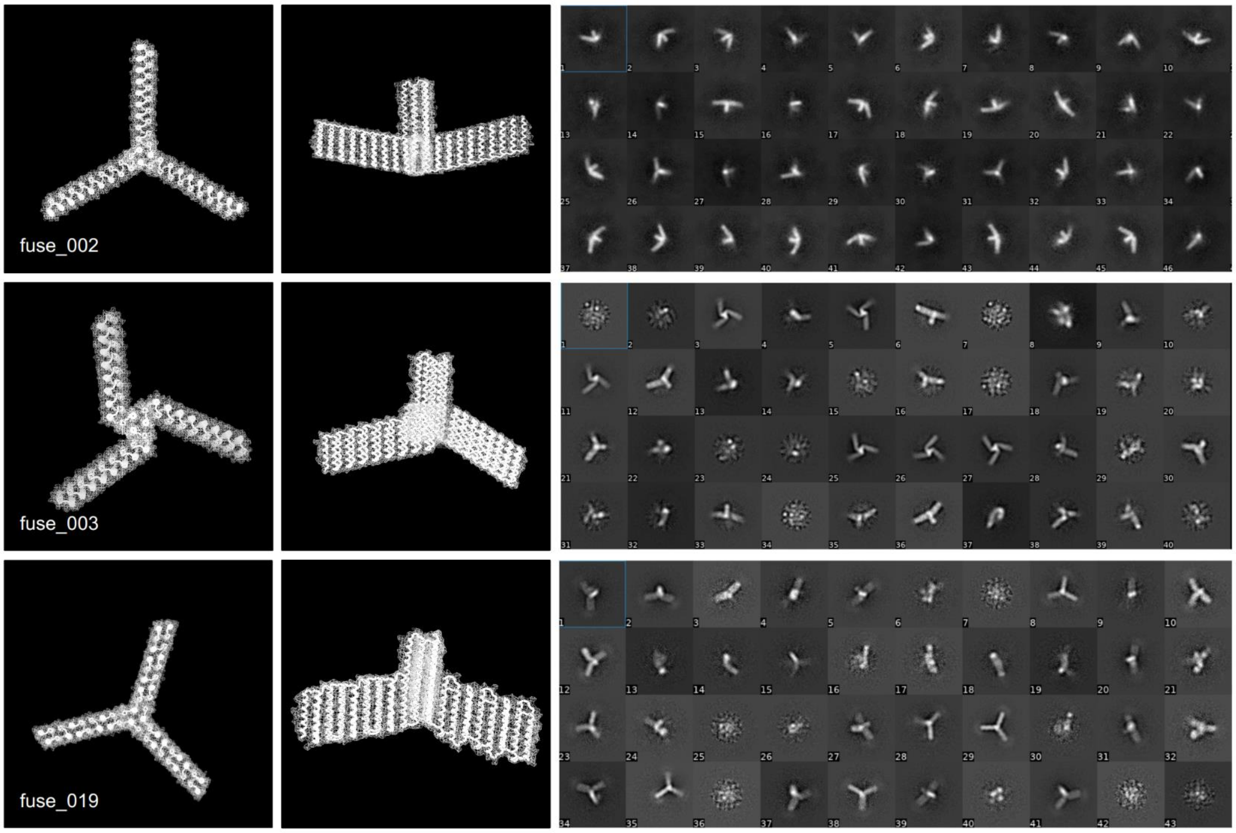

Fig. S15. Examples of HelixFuse *de novo* helical bundles to THR proteins.

(left) Design models showing 3-fold axis and side view. (right) Raw 2D class averages from negative stain electron microscopy showing many classes resembling the design model

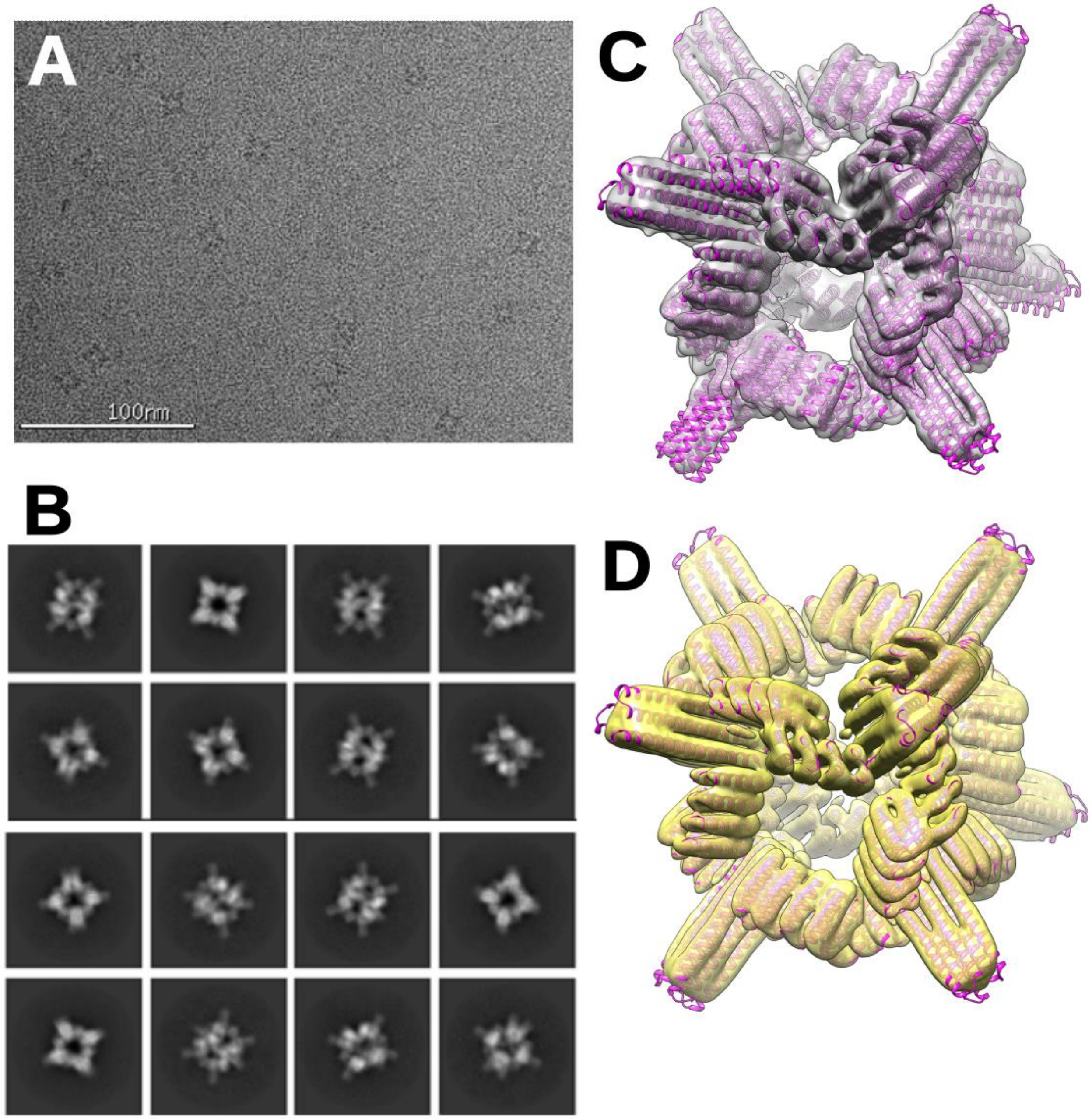

Fig. S16. Cryo-EM of *cage_O3_10*.

(A) Representative cryo-EM micrograph of *cage_O3_10*. (**B**) Representative 2D class averages. **(C)** Cryo-EM map (gray) without symmetry imposed (C1) at 8.2 Å resolution with the design model (magenta) fit in as a rigid body **(D)** Cryo-EM map (yellow) with O symmetry at 7.0 Å resolution with the design model (magenta) fit in as a rigid body.

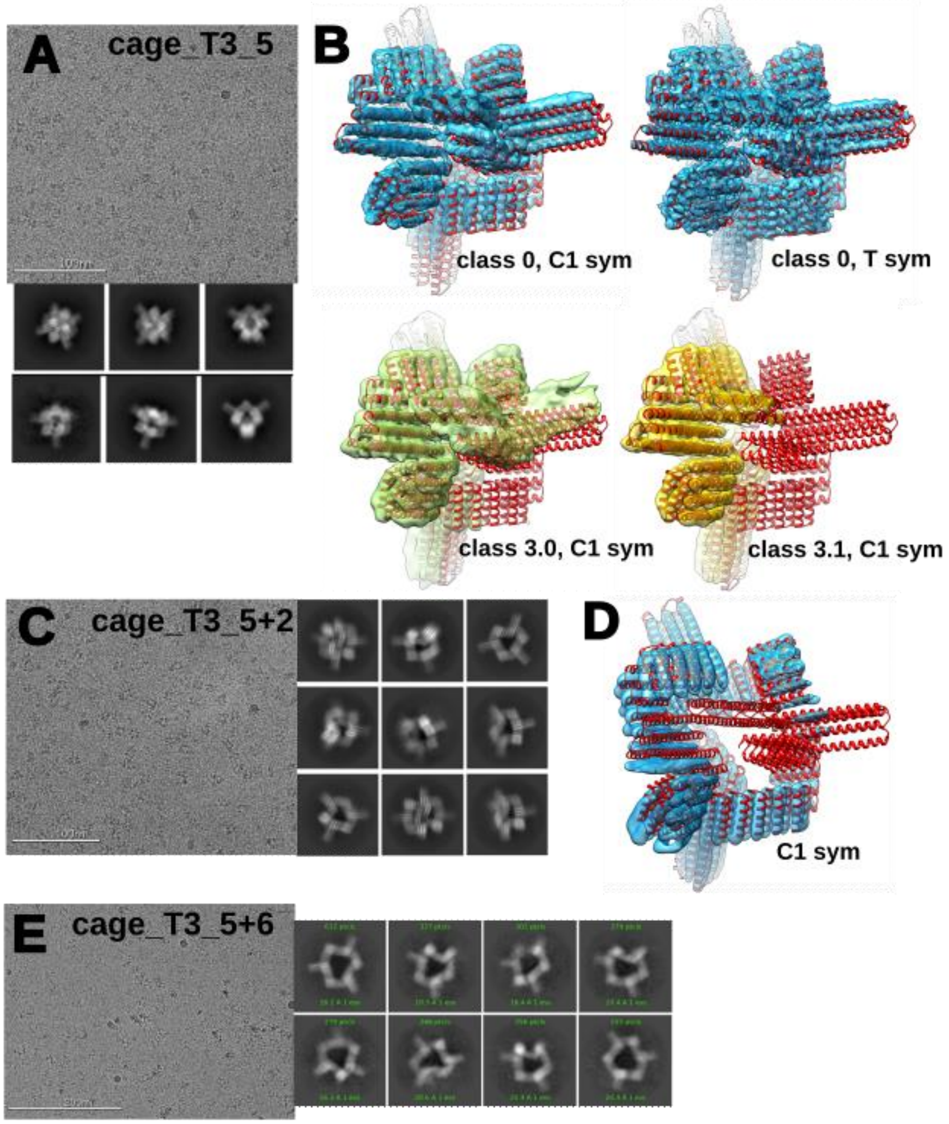

Fig. S17. Cryo-EM of cage_T3_5 and its expansions.

(A) Representative cryo-EM micrograph of *cage_T3_5*, and representative 2D class averages (scale bar = 100 nm). **(B)** Cryo-Em maps of *cage_T3_5* from different heterogeneous refinements as described in Figure S25, with design model (red) fit in as a rigid body. **(C)** Representative cryo-EM micrograph of expanded *cage_T3_5_+2*, and representative 2D class averages (scale bar = 100 nm). (**D**) Cryo-EM map of *cage_T3_5_+2* without symmetry applied (C1), with the design model fit in as a rigid body. Density corresponding to one trimer is absent, suggesting that this trimer is not present in the cage at this size. **(E)** Representative cryo-EM micrograph of expanded *cage_T3_5_+6*, and representative 2D class averages (scale bar = 200 nm).

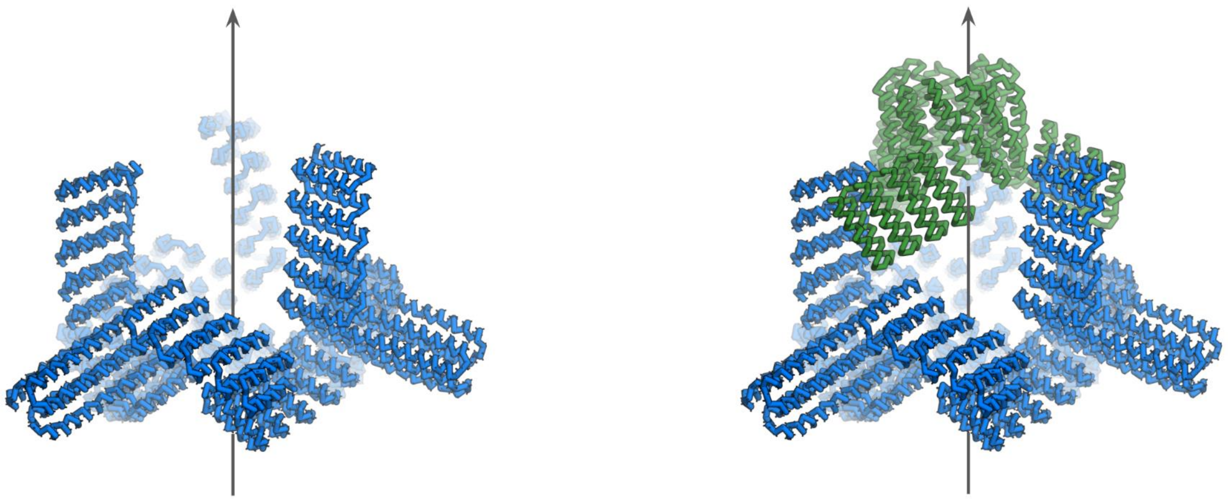

Fig. S18. Cage polarization scheme using cage_T3_5_+2.

Conceptual illustration to show that if a model of an incomplete cage were to be approximated from cryo-EM data into a simplified symmetry such as C3 (blue) aligned to the z-axis (left), it can be possible to design a new trimeric binding partner (green) that better satisfies the new opened configuration, resulting in breaking true tetrahedral symmetry into C3 cyclic symmetry.

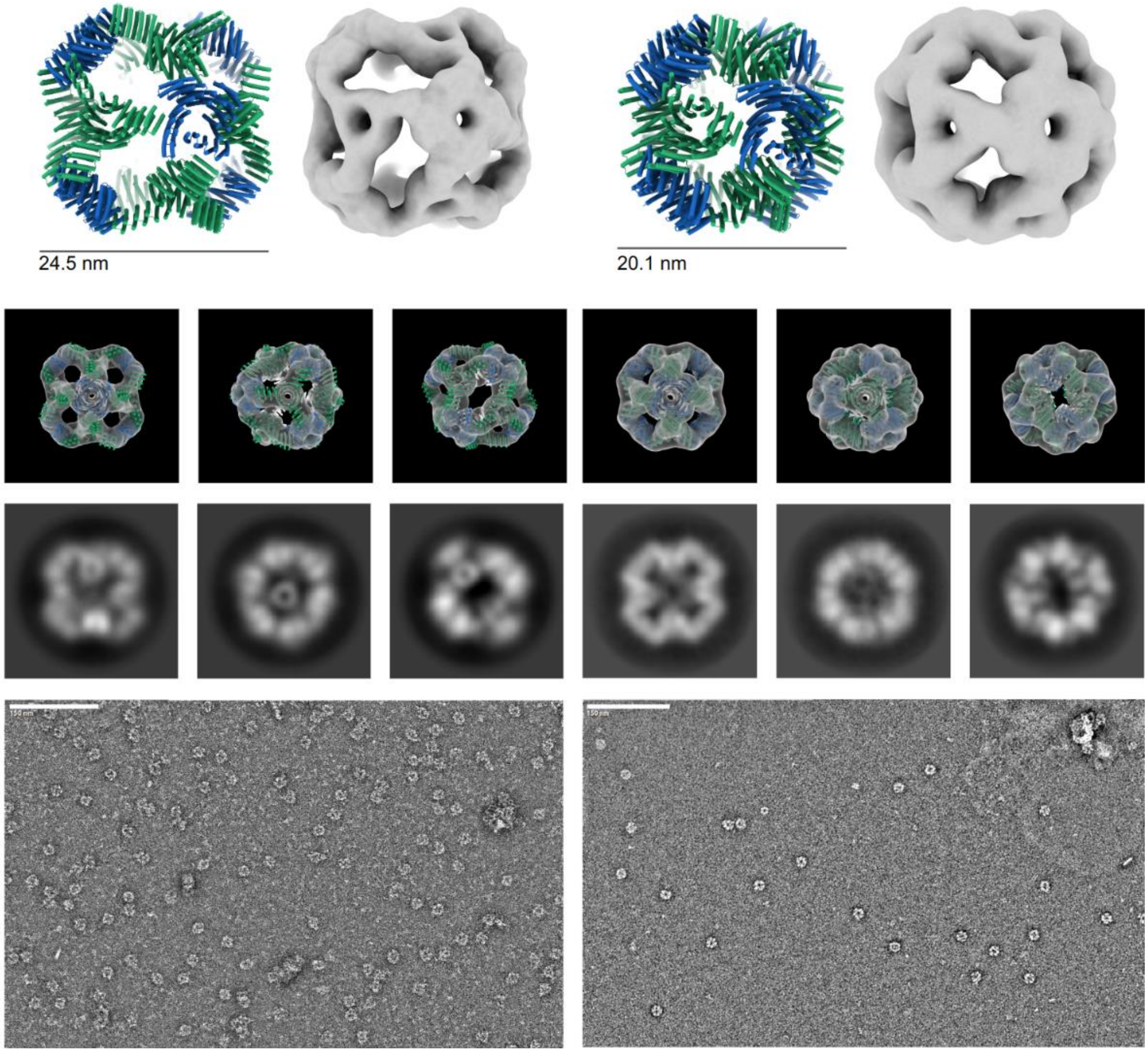

Fig. S19. Negative stain EM of cage_O43_54 series.

Negative stain electron microscopy of cage_O43_54 (left) and cage_O43_54_-4 (right). Design model with 3D reconstruction (top), design model with reconstruction map overlaid compared to selected class averages from each of the axes views (4-fold, 3-fold, and 2-fold) (middle), and a representative micrograph at 57,000x magnification (bottom); scale bar = 150 nm.

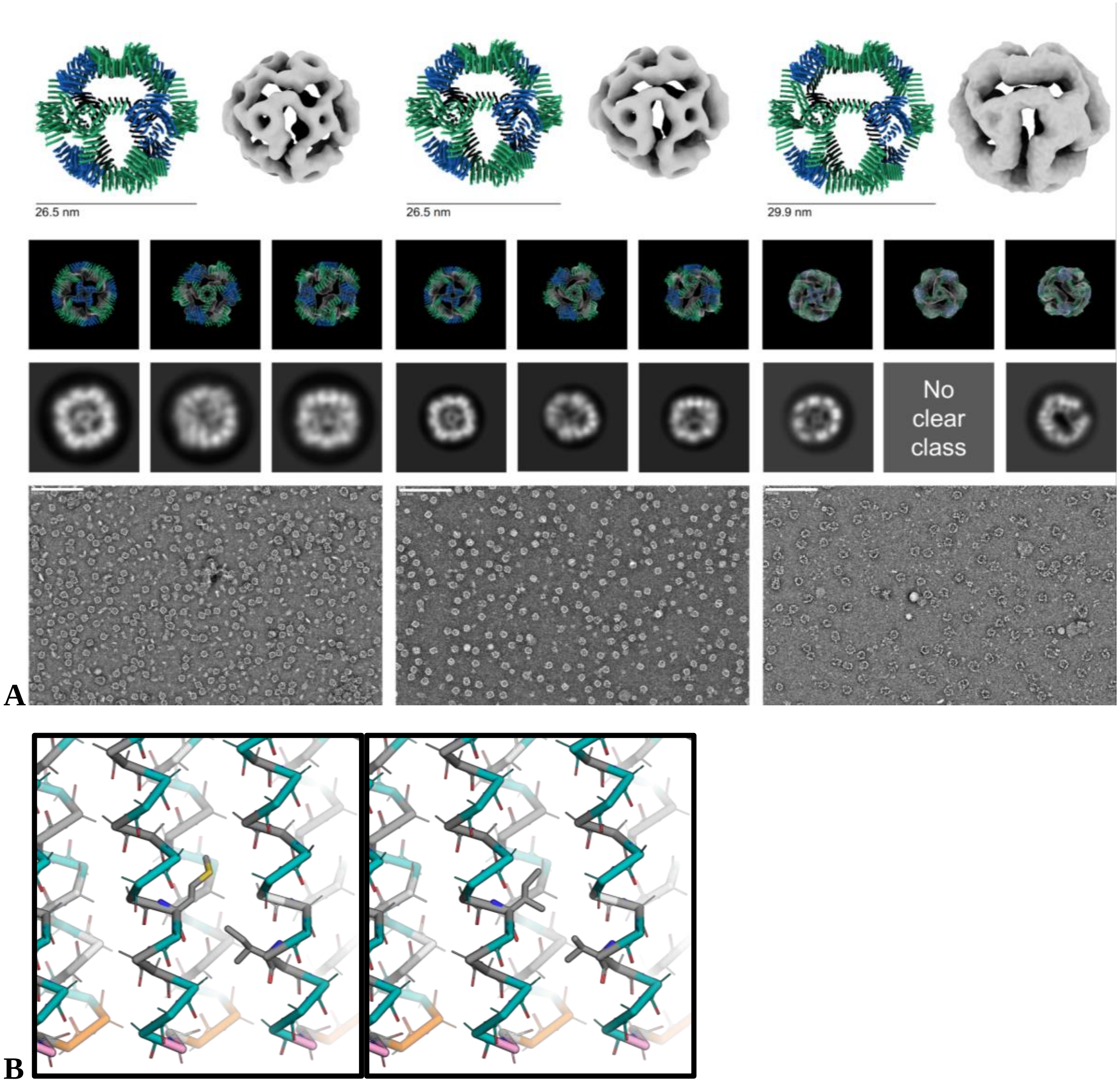

Fig. S20. Negative stain EM of cage_O43_59 series.

(A) Negative stain electron microscopy of cage_O43_59 (left), cage_O43_59_noMet (middle), and cage_O43_59_+4 (right). Design model with 3D reconstruction (top), design model with reconstruction map overlaid compared to selected class averages from each of the axes views (4-fold, 3-fold, and 2-fold) (middle), and a representative micrograph at 57,000x magnification (bottom); scale bar = 150 nm. The cage_O43_59_noMet construct is identical to cage_O43_59 except for methionine related mutations, and a disulfide pair to stabilize the terminal helix. **(B)** Original backbone of THR4 (left) and noMet mutant (right). Backbones of polar residues shown in teal, proline in orange, glycine in pink, alanine in white, and hydrophobic residues in gray. Each methionine (one per repeat, first instance M34) can be mutated to isoleucine and the nearby isoleucine (first instance I78) mutated to valine to compensate without disrupting the structure.

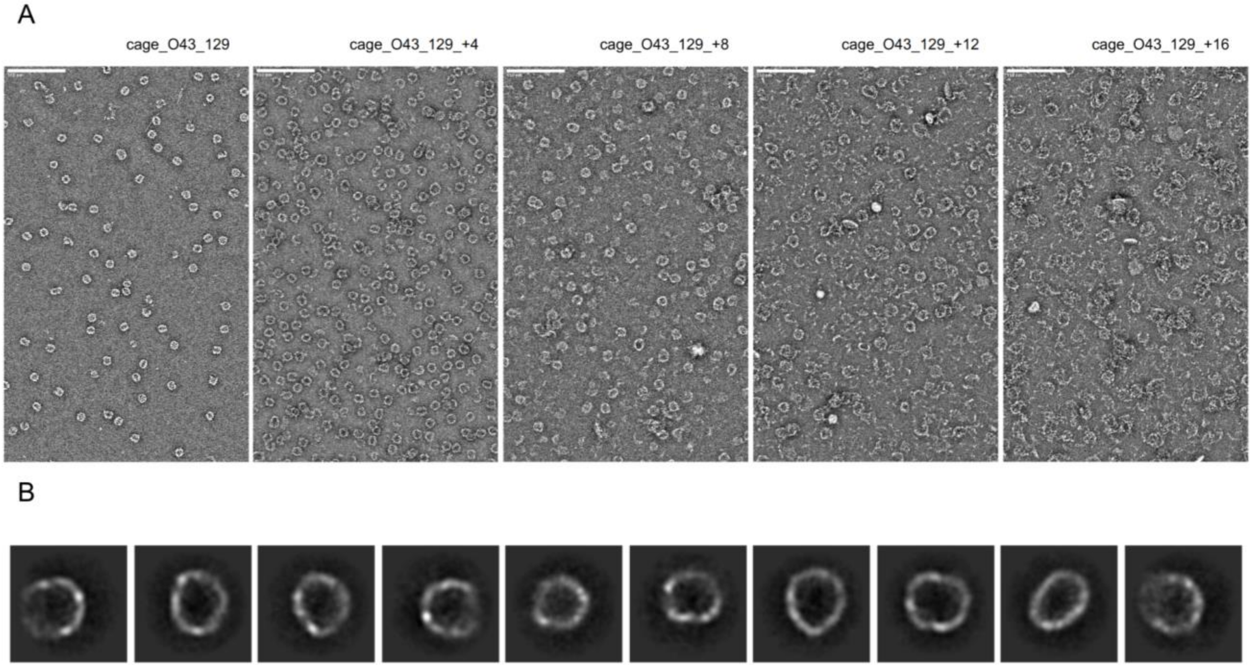

Fig. S21. Negative stain EM of cage_O43_129 series.

(A) Negative stain electron microscopy micrographs of cage_O43_129 (left) to cage_O43_129_+16 (right). Increased amounts of unassembled and/or misassembled protein in the background increases with extended THR arm length. (B) Representative class averages of cage_O43_129_+16. Although the design yielded distinct pickable particles, the resulting class averages were heterogeneous and often oblong, suggesting misassembled particles.

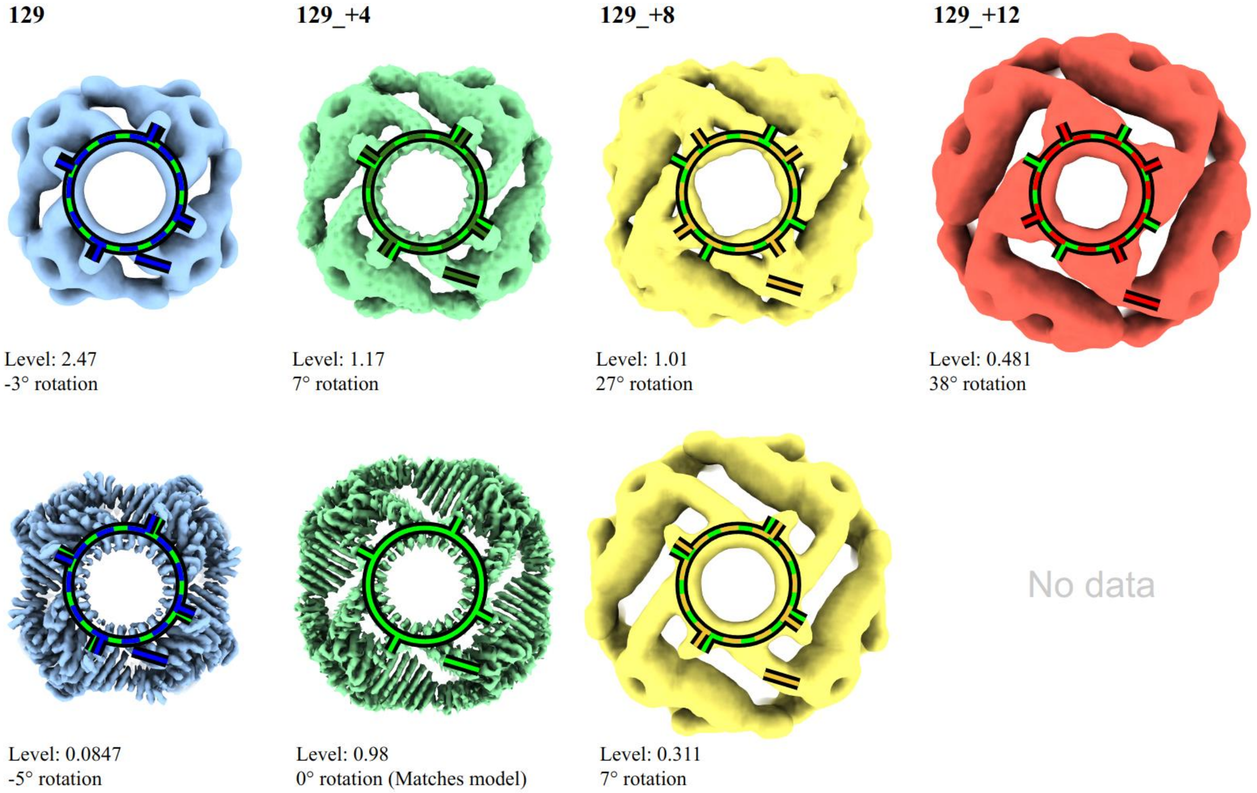

Fig. S22. Rotational deviation of cage_O43_129 series.

Left to right: 3D reconstruction maps of cage_O43_129 (blue), cage_O43_129_+4 (green), cage_O43_129_+8 (yellow), and cage_O43_129_+12 (red) visualized along the 4-fold symmetrical axis. Top row is ns-EM; bottom row is cryo-EM. The C4 component rotation that matches the design model is shown as a light green circle (cage_O43_129_+4 in cryo). At all size iterations the C4 component should be identical in rotation in theory. Rotations were approximated by eye to the center of the "arm" extruding from the C4 ring. The ns-EM reconstruction of +4 shows a 7° clockwise rotation of the C4 component (dark green) compared to the cryo-EM reconstruction. Both smaller versions show a counter-clockwise rotation relative to the design model (-3° for ns-EM and -5° cryo-EM). The larger +8 versions both show a clockwise rotation (27° for ns-EM and 7° cryo-EM). The largest +12 version rotates clockwise 38° with ns-EM. Cryo-EM for +12 was not collected due to sample heterogeneity. The disconnected colored line overlaid in each reconstruction depicts the trajectory of the THR on the C3 component. Overall, longer THR extensions led to more dramatic rotations, and ns-EM seems to be less reliable for assessing this effect compared to cryo-EM.

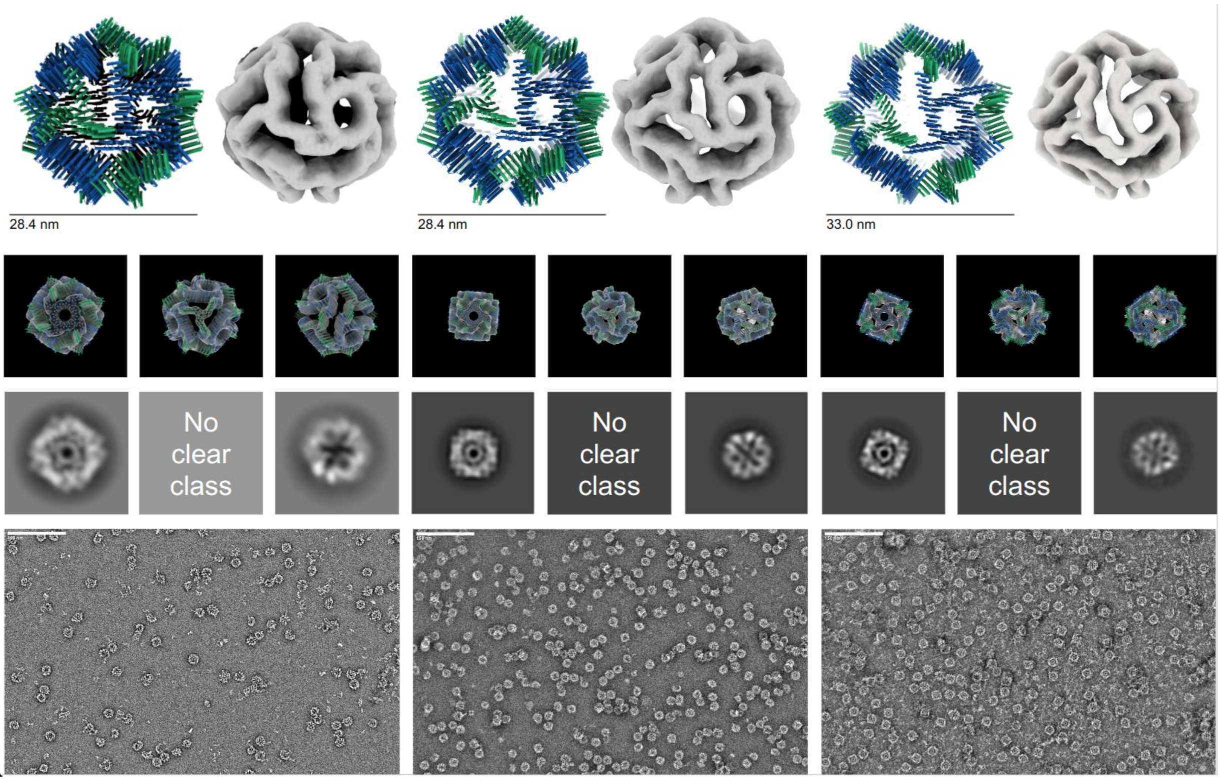

A

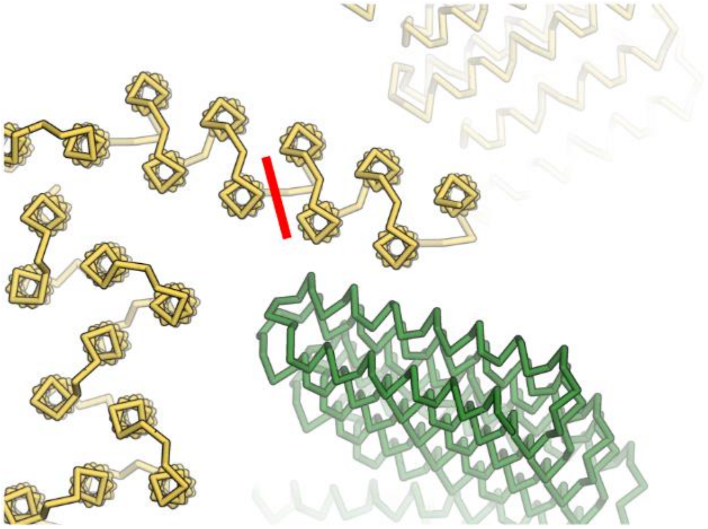

B

Fig. S23. Negative stain EM of cage_O43_164 series.

(A) Negative stain electron microscopy of cage_O43_164 (left), cage_O43_164_cut (middle), and cage_O43_164_+4_cut (right). Design model with 3D reconstruction (top), design model with reconstruction map overlaid compared to selected class averages from each of the axes views (4-fold, 3-fold, and 2-fold) (middle), and a representative micrograph at 57,000x magnification (bottom); scale bar = 150 nm. (B) ”Cut” variant expresses the first five helices of the C4 subunit as a separate chain; cut location depicted with the red line.

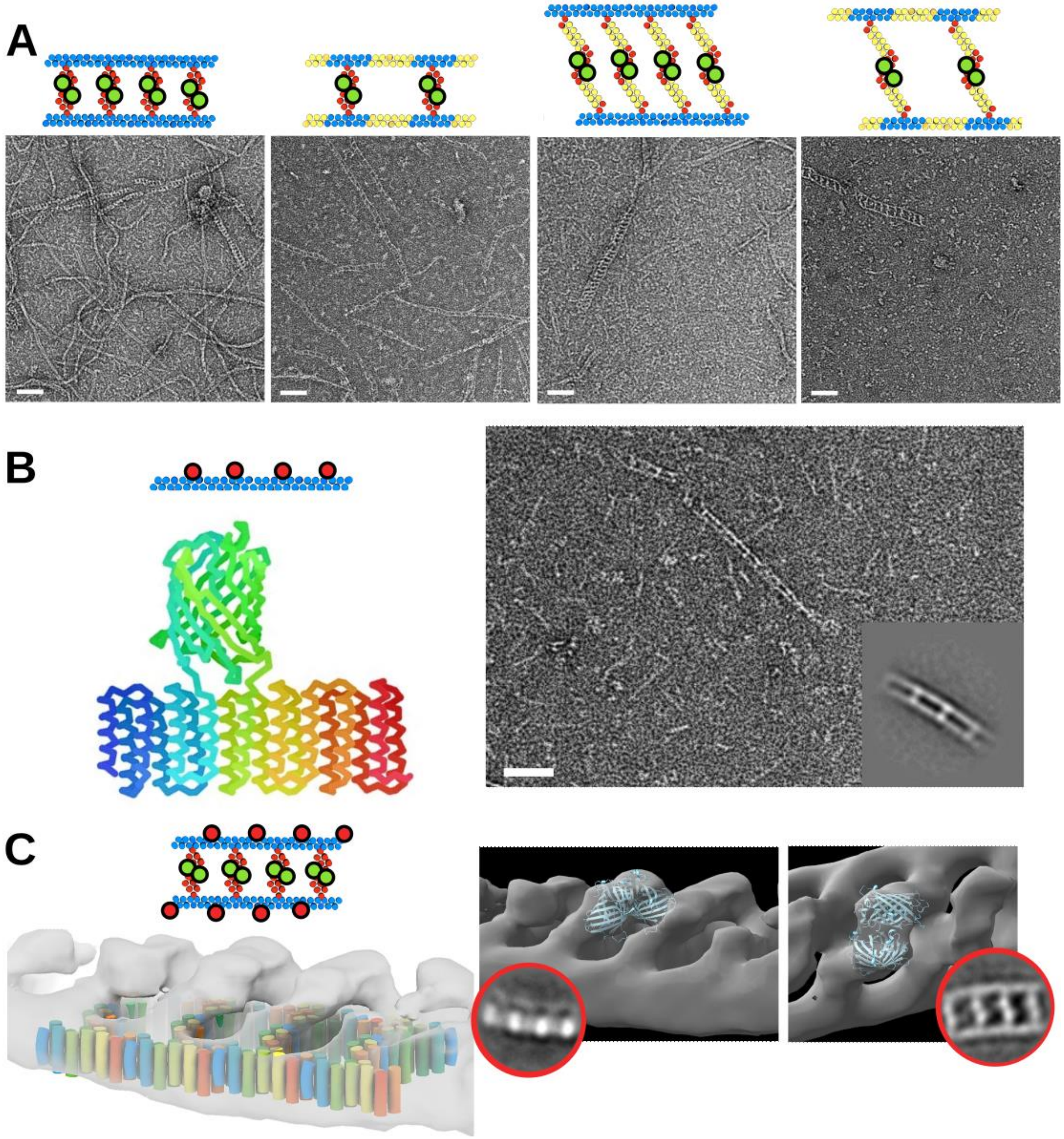
A
B
C

Fig. S24. Additional ns-EM data for train track designs.

(A) Comparison of assembly for the 4 sizes of train track with GFP fused to the C2 module, all prepared under the same conditions of guanidine HCl denaturation of both components and then dialysis into TBS buffer when both components are mixed in approximate equimolar ratio before dialysis. 50 nm white scale bars. (B) A version of the rail/ Split module THR where the interface on the side for the rail is knocked out, and instead a mScarlet sequence (*24)* is inserted into one of the middle loops (this protein is still uncapped so it can assemble long-ways). An Alphafold2 prediction of one such design monomer is shown in rainbow. A ns-EM micrograph (50 nm white scale bar) with class average inset shows that single fibrils rarely assemble end-to-end with more than 5 subunits, but perhaps weak dimerization of the mScarlet protein combined with the periodic templating of the rail leads to more stable double-fibrils. (C) An example of the base size train track where density of the GFP was visible in the ns-EM reconstruction. An Alphfold2 predicted sfGFP (*23*) dimer was fit into the density above the obvious train track pattern, and matched well.

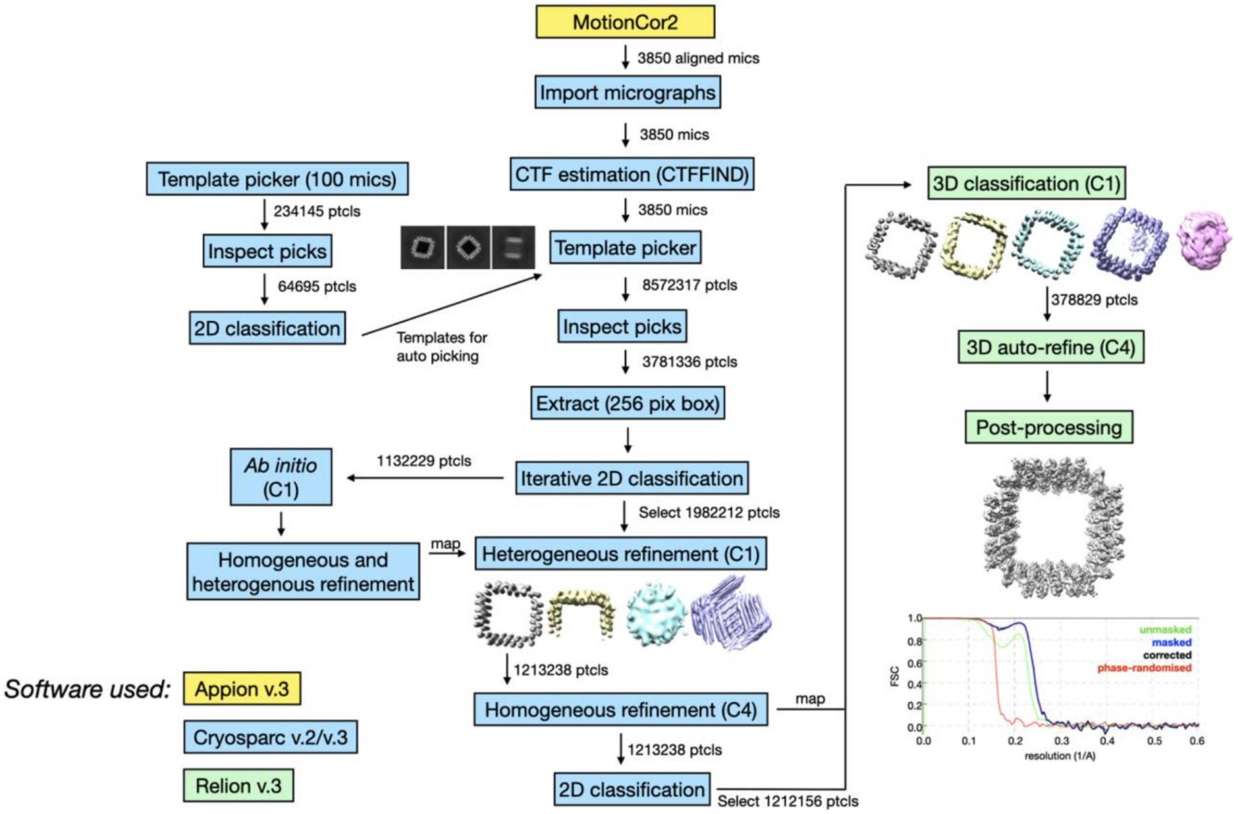

Fig. S25. cryo-EM data processing pipeline used for *sC4*.

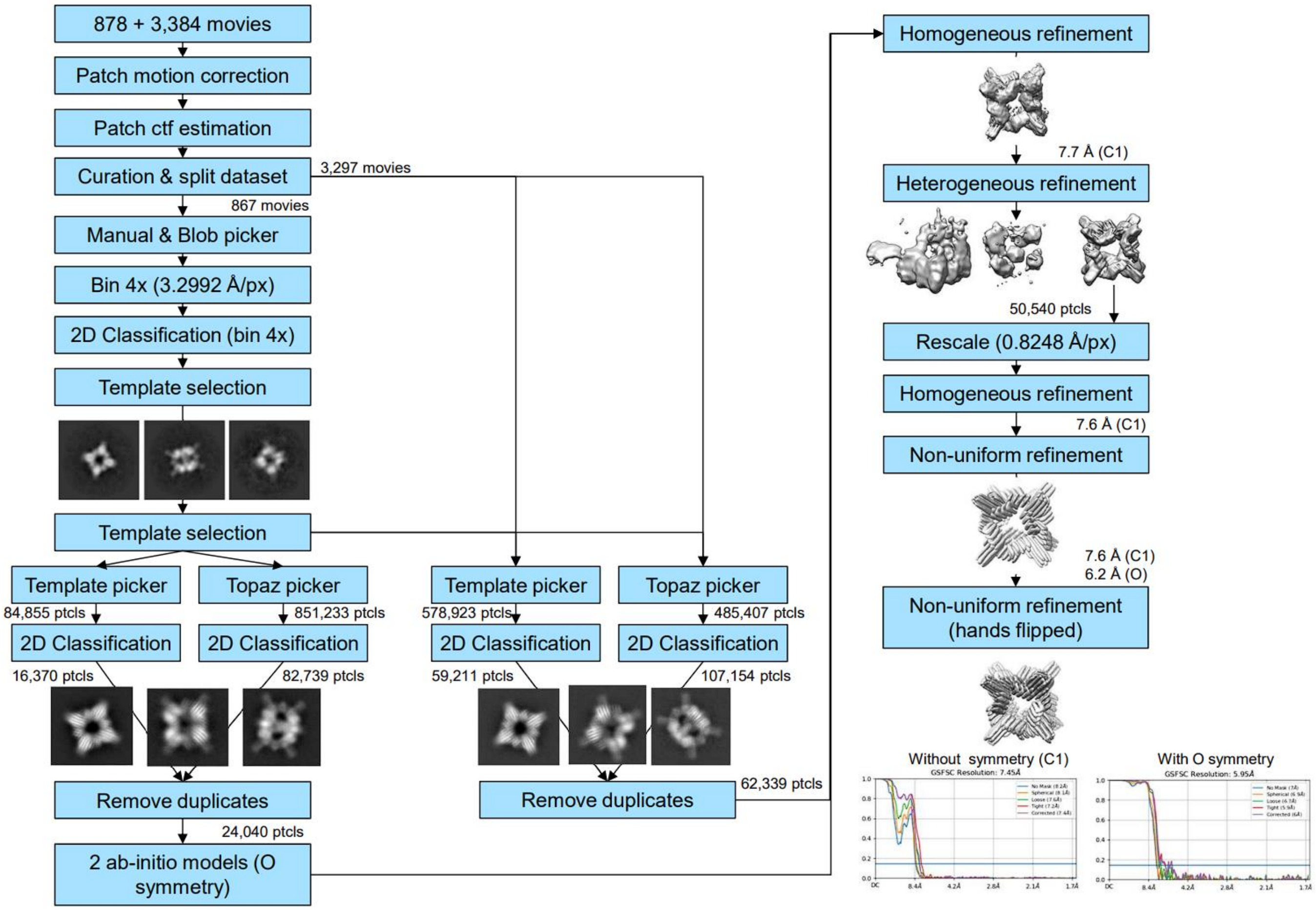

Fig. S26. cryo-EM data processing pipeline used for *cage_O3_10*.

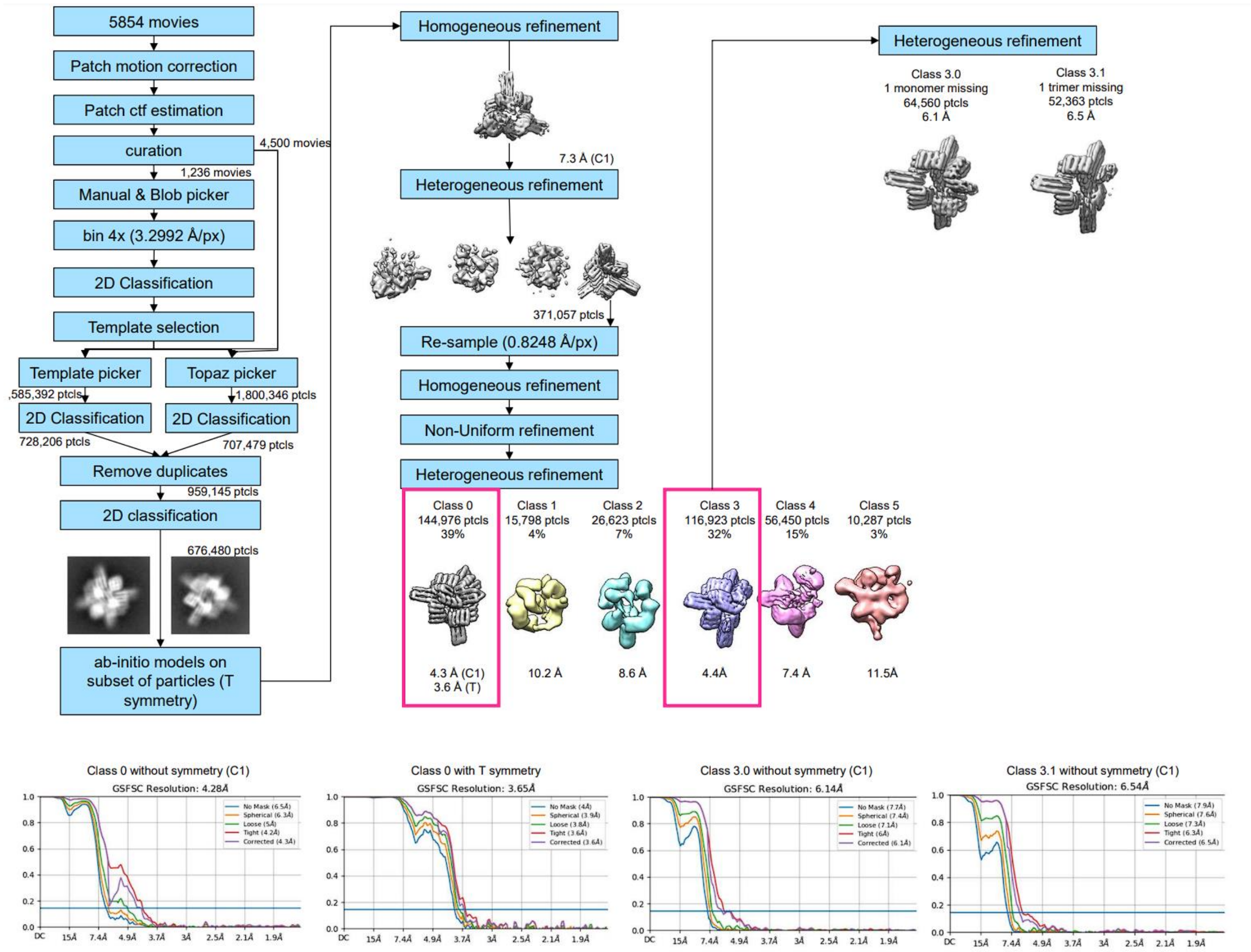

Fig. S27. cryo-EM data processing pipeline used for *cage_T3_5*.

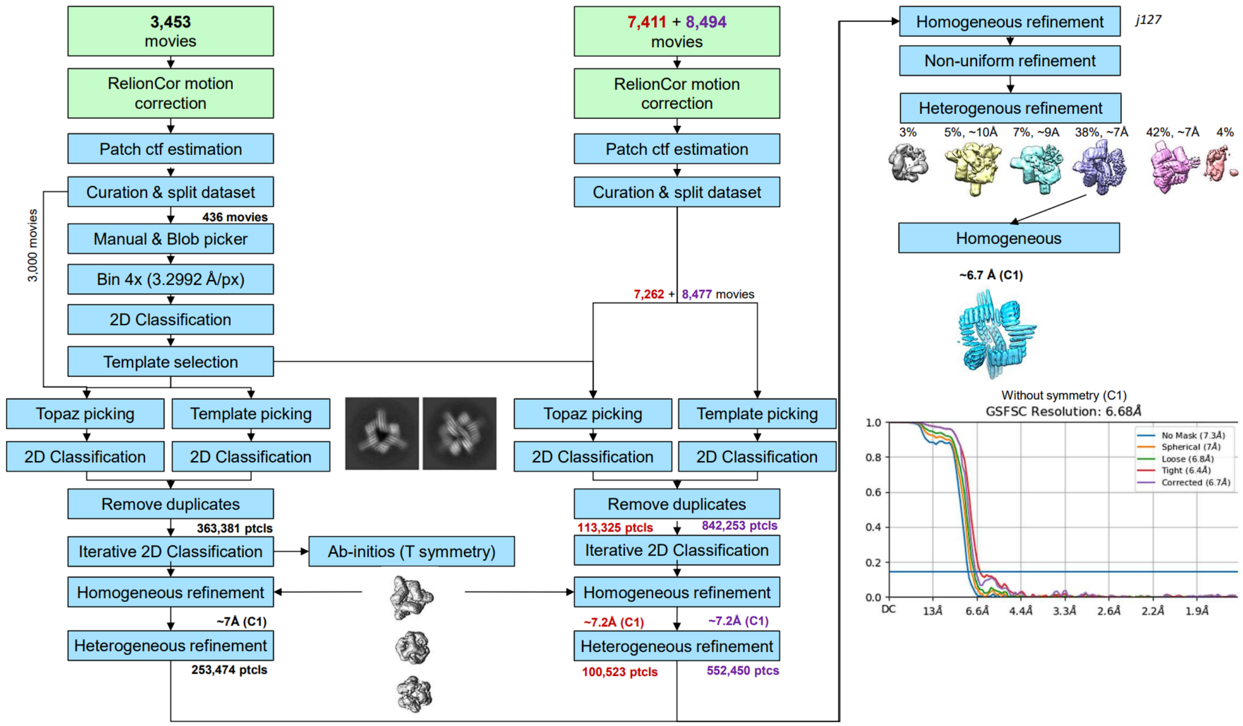

Fig. S28. cryo-EM data processing pipeline used for *cage_T3_5_+2*.

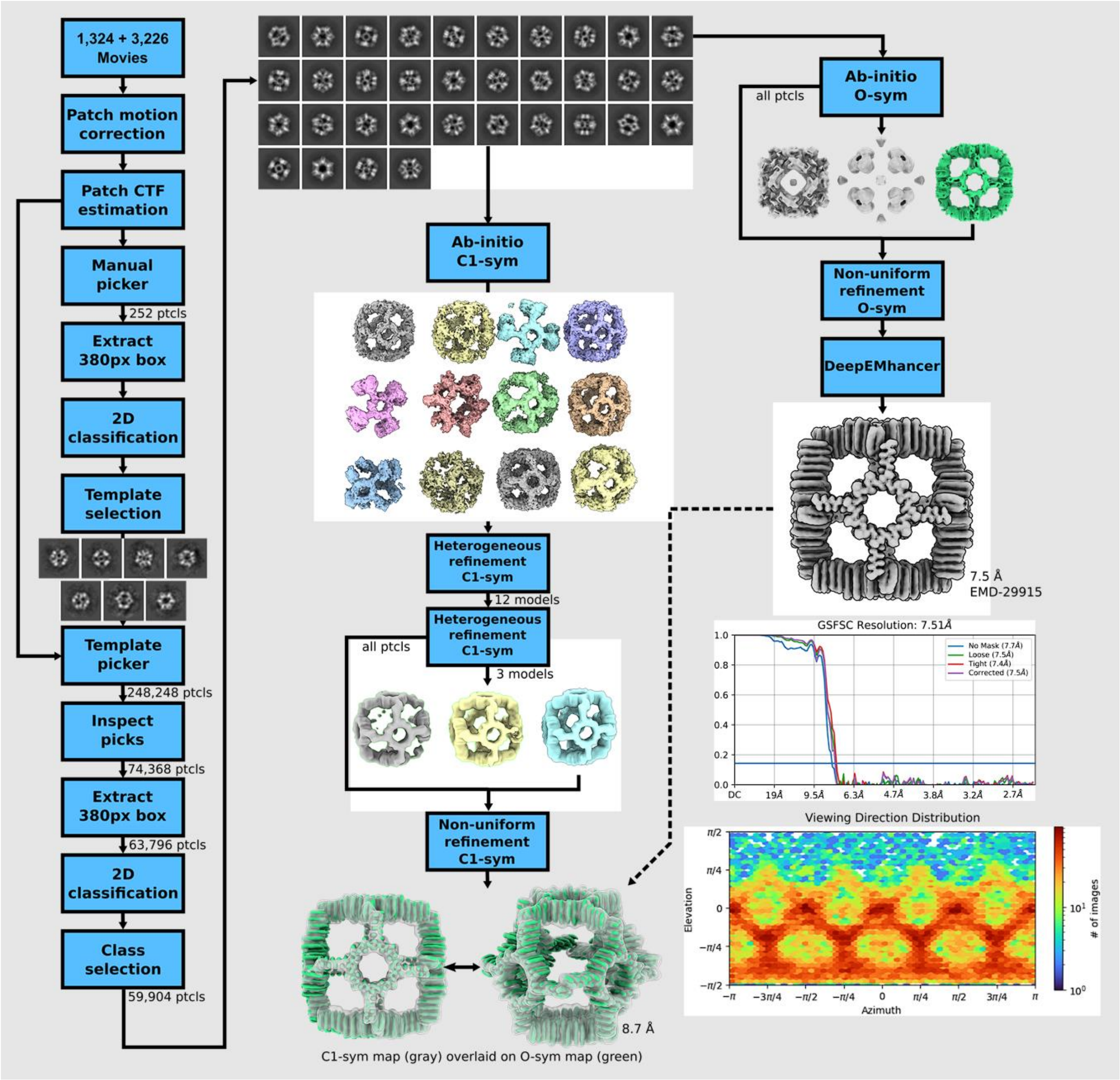

Fig. S29. cryo-EM data processing pipeline used for *cage_O4_34*.

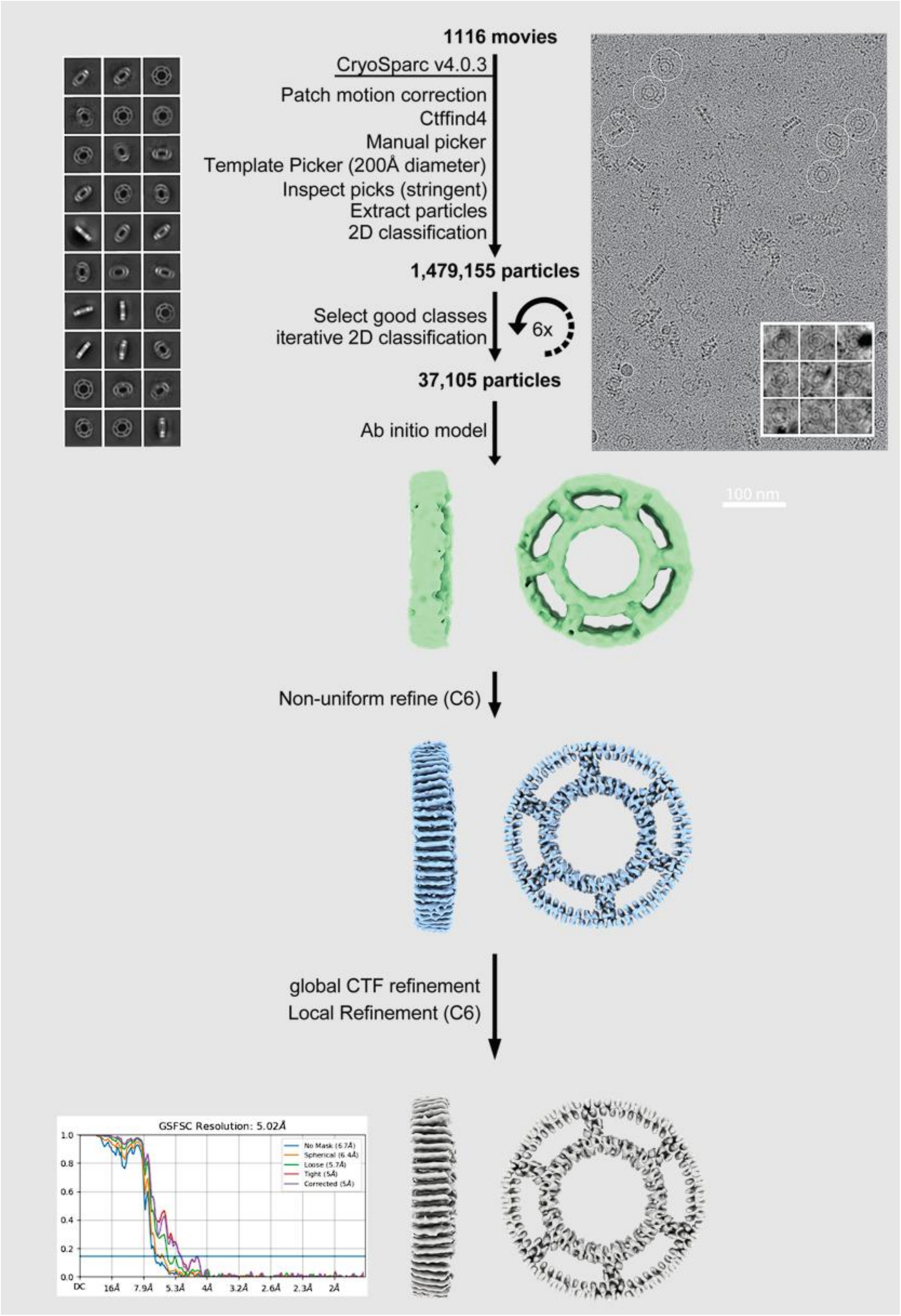

Fig. S30. cryo-EM data processing pipeline used for *strut_C6_21*.

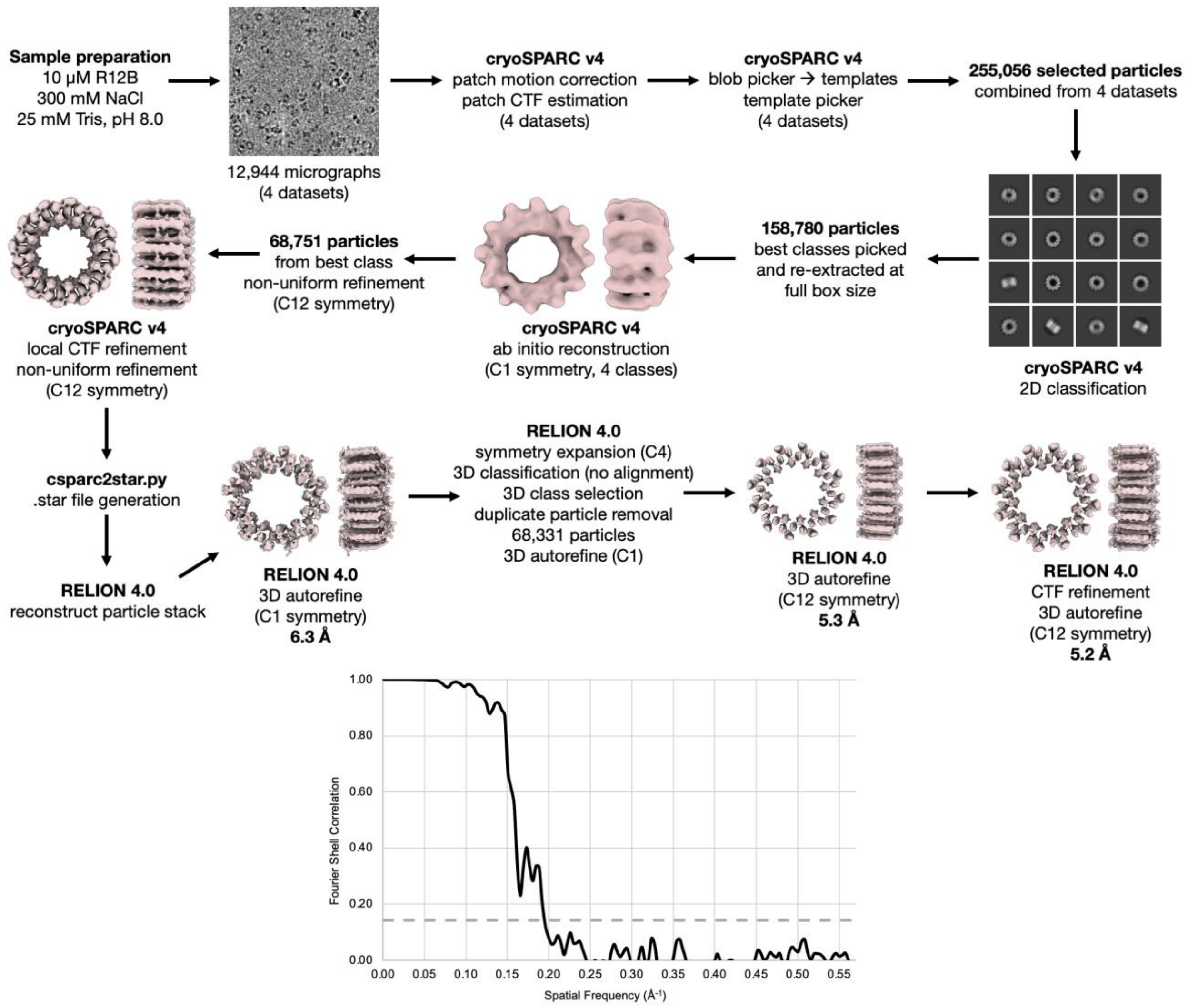

Fig. S31. cryo-EM data processing pipeline used for *R12B*.

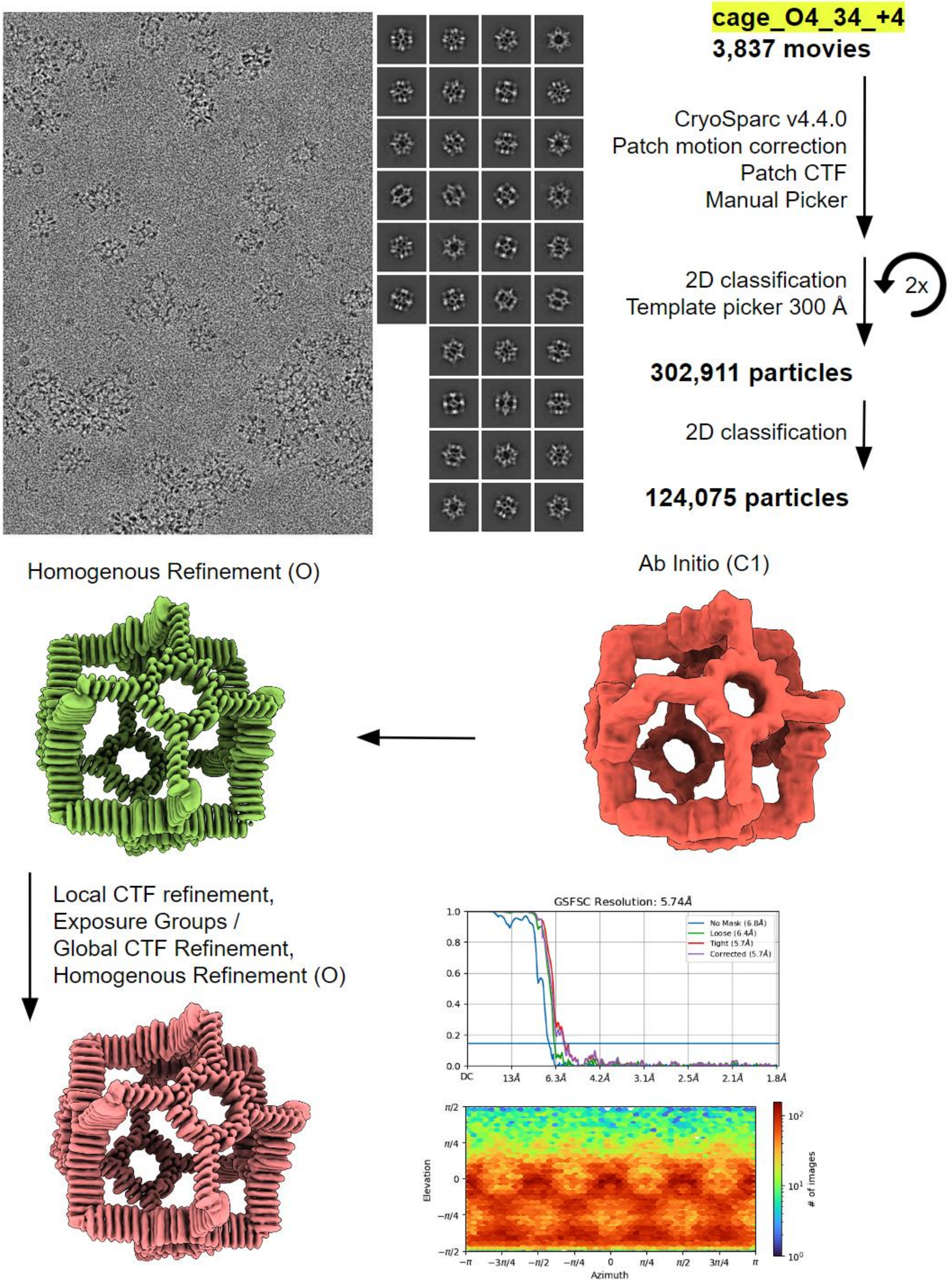

Fig. S32. cryo-EM data processing pipeline used for *cage_O4_34+4*.

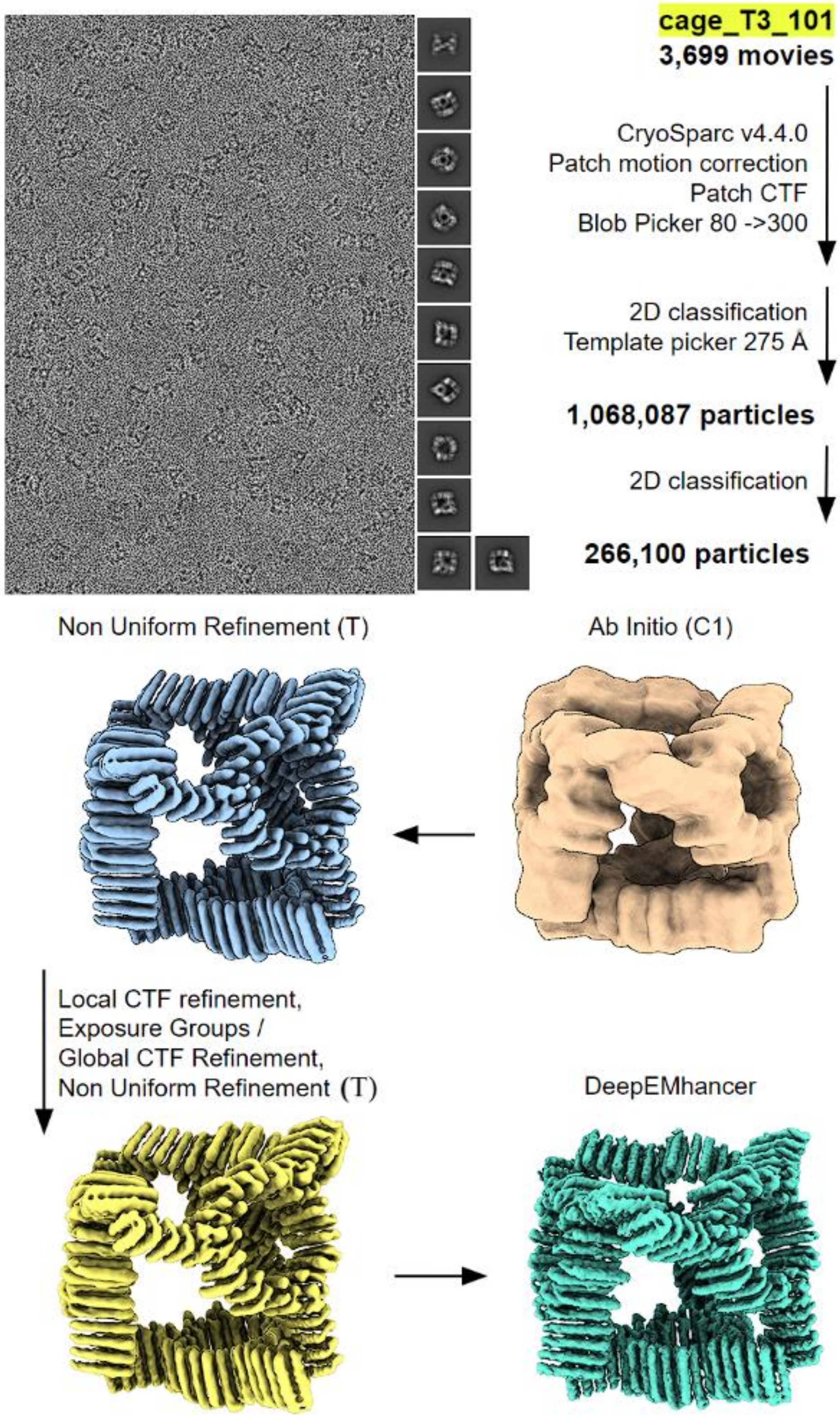

Fig. S33. cryo-EM data processing pipeline used for *cage_T3_101*.

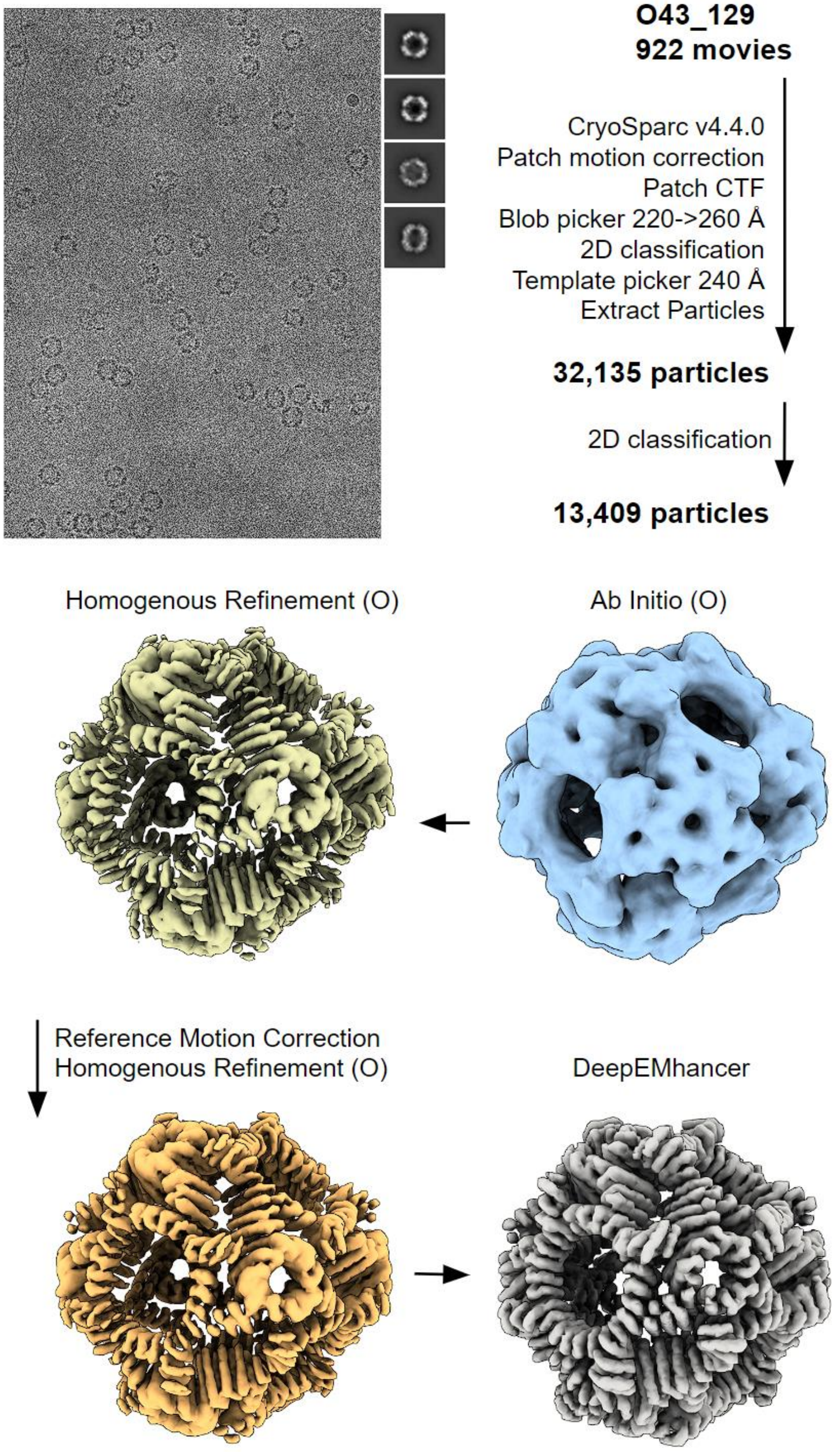

Fig. S34. cryo-EM data processing pipeline used for *cage_O43_129*.

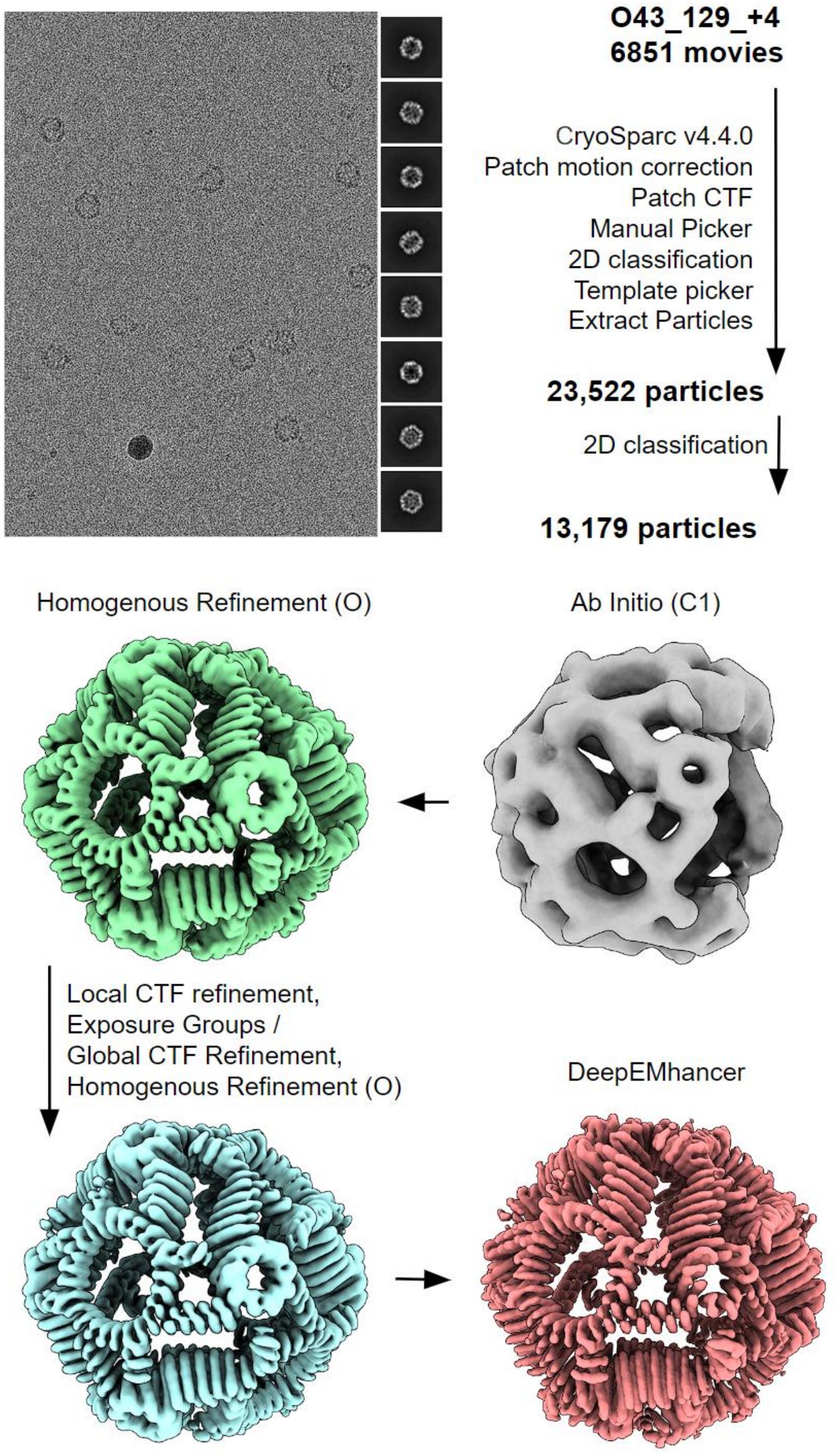

Fig. S35. cryo-EM data processing pipeline used for *cage_O43_129_+4*.

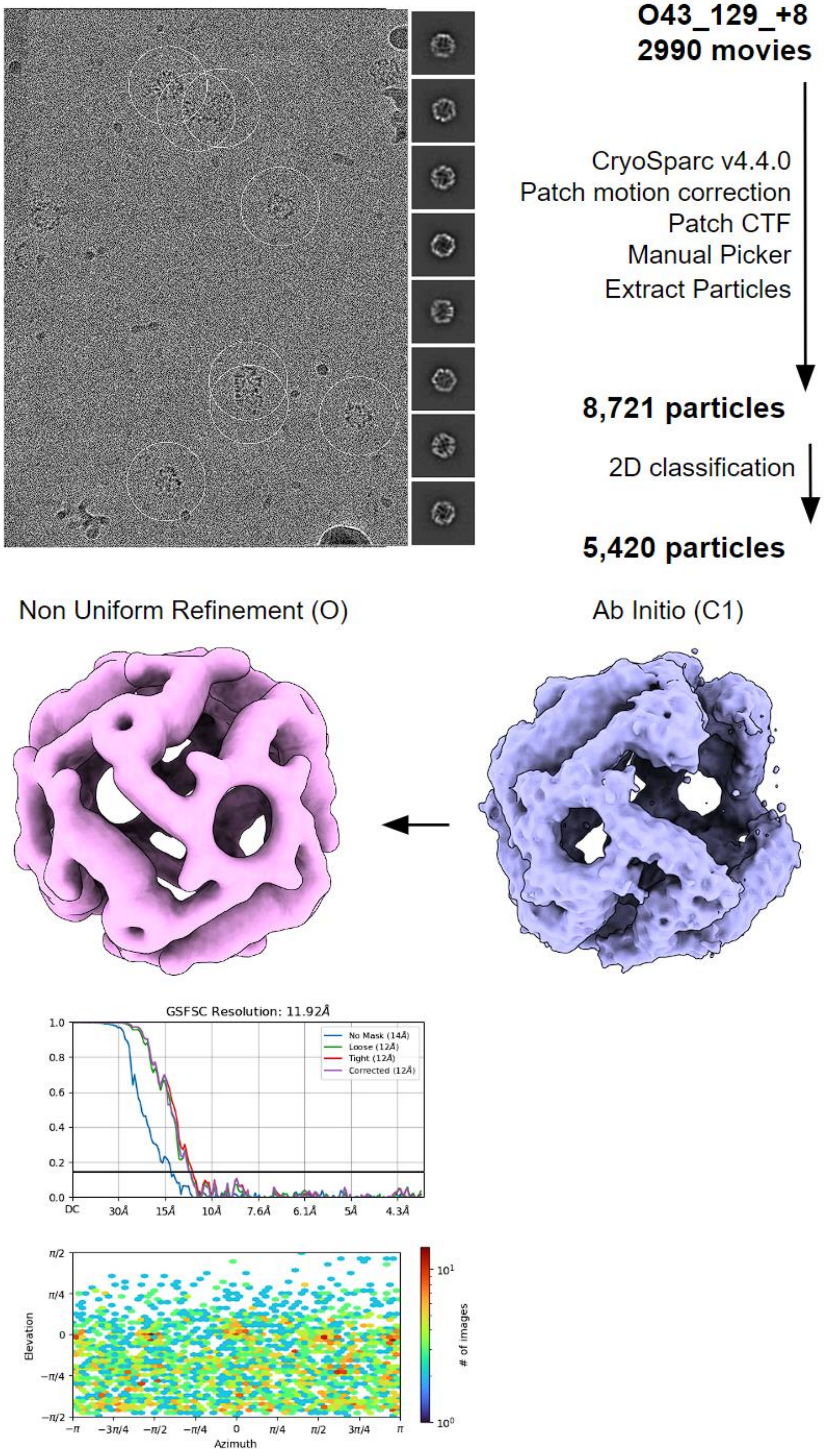

Fig. S36. cryo-EM data processing pipeline used for *cage_O43_129_+8*.

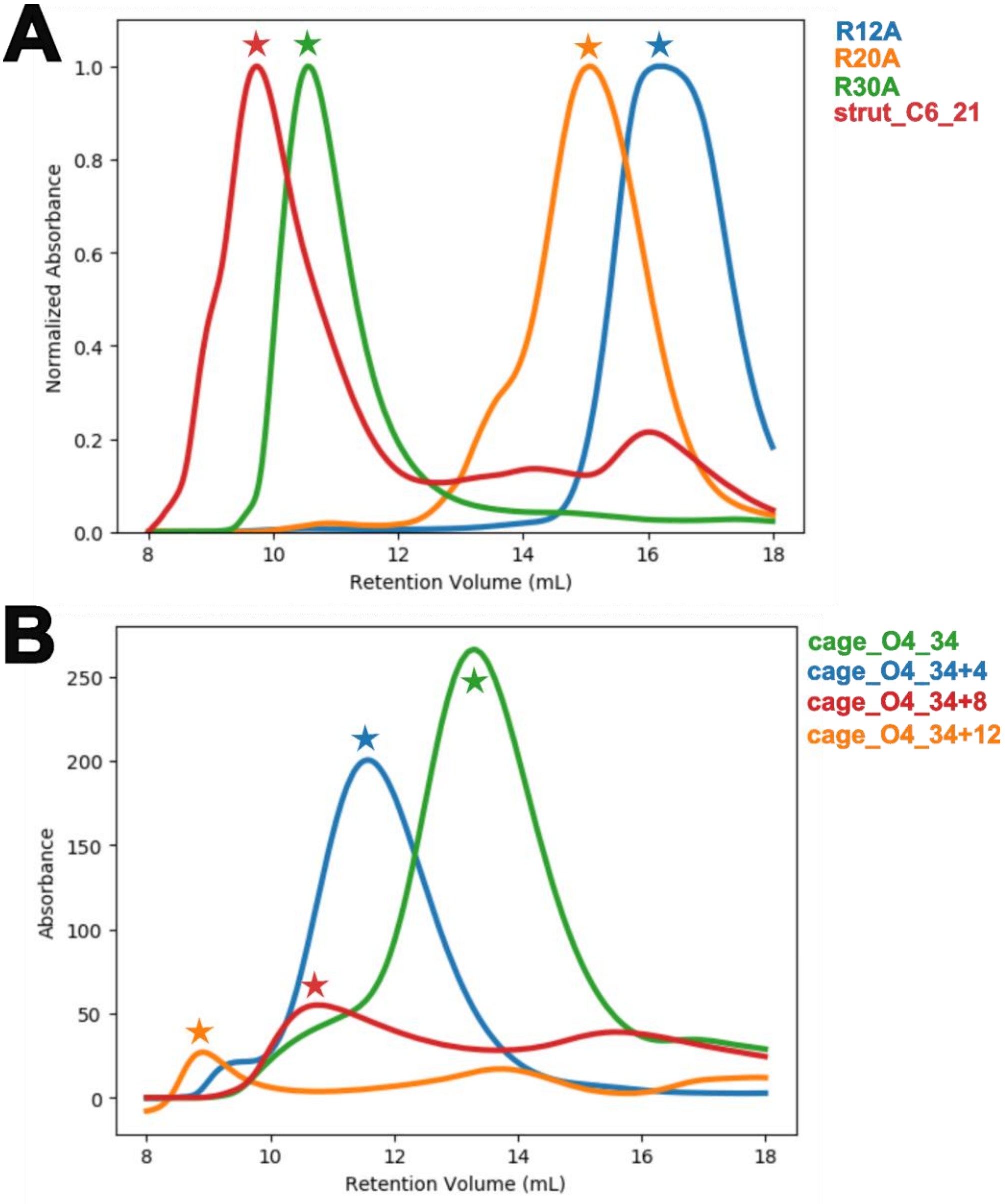

Fig. S37. Size Exclusion Chromatography (SEC) traces of representative designs

(A) Overlaid SEC traces of selected ring designs ran on Superdex® 200 Increase 10/300 GL column after IMAC purification. Absorbance at 230 nm wavelength is normalized between samples because not all were prepared at the same scale. *R12A*, *R20A*, and *R30A* are single component multimeric designs, and *strut_C6_21* is a 2-component multimeric design. (B) Overlaid SEC traces for 4 sizes of *cage_O4_34*, non-normalized absorbance at 230 nm wavelength, ran on Superose® 6 Increase 10/300 GL column. All samples in (B) were prepared from 50 mL culture with autoinduction for expression.

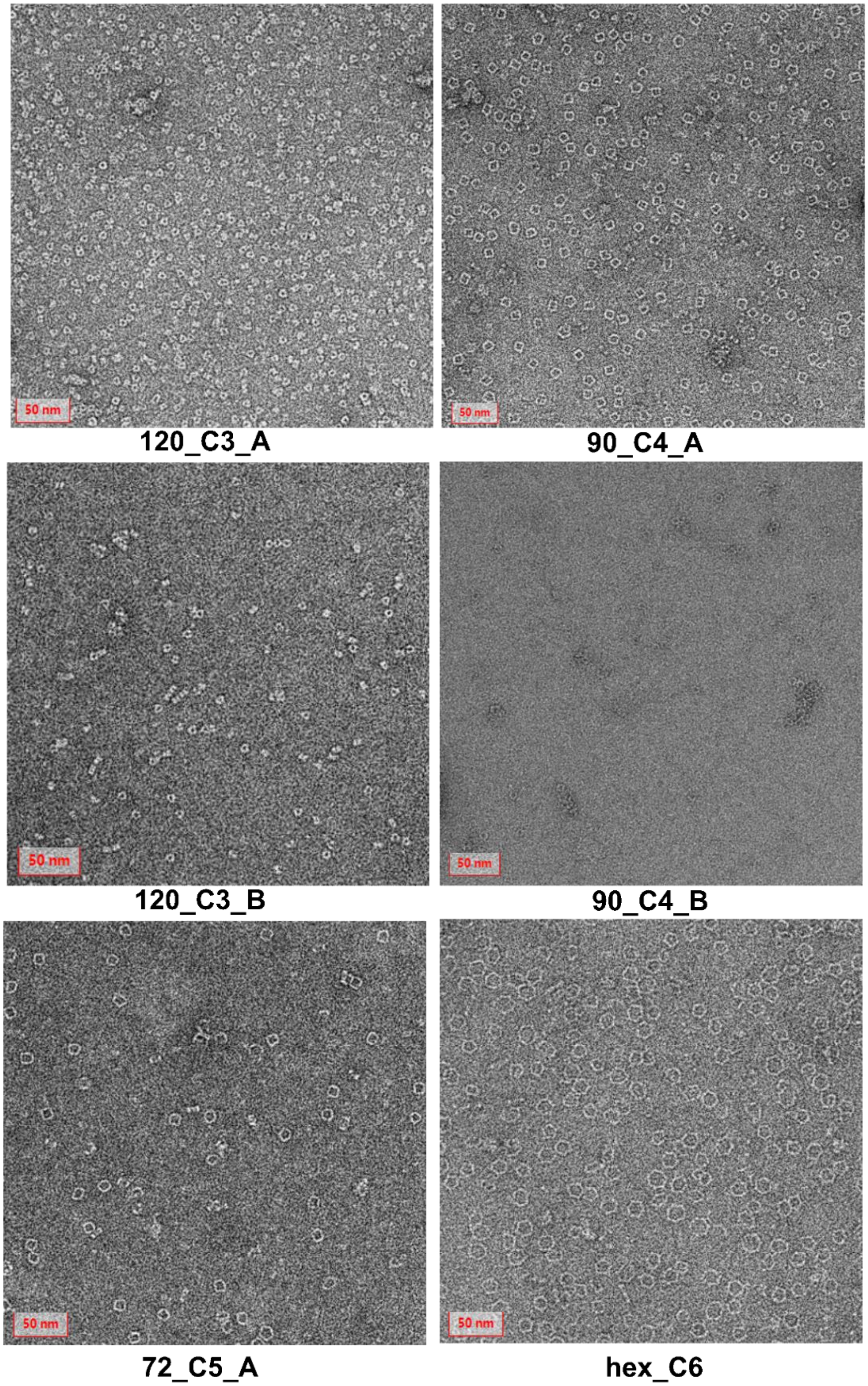

Fig. S38. Wider fields of view in ns-EM to show distribution of polygonal design particles

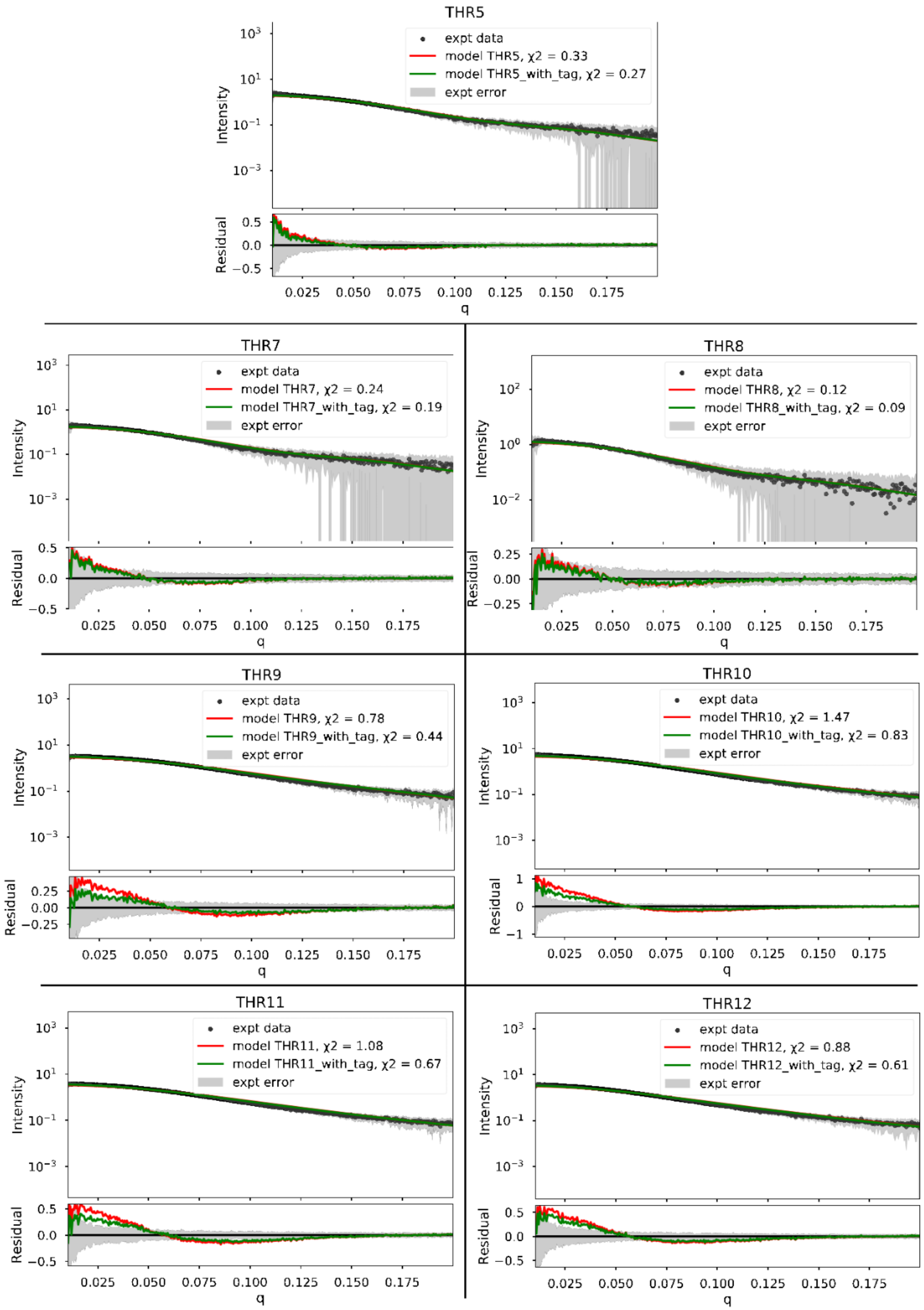

Fig. S39. SAXS data plots for tall-helix THR designs made with Rosetta FastDesign

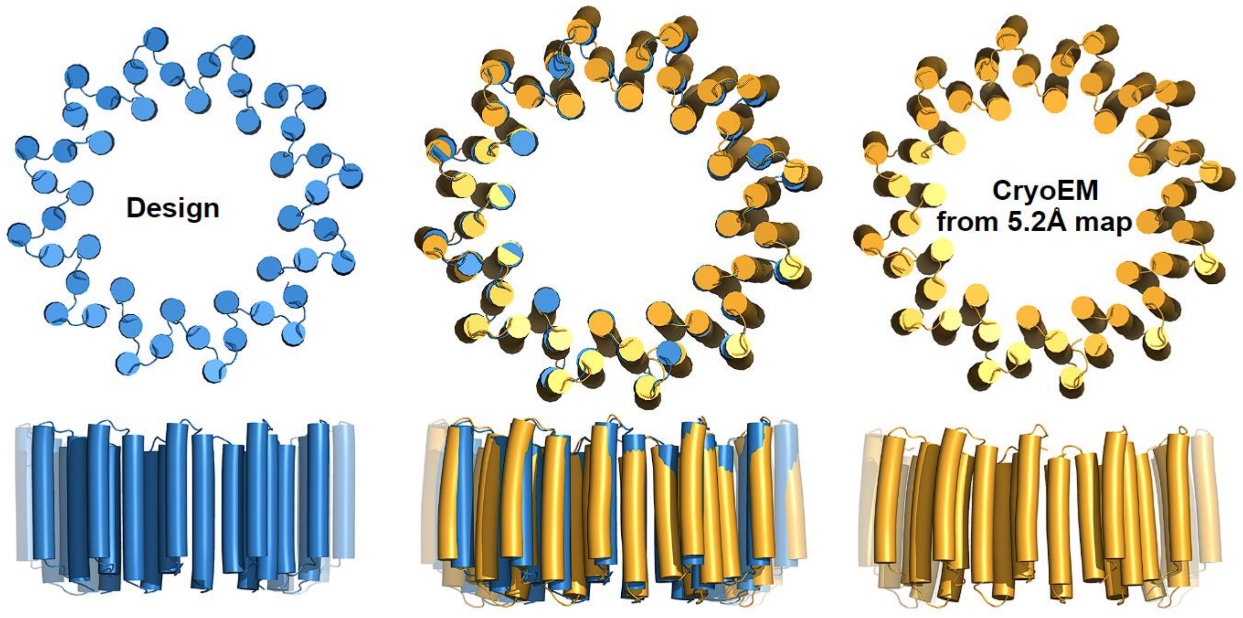

Fig. S40. Comparison of helix positions for *R12B* design model and experimental model from cryo-EM map

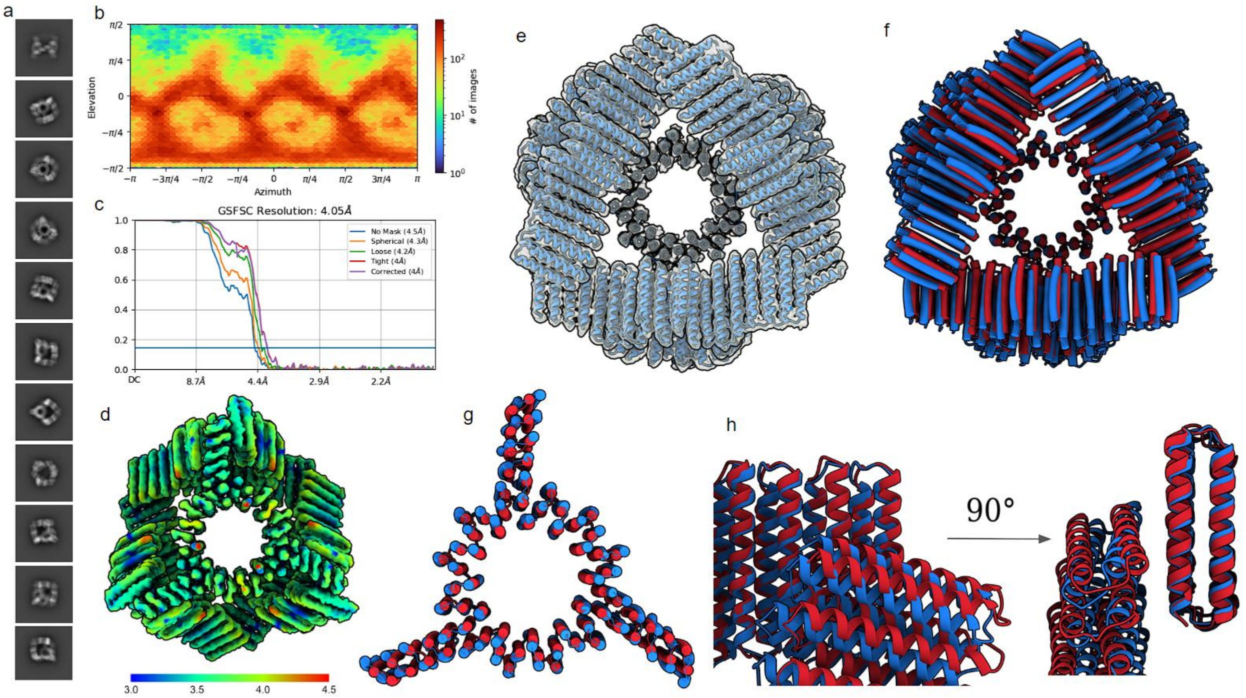

Fig. S41. Details of cryo-EM model built for *cage_T3_101*

a, Representative 2D class averages **b**, Orientational distribution plot demonstrating full angular sampling **c**, Global FSC. **d**, CryoEM local resolution map **e,** Refined 4.05Å structure fit into density **f,g,h** Computationally predicted model (red) cryo-EM structure (blue) **f**, Overlay of the design model with the cryo-EM structure, diameter of cryo-EM structure is expanded vs computational model, alignment done in chimera with fit to map **g**, Alignment of single side of 4 sided nanocage, inner diameter of the ring is expanded and arms are bent slightly in cryo-EM structure vs computational model, aligned in pymol RMSD = 2.788 **h**, Alignment of cryo-EM structure and computational model using one 8 helix hand of the handshake, the interface and angle of contact are both shifted, computational model handshake has an interface area 1113.7 Å^2 vs the cryo-EM structure with an average interface area of 662.8 Å^2 (pisa server).

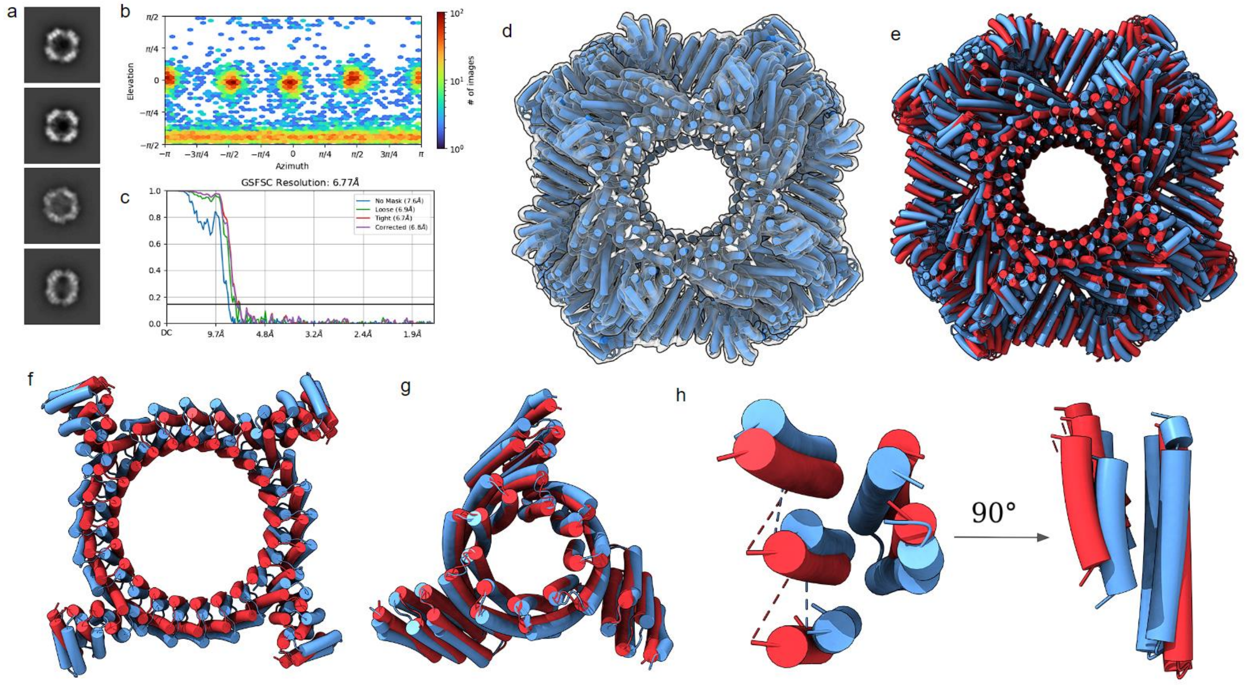

Fig. S42. Details of cryo-EM model built for *cage_O43_129*

a, Representative 2D class averages **b**, Orientational distribution plot demonstrating angular sampling **c**, Global FSC. **d,** Refined 6.77Å cryo-EM model fit into cryo-EM density map .**e,** CryoEM structure (blue) overlaid with the computationally predicted model (red) **f**, Alignment of tetrameric subunit, to map **g**, Alignment of trimetric subunit (RMSD = 1.691) **h**, Alignment of interface between the two subunits (RMSD = 4.247)

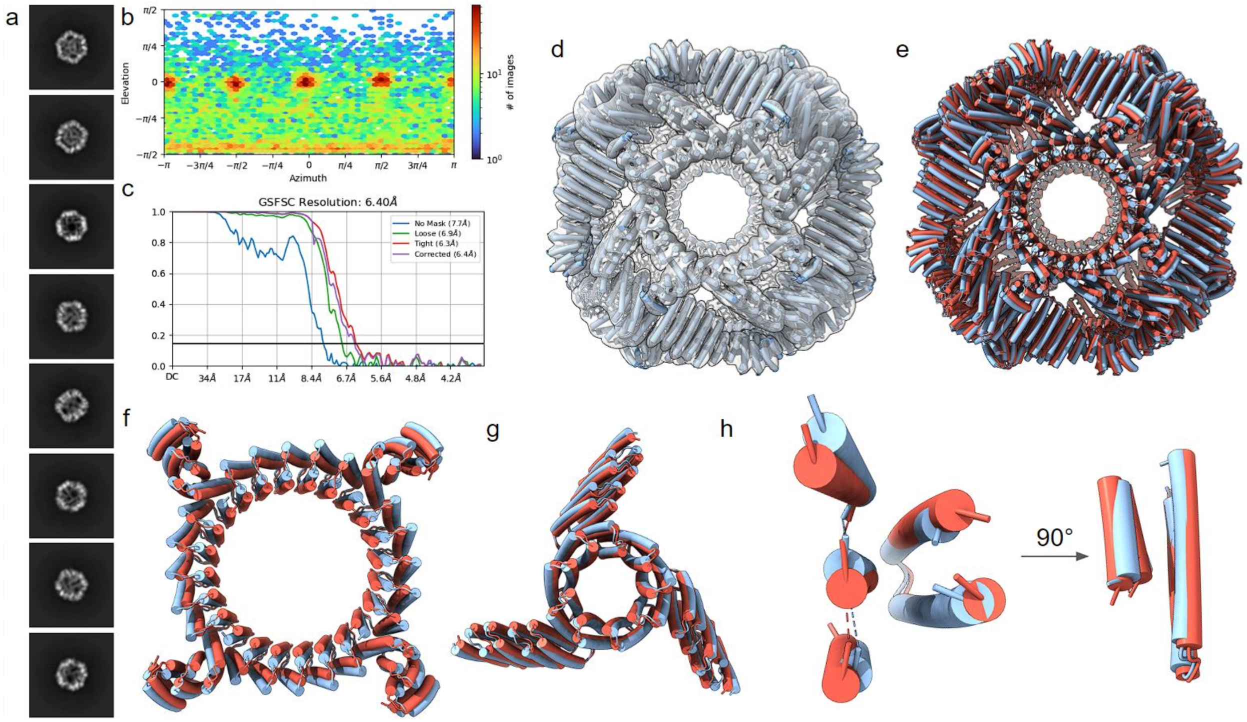

Fig. S43. Details of cryo-EM model built for *cage_O43_129_+4*

a, Representative 2D class averages **b**, Orientational distribution plot demonstrating angular sampling **c**, Global FSC. **d,** Refined 6.44Å cryo-EM model fit into cryo-EM density map .**e,** CryoEM structure (blue) overlaid with the computationally predicted model (red) **f**, Alignment of tetrameric subunit (RMSD 3.503) **g**, Alignment of trimetric subunit (RMSD 2.022) **h**, Alignment of interface between the two subunits (RMSD 0.933)

	**sC4** (EMDB-29974) (PDB 8GEL)	**sC4_+2**	**cage_O3_10** (EMDB-40070)	**cage_O3_10** (EMDB-40071)
**Data collection and processing**				
Microscope	Krios	Arctica	Krios	Krios
Exposure navigation	Imageshift (Leginon)	Imageshift (Leginon)	Imageshift (Leginon)	Imageshift (Leginon)
Magnification	105,000x	36,000x	105,000x	105,000x
Voltage (kV)	300	200	300	300
Detector	Gatan K3	Gatan K3	Gatan K3	Gatan K3
Electron exposure (e–/Å^2)	47.07	56.8	58.8	58.8
Exposure time (s)	1.6	2.8	2	2
Defocus range (μm)	0.8 – 2.0	1.1 - 2.9	0.6 - 2.2	0.6 - 2.2
Pixel size (Å)	0.4124	0.548	0.4124	0.4124
Number of frames	40	40	50	50
Number of micrographs	3,850	207	4,262	4,262
Software for processing	CryoSPARC, Relion	CryoSPARC	CryoSPARC	CryoSPARC
Initial particle images (no.)	3,781,336	51,637	2,000,418	2,000,418
Final particle images (no.)	378,829	34,762	49,466	49,466
Box size (pixels)	256	256	512	512
Symmetry imposed	C4	N/A	C1	O
Map resolution (Å) for FSC threshold=0.143 (unmasked / masked)	4.06 / 3.91	N/A	8.2 / 7.4	7.0 / 6.0
Sphericity from 3DFSC (unmasked/masked)	0.851 / 0.952	N/A	0.660 / 0.737	0.958 / 0.971
**Refinement**		N/A	N/A	N/A
Initial model used	Computational model	N/A	N/A	N/A
Map CC (mask)	0.791	N/A	N/A	N/A
Map CC (volume)	0.764	N/A	N/A	N/A
Map CC (peaks	0.648	N/A	N/A	N/A
Model resolution (Å)				
FSC threshold	4.0	N/A	N/A	N/A
Model resolution range (Å)	3.8 - 4.5	N/A	N/A	N/A
Map sharpening B factor (Å^2)	-224.3	N/A	N/A	N/A
Model composition				
Non-hydrogen atoms	15,566	N/A	N/A	N/A
Protein residues	1,992	N/A	N/A	N/A
Ligands	0	N/A	N/A	N/A

ADP *B* factors (Å^2)				
Protein (min/max/mean)	24.04/88.07/45.86	N/A	N/A	N/A
Ligand	NA	N/A	N/A	N/A
R.m.s. deviations				
Bond lengths (Å)	0.007	N/A	N/A	N/A
Bond angles (°)	0.861	N/A	N/A	N/A
Validation				
MolProbity score	1.81	N/A	N/A	N/A
Clashscore	20.54	N/A	N/A	N/A
Poor rotamers (%)	0.00	N/A	N/A	N/A
C-beta deviations (%)	0.00	N/A	N/A	N/A
Ramachandran plot				
Favored (%)	98.38	N/A	N/A	N/A
Allowed (%)	1.62	N/A	N/A	N/A
Disallowed (%)	0.00	N/A	N/A	N/A

Table S1. Cryo-EM data collection, processing, and refinement parameters for a subset of designs (I).

	**cage_T3_5** (EMDB-40075)	**cage_T3_5** (EMDB-40074)	**cage_T3_5** (EMDB-40073)	**cage_T3_5** (EMDB-40072)
**Data collection and processing**				
Microscope	Krios	Krios	Krios	Krios
Exposure navigation	Imageshift (Leginon)	Imageshift (Leginon)	Imageshift (Leginon)	Imageshift (Leginon)
Magnification	105,000x	105,000x	105,000x	105,000x
Voltage (kV)	300	300	300	300
Detector	Gatan K3	Gatan K3	Gatan K3	Gatan K3
Electron exposure (e–/Å^2)	58.8	58.8	58.8	58.8
Exposure time (s)	2	2	2	2
Defocus range (μm)	0.6 - 2.2	0.6 - 2.2	0.6 - 2.2	0.6 - 2.2
Pixel size (Å)	0.4124	0.4124	0.4124	0.4124
Number of frames	50	50	50	50
Number of micrographs	5,854	5,854	5,854	5,854
Software for processing	CryoSPARC	CryoSPARC	CryoSPARC	CryoSPARC
Initial particle images (no.)	959,145	959,145	959,145	959,145
Final particle images (no.)	144,976	144,976	64,560	52,363
Box size	360	360	360	360
Symmetry imposed	C1	T	C1 (1 chain missing)	C1 (1 trimer missing)
Map resolution (Å) for FSC threshold 0.143 (unmasked / masked)	6.5 / 4.3	4/ 3.6	7.7 / 6.1	7.9 / 6.5
Map sharpening B factor (Å)	none	-165	none	none
Sphericity from 3DFSC (unmasked/masked)	0.965 / 0.967	0.840 / 0.989	0.658 / 0.912	0.635 / 0.751

Table S2. Cryo-EM data collection and processing parameters for a subset of designs (II).

	cage_T3_5_+2 (EMDB-40076)	cage_T3_5_+6	tC3_A
**Data collection and processing**			
Microscope	Krios	Arctica	Krios
Exposure navigation	Imageshift (Leginon)	Imageshift (Leginon)	Imageshift (Leginon)
Magnification	105,000x	36,000x	105,000x
Voltage (kV)	300	200	300
Detector	Gatan K3	Gatan K3	Gatan K3
Electron exposure (e–/Å^2)	58.8	56.8	46.77
Exposure time (s)	2	2.8	1.6
Defocus range (μm)	0.6 - 2.2	0.6-2.4	0.9 - 2.5
Pixel size (Å)	0.4124	0.548	0.4124
Number of frames	50	40	40
Number of micrographs	19,358	90	438
Software for processing	cryoSPARC	Relion, cryoSPARC	Relion, cryoSPARC
Initial particle images (no.)	5,440,898	58,853	835,272
Final particle images (no.)	166,363	6,863	307,909
Box size	360	360	180
Symmetry imposed	C1	N/A	N/A
Map resolution (Å) for FSC threshold 0.143 (unmasked / masked)	7.3 / 6.7	N/A	N/A
Map sharpening B factor (Å)	none	N/A	N/A
Sphericity from 3DFSC (unmasked/masked)	0.911 / 0.912	N/A	N/A

Table S3. Cryo-EM data collection and processing parameters for a subset of designs (III).

	**cage_O43_129** (EMDB-42906) (PDB 8V2D)	**cage_O43_129_+4** (EMDB-42944) (PDB 8V3B)	**cage_O43_129_+8** (EMDB-42031) (PDB 8V2D)	**cage_O4_34** (EMDB-29915) (PDB 8V2D)
**Data collection and processing**				
Microscope	Glacios	Krios	Glacios	Tundra
Exposure navigation	Imageshift (SerialEM)	Imageshift (SerialEM)	Imageshift (SerialEM)	Imageshift (EPU)
Magnification	45,000x	105,000x	45,000x	110,000x
Voltage (kV)	200	200	200	100
Detector	Gatan K3	Gatan K3	Gatan K3	CETA-F
Electron-exposure (e–/Å^2)	50	52	50	41,5
Exposure time (s)	5	5	5	1.5
Defocus range (μm)	0.8 - 1.8	0.8 - 1.8	0.8 - 1.8	0.5 - 2.2
Pixel size (Å)	0.89	0.84	0.89	1.248
Number of frames	99	100	99	21
Number of micrographs	922	6,851	2,990	4,550
Software for processing	cryoSPARC	cryoSPARC	cryoSPARC	cryoSPARC
Initial particle images (no.)	32,135	23,522	8,251	248,248
Final particle images (no.)	13,409	16,878	5,420	59,904
Box size	546	800	900>380 downsampled	380
Symmetry imposed	O	O	O	O
Map resolution (Å) for FSC threshold=0.143 (unmasked / masked)	7.6 / 6.77	7.6 / 6.4	14 / 11.9	7.7 / 7.5
**Refinement**			N/A	N/A
Initial model used	Computational model	Computational model	N/A	N/A
Map-sharpening B factor (Å^2)	DeepEMhancer	DeepEMhancer	N/A	N/A
Map resolution (Å) for FSC threshold=0.143 (unmasked / masked)	7.6 / 6.77	7.6 / 6.4	N/A	N/A
Model composition				
Non-hydrogen atoms	73808	86736	N/A	N/A
Protein residues	14904	17520	N/A	N/A
Ligands	0	0	N/A	N/A

B factors (Å^2)				
Protein	DeepEMhancer	DeepEMhancer	N/A	N/A
Ligand	NA	N/A	N/A	N/A
R.m.s. deviations				
Bond-lengths (Å)	0.009	0.005	N/A	N/A
Bond angles (°)	1.394	0.612	N/A	N/A
Validation				
MolProbity	0.50	0.98	N/A	N/A
Clashscore	0	0	N/A	N/A
Poor-rotamers %	0	0	N/A	N/A
C-beta deviations (%)	N/A	N/A	N/A	N/A
Ramachandran plot				
Favored (%)	99.20	99.69	N/A	N/A
Allowed (%)	0.80	0.31	N/A	N/A
Disallowed(%)	0.00	N/A	N/A	N/A

Table S4. Cryo-EM data collection, processing, and refinement parameters for a subset of designs (IV).

	**cage_O4_34_+4** (EMDB-41907)	**T3_101** (EMDB-41364) (PDB 8TL7)	**R12B** (EMDB-43318)	**strut_C6_21** (EMDB-29893)
**Data collection and processing**				
Microscope	Glacios	Glacios	Glacios	Glacios
Exposure navigation	Imageshift (SerialEM)	Imageshift (SerialEM)	Imageshift (SerialEM)	Image shift (SerialEM)
Magnification	45,000x	45,000x	45,000x	36,000x
Voltage (kV)	200	200	200	200
Detector	Gatan K3	Gatan K3	Gatan K3	Gatan K3
Electron-exposure (e–/$Å^2$)	50	50	50	50
Exposure time (s)	5	5	5	10
Defocus range (μm)	0.8 - 1.8	0.8 - 1.8	0.8 - 1.2	0.8 - 1.8
Pixel size (Å)	0.89	0.89	0.445	0.89
Number of frames	99	99	99	50
Number of micrographs	3,837	3,696	12,944	1,116
Software for processing	cryoSPARC	cryoSPARC	cryoSPARC, Relion 4.0	cryoSPARC
Initial particle images (no.)	302.911	1,068,087	255,056	1,479,115
Final particle images (no.)	124,075	266,100	68,331	37,105
Box size	706	490	300	450
Symmetry imposed	O	T	C12	C6
Map resolution (Å) for FSC threshold=0.143 (unmasked / masked)	6.67 / 5.64	4.33 / 3.99	5.6 / 5.2	6.7 / 5.0
**Refinement**	N/A	N/A	N/A	N/A
Initial model used	N/A	Computational model	N/A	N/A
Map sharpening B factor ($Å^2$)	N/A	DeepEMhancer	N/A	N/A
Map resolution (Å) for FSC threshold=0.143 (unmasked / masked)	N/A	4.33 / 3.99	N/A	N/A
Model composition				
Non-hydrogen atoms	N/A	50433	N/A	N/A
Protein residues	N/A	8328	N/A	N/A
Ligands	N/A	0	N/A	N/A
B factors ($Å^2$)				
Protein	N/A	DeepEMhancer	N/A	N/A
Ligand	N/A	N/A	N/A	N/A
R.m.s. deviations				
Bond-lengths(Å)	N/A	.002	N/A	N/A

Bond angles (°)	N/A	.517	N/A	N/A
Validation				
MolProbity	N/A	0.98	N/A	N/A
Clashscore	N/A	0	N/A	N/A
Poor-rotamers %	N/A	0	N/A	N/A
C-beta deviations (%)	N/A	N/A	N/A	N/A
Ramachandran plot				
Favored (%)	N/A	99.69	N/A	N/A
Allowed (%)	N/A	0.31	N/A	N/A
Disallowed (%)	N/A	0.00	N/A	N/A

Table S5. Cryo-EM data collection, processing, and refinement parameters for a subset of designs (V).

	THR1 (8G9J)	THR2 (8G9K)	THR5 (8GA7)	THR6 (8GA6)
**Data collection**				
Space group	*P* 4$_1$ 2$_1$ 2	*P* 2$_1$ 2$_1$ 2$_1$	*P* 2$_1$ 2$_1$ 2$_1$	*P* 2$_1$ 2$_1$
Cell dimensions				
a, *b*, *c* (Å)	49.70, 49.70, 129.26	46.39, 61.19, 149.02	46.41, 65.06, 135.28	34.96, 120.42, 52.27
α, β, γ (°)	90, 90, 90	90, 90, 90	90, 90, 90	90, 105.65, 90
Resolution (Å)	46.39 - 2.5 (2.75 - 2.5)	56.61 - 2.48 (2.56 - 2.48)	38.27 - 2.93 (3.08 - 2.93)	46.44 - 2.5 (2.58 - 2.5)
R_{merge}	0.0699 (0.431)	0.063 (0.123)	0.204 (1.178)	0.137 (0.778)
R_{pim}	0.0698 (0.4314)	0.025 (0.491)	0.079 (0.439)	0.084 (0.463)
$I / \sigma I$	6.62 (1.20)	17.19 (6.68)	7.79 (2.61)	5.24 (1.69)
$CC_{1/2}$	0.999 (0.870)	0.999 (0.999)	0.997 (0.939)	0.982 (0.722)
Completeness (%)	98.64 (95.58)	98.91 (98.79)	98.34 (98.93)	97.98 (95.07)
Redundancy	1.7 (1.3)	7.4 (7.6)	7.5 (7.9)	3.7 (3.8)
**Refinement**				
Resolution (Å)	46.39 - 2.5 (2.75 - 2.5)	56.61 - 2.48 (2.56 - 2.48)	38.27 - 2.93 (3.08 - 2.93)	46.44 - 2.5 (2.58 - 2.5)
No. reflections	6005 (1404)	15526 (1388)	9149 (1290)	14095 (1330)
R_{work} / R_{free}	0.2333 (0.2987) / 0.2842 (0.3763)	0.2642 (0.3305) / 0.3010 (0.3548)	0.2756 (0.3005) / 0.3144 (0.3387)	0.2663 (0.2739) / 0.3195 (0.3633)
No. atoms				
Protein	1486	2965	3438	3476
Ligand/ion	0	0	0	0
Water	13	39	0	98
B-factors				
Protein	46.72	78.57	58.84	43.01
Ligand/ion	NA	NA	NA	NA
Water	40.71	73.05	NA	37.67
Rama Favored/allowed Outlier (%)	98.49/ 1.51/ 0.00	97.99/ 0.76/ 0.25	97.60/ 2.00/ 0.40	98.30/ 1.06/ 0.64
R.m.s. deviations				
Bond lengths (Å)	0.003	0.001	0.002	0.002
Bond angles (°)	0.520	0.400	0.400	0.370

Table S6. Crystallographic data collection and refinement
